# Trends in prevalence of blindness and distance and near vision impairment over 30 years: an analysis for the Global Burden of Disease Study

**DOI:** 10.1016/S2214-109X(20)30425-3

**Published:** 2020-12-01

**Authors:** GBD 2019 Blindness and Vision Impairment Collaborators, Rupert Bourne, Jaimie D Steinmetz, Seth Flaxman, Paul Svitil Briant, Hugh R Taylor, Serge Resnikoff, Robert James Casson, Amir Abdoli, Eman Abu-Gharbieh, Ashkan Afshin, Hamid Ahmadieh, Yonas Akalu, Alehegn Aderaw Alamneh, Wondu Alemayehu, Ahmed Samir Alfaar, Vahid Alipour, Etsay Woldu Anbesu, Sofia Androudi, Jalal Arabloo, Aries Arditi, Malke Asaad, Eleni Bagli, Atif Amin Baig, Till Winfried Bärnighausen, Maurizio Battaglia Parodi, Akshaya Srikanth Bhagavathula, Nikha Bhardwaj, Pankaj Bhardwaj, Krittika Bhattacharyya, Ali Bijani, Mukharram Bikbov, Michele Bottone, Tasanee Braithwaite, Alain M Bron, Zahid A Butt, Ching-Yu Cheng, Dinh-Toi Chu, Maria Vittoria Cicinelli, João M Coelho, Baye Dagnew, Xiaochen Dai, Reza Dana, Lalit Dandona, Rakhi Dandona, Monte A Del Monte, Jenny P Deva, Daniel Diaz, Shirin Djalalinia, Laura E Dreer, Joshua R Ehrlich, Leon B Ellwein, Mohammad Hassan Emamian, Arthur G Fernandes, Florian Fischer, David S Friedman, João M Furtado, Abhay Motiramji Gaidhane, Shilpa Gaidhane, Gus Gazzard, Berhe Gebremichael, Ronnie George, Ahmad Ghashghaee, Mahaveer Golechha, Samer Hamidi, Billy Randall Hammond, Mary Elizabeth R Hartnett, Risky Kusuma Hartono, Simon I Hay, Golnaz Heidari, Hung Chak Ho, Chi Linh Hoang, Mowafa Househ, Segun Emmanuel Ibitoye, Irena M Ilic, Milena D Ilic, April D Ingram, Seyed Sina Naghibi Irvani, Ravi Prakash Jha, Rim Kahloun, Himal Kandel, Ayele Semachew Kasa, John H Kempen, Maryam Keramati, Moncef Khairallah, Ejaz Ahmad Khan, Rohit C Khanna, Mahalaqua Nazli Khatib, Judy E Kim, Yun Jin Kim, Sezer Kisa, Adnan Kisa, Ai Koyanagi, Om P Kurmi, Van Charles Lansingh, Janet L Leasher, Nicolas Leveziel, Hans Limburg, Marek Majdan, Navid Manafi, Kaweh Mansouri, Colm McAlinden, Seyed Farzad Mohammadi, Abdollah Mohammadian-Hafshejani, Reza Mohammadpourhodki, Ali H Mokdad, Delaram Moosavi, Alan R Morse, Mehdi Naderi, Kovin S Naidoo, Vinay Nangia, Cuong Tat Nguyen, Huong Lan Thi Nguyen, Kolawole Ogundimu, Andrew T Olagunju, Samuel M Ostroff, Songhomitra Panda-Jonas, Konrad Pesudovs, Tunde Peto, Zahiruddin Quazi Syed, Mohammad Hifz Ur Rahman, Pradeep Y Ramulu, Salman Rawaf, David Laith Rawaf, Nickolas Reinig, Alan L Robin, Luca Rossetti, Sare Safi, Amirhossein Sahebkar, Abdallah M Samy, Deepak Saxena, Janet B Serle, Masood Ali Shaikh, Tueng T Shen, Kenji Shibuya, Jae Il Shin, Juan Carlos Silva, Alexander Silvester, Jasvinder A Singh, Deepika Singhal, Rita S Sitorus, Eirini Skiadaresi, Vegard Skirbekk, Amin Soheili, Raúl A R C Sousa, Emma Elizabeth Spurlock, Dwight Stambolian, Biruk Wogayehu Taddele, Eyayou Girma Tadesse, Nina Tahhan, Md Ismail Tareque, Fotis Topouzis, Bach Xuan Tran, Ravensara S Travillian, Miltiadis K Tsilimbaris, Rohit Varma, Gianni Virgili, Ya Xing Wang, Ningli Wang, Sheila K West, Tien Y Wong, Zoubida Zaidi, Kaleab Alemayehu Zewdie, Jost B Jonas, Theo Vos, Vision Loss Expert Group of the Global Burden of Disease Study

## Abstract

**Background:**

To contribute to the WHO initiative, VISION 2020: The Right to Sight, an assessment of global vision impairment in 2020 and temporal change is needed. We aimed to extensively update estimates of global vision loss burden, presenting estimates for 2020, temporal change over three decades between 1990–2020, and forecasts for 2050.

**Methods:**

We did a systematic review and meta-analysis of population-based surveys of eye disease from January, 1980, to October, 2018. Only studies with samples representative of the population and with clearly defined visual acuity testing protocols were included. We fitted hierarchical models to estimate 2020 prevalence (with 95% uncertainty intervals [UIs]) of mild vision impairment (presenting visual acuity ≥6/18 and <6/12), moderate and severe vision impairment (<6/18 to 3/60), and blindness (<3/60 or less than 10° visual field around central fixation); and vision impairment from uncorrected presbyopia (presenting near vision <N6 or <N8 at 40 cm where best-corrected distance visual acuity is ≥6/12). We forecast estimates of vision loss up to 2050.

**Findings:**

In 2020, an estimated 43·3 million (95% UI 37·6–48·4) people were blind, of whom 23·9 million (55%; 20·8–26·8) were estimated to be female. We estimated 295 million (267–325) people to have moderate and severe vision impairment, of whom 163 million (55%; 147–179) were female; 258 million (233–285) to have mild vision impairment, of whom 142 million (55%; 128–157) were female; and 510 million (371–667) to have visual impairment from uncorrected presbyopia, of whom 280 million (55%; 205–365) were female. Globally, between 1990 and 2020, among adults aged 50 years or older, age-standardised prevalence of blindness decreased by 28·5% (–29·4 to −27·7) and prevalence of mild vision impairment decreased slightly (–0·3%, −0·8 to −0·2), whereas prevalence of moderate and severe vision impairment increased slightly (2·5%, 1·9 to 3·2; insufficient data were available to calculate this statistic for vision impairment from uncorrected presbyopia). In this period, the number of people who were blind increased by 50·6% (47·8 to 53·4) and the number with moderate and severe vision impairment increased by 91·7% (87·6 to 95·8). By 2050, we predict 61·0 million (52·9 to 69·3) people will be blind, 474 million (428 to 518) will have moderate and severe vision impairment, 360 million (322 to 400) will have mild vision impairment, and 866 million (629 to 1150) will have uncorrected presbyopia.

**Interpretation:**

Age-adjusted prevalence of blindness has reduced over the past three decades, yet due to population growth, progress is not keeping pace with needs. We face enormous challenges in avoiding vision impairment as the global population grows and ages.

**Funding:**

Brien Holden Vision Institute, Fondation Thea, Fred Hollows Foundation, Bill & Melinda Gates Foundation, Lions Clubs International Foundation, Sightsavers International, and University of Heidelberg.

## Introduction

VISION 2020: The Right to Sight, a joint global initiative for the elimination of avoidable blindness between WHO and the International Agency for the Prevention of Blindness, has galvanised efforts to systematically eliminate avoidable blindness.^1^, ^2^ Furthermore, the adoption of the resolution *Towards universal eye health: a global action plan 2014–2019* by the World Health Assembly renewed ideals and strategies for Member States to amplify initiatives to prevent vision impairment and promote low vision rehabilitation in their populations.^3^

The Vision Loss Expert Group (VLEG) populates and curates the Global Vision Database, a continuously updated, comprehensive, online database storing worldwide ophthalmic epidemiological information. In a previous report, we estimated that in 2015, 36·0 million people were blind, 217 million had moderate and severe vision impairment, 188 million had mild vision impairment, and 667 million additional people (aged ≥50 years) had vision impairment from uncorrected presbyopia.^4^ These 2015 estimates were used for the *World report on vision*, published by WHO in 2019.^5^ We subsequently provided country-specific data online, searchable by level of vision impairment, age, and sex.

**Research in context**

**Evidence before this study**

The Vision Loss Expert Group (VLEG) previously published global, regional, and country-level estimates of mild or more severe distance vision impairment from 1990 to 2015. They found an overall decrease in age-standardised prevalence of distance vision but an increase in total people with vision loss. In collaboration with the VLEG, the Global Burden of Disease Study (GBD) has produced annual updates of estimates of vision impairment, most recently in 2019. An important contribution to the WHO initiative, VISION 2020: The Right to Sight, these studies are unique in providing granular estimates that are sex, age, and country specific, and also assess temporal trends.

**Added value of this study**

This updated assessment of global vision impairment contributes to VISION 2020: The Right to Sight by providing up-to-date global and regional sex-specific and age-specific estimates of vision impairment, analyses of how vision impairment rates and cases have changed over time, and projections by region to 2050. The current study also allows us to assess the World Health Assembly 2013 resolution to decrease avoidable moderate or worse distance vision impairment by 25% from 2010 to 2019. Our results suggest that total cases of moderate or worse distance vision have increased since 2010, and therefore, in terms of crude prevalence, the target was not reached.

**Implications of all the available evidence**

Despite a reduction in global age-standardised prevalence of blindness, the crude prevalence is increasing, and with an ageing and growing population, vision impairment remains an urgent and increasingly important public health priority. We are currently investigating the causes of vision impairment, and more directly assessing temporal changes in avoidable vision loss.

As countries progress on the spectrum of socioeconomic development and life expectancies increase, the disease burden is shifting from communicable conditions to non-communicable and age-related conditions. The blinding ophthalmic conditions that are manifesting in this so-called epidemiological transition^6^ include age-related cataract,^7^ age-related macular degeneration,^8^ glaucoma,^9^ and diabetic retinopathy.^10^ Vision impairment and blindness are associated with reduced economic, educational, and employment opportunities,^11^, ^12^, ^13^, ^14^ and increased risk of death.^15^, ^16^ Furthermore, in older age, vision impairment not only significantly affects quality of life^17^ (eg, the association between vision impairment and depression^18^, ^19^, ^20^) but also amplifies comorbidities such as cognitive impairment^21^ and risk of falls.^22^

Our 2015 report concentrated on blindness and moderate and severe vision impairment, with little data pertaining to mild vision impairment and vision impairment from uncorrected presbyopia. Since then, in collaboration with the Global Burden of Disease Study (GBD), the Global Vision Database has been extensively updated and revised with increased granularity of the available worldwide ophthalmic epidemiological information, permitting a more precise estimate of the global vision loss burden in 2020 and assessment of temporal changes. Additionally, we can describe vision loss in terms of years lived with disability (YLDs).

## Methods

### Input data

Preparation of data included first a systematic review of published population-based studies of vision impairment and blindness by the VLEG, that also included grey literature sources. Eligible studies from this review were then combined with data from Rapid Assessment of Avoidable Blindness (RAAB) studies by VLEG and finally data from the US National Health and Nutrition Examination survey and the WHO Study on Global Ageing and Adult Health were contributed by the GBD team. These stages are explained in more detail as follows.

In this study, we used population-representative studies as data sources for modelling of vision impairment; more detailed methods are in the appendix (pp 3–25). The VLEG serially systematically reviewed literature published between Jan 1, 1980, and Oct 1, 2018, by commissioning the York Health Economics Consortium (University of York, York, UK) to search Embase, SciELO, MEDLINE, WHO Library Database, and OpenGrey, and additional grey literature sources (a detailed search strategy is given in the appendix [pp 4–24]). After title and abstract screening, abstracts were sent to regional committees of VLEG members to assess quality and make final inclusion decisions. In total, VLEG identified 137 studies and extracted data from 70 studies in their 2010 review, and a further 67 studies in their 2014–18 review. Studies were primarily national and subnational cross-sectional surveys. We included RAAB surveys, which sampled individuals aged 50–99 years in low-income and middle-income settings. Additionally, the VLEG commissioned the preparation of 5-year age-disaggregated RAAB data from the RAAB repository. We included studies that met the following criteria: visual acuity data had to be measured using a test chart that could be mapped to the Snellen scale, and the sample had to be representative of the population. Self-report of vision loss was excluded. We used International Classification of Diseases 11th edition criteria for vision loss, as used by WHO, which categorises people according to vision in the better eye on presentation, in which mild vision loss is defined as a visual acuity of 6/18 or better but less than 6/12, moderate vision loss as a visual acuity of 6/60 or better but less than 6/18, severe vision loss as a visual acuity of 3/60 or better but less than 6/60 (hence our definition of moderate and severe vision loss is <6/18 to 3/60), and blindness as a visual acuity of less than 3/60 or less than 10° visual field around central fixation (although the visual field definition is rarely used in population-based eye surveys). Vision impairment from uncorrected presbyopia was defined as presenting near vision of worse than N6 or N8 at 40 cm when best-corrected distance visual acuity was 6/12 or better. This definition was used to avoid double counting individuals with both distance and near vision impairment associated with non-refractive causes.^4^ We included studies that measured either presenting vision loss, where visual acuity was measured using the usual corrective lenses an individual arrived wearing, or best-corrected vision loss, where correction using a lens or pinhole addressed any refractive error.

The data identification process is summarised in the appendix (pp 3–25, 93–100, 102).

### Data preparation

First, we separated raw data into datasets including so-called vision-loss envelopes for all-cause mild, moderate, and severe vision loss, and blindness. Studies that only reported male and female sex information combined were split into male-specific and female-specific datapoints by identifying within-study datapoints matched by age, year, and location that reported data for male and female prevalence separately, and then we used the log ratio of female to male prevalence from these studies as input data into a mixed-effects meta-regression tool developed by the Institute for Health Metrics and Evaluation (IHME) called MR-BRT (meta regression; Bayesian; regularised; trimmed). This tool was developed in part to allow for the ability to propagate between-study heterogeneity as part of uncertainty adjustments, and to allow trimming of outlier input data. A detailed description of MR-BRT has been published elsewhere.^23^ We used results from this model and demographic data on population by location to determine male and female prevalence of vision impairment. Next, we used MR-BRT to adjust non-reference data to the reference definition of presenting vision data that fit within the WHO severity categories using data from studies that did not involve RAAB methods. We split data that spanned thresholds (either prevalence data for moderate and severe vision loss combined, or severe vision loss and blindness combined) into reference severity groups (moderate vision loss, severe vision loss, blindness) using a log ratio meta-regression with a cubic spline on age with linear tails. The input data for this meta-regression came from studies that provided matched age, year, sex, and location data for each severity level (eg, moderate vision loss and severe vision loss separately). If input data were collected for age ranges of greater than 25 years, these data were split into 5-year age bins. More detailed information on age-splitting and adjustment of best-corrected visual acuity data to the reference definitions is in the appendix (pp 25–26).

### Disease Modelling Meta-Regression 2.1 modelling

Disease Modelling Meta-Regression (DisMod-MR) 2.1 modelling is described in detail elsewhere.^24^ Briefly, this is a Bayesian mixed-effects meta-regression tool developed for GBD non-fatal disease modelling. It is based on a compartmental model with age integration in which individuals can move from a state of health to disease, and either back to health or to death. For vision modelling, no fatal component exists and estimates are made for prevalence only. For DisMod modelling, we used all input data from all locations in a mixed-effects non-linear model for a global estimate of disease burden. We used model outputs (global fit plus fixed effects plus random effects) in a cascade as a prior for estimates in the seven GBD super-regions, which in turn were used as priors for regional estimates, and then country estimates, and finally subnational estimates for a subset of 21 countries. Final estimates for each geographical level were calculated by aggregation, where the country final estimate was the sum of subnational estimates, the regional final estimate was the sum of country final estimates, and the super-region final estimate was the sum of the regional final estimates. We used the GBD classification of countries and territories by region and super-region, a list of which is in the appendix (pp 28–29). We only present data by region in this Article.

### Modelling and post-processing steps

We ran DisMod-MR 2.1 models for all vision loss severity categories. We used final estimates for vision loss to calculate YLDs on the basis of disability weights assigned to each severity of vision loss, and, finally, adjusted for comorbidity with any causes of non-fatal health loss.^25^ The health states and corresponding disability weights for vision are given in the appendix (p 101). We used YLD data for 2019, because these were the most recent data available.

### Extrapolation, age-standardisation, and forecasting for 2020 and 2050 estimates

Final estimates for GBD 2019 are for the years 1990–2019. To estimate the number of individuals for each severity level for the year 2020, we multiplied 1000 draws of estimated prevalence from GBD 2019 for each location by the 2020 population estimates for each location to calculate the number of affected individuals and 95% uncertainty intervals (UIs) in 2020. We age-standardised our estimates using the GBD standard population (a more detailed description is in the appendix [p 26]).^26^ We forecasted total cases of vision loss by severity for the years 2030, 2040, and 2050. These estimates were made for GBD regions and globally. We generated forecasts of age-specific rates using age-specific prevalence rates for the years 1990, 1995, 2000, 2005, 2010, 2015, and 2019 as input data into a regression analysis with year, region, and age as predictors. We included an interaction term between region and year, and a cubic spline on age. We ran sex-specific regression models 1000 times and used the resulting coefficients to predict rates in the years 2030, 2040, and 2050. We multiplied the predicted rates by forecasted population^26^ for each region to obtain an estimated case number, and then aggregated these estimates to obtain global estimates. Finally, estimates were collapsed to mean and 95% UIs.

### Role of the funding source

The funder of the study had no role in study design, data collection, data analysis, data interpretation, or writing of the report. The corresponding author had full access to all the data in the study and had final responsibility for the decision to submit for publication.

## Results

528 data sources were used to calculate the prevalence of vision loss categories, 243 (46%) of which were RAAB studies. Maps showing the distribution of RAAB and non-RAAB sources by region are in the appendix (p 111). 44 (18%) of 243 RAAB studies were national in scope and the remaining 199 (82%) were subnational. 52 (18%) of 285 non-RAAB studies were nationally representative. Among all sources (both non-RAAB and RAAB), 485 sources included data for blindness, 445 sources included data for moderate and severe vision impairment, 59 sources included data for mild vision impairment, and 25 sources included prevalence of vision impairment from uncorrected presbyopia.

Globally, 43·3 million (95% UI 37·6–48·4) people were estimated to be blind in 2020 (crude prevalence 5·49 [4·76–6·13] per 1000), of whom 23·9 million (55%; 20·8–26·8) were female (table 1; appendix p 105).

**Table 1:** Number of cases and age-standardised prevalence of blindness and moderate and severe vision impairment in 2020 and change since 1990, all ages

	**Blindness**				**Moderate and severe vision impairment**			
	Cases (thousands)		Age-standardised prevalence (per 1000)		Cases (thousands)		Age-standardised prevalence (per 1000)	
	2020	Percentage change 1990–2020	2020	Percentage change 1990–2020	2020	Percentage change in cases 1990–2020	2020	Percentage change 1990–2020
Global	43 300 (37 600 to 48 400)	50·6% (47·8 to 53·4)	5·25 (4·58 to 5·87)	−27·0% (−27·8 to −26·1)	295 000 (267 000 to 325 000)	91·7% (87·6 to 95·8)	35·8 (32·4 to 39·2)	1·1% (0·6 to 1·6)
Andean Latin America	349 (301 to 398)	64·0% (57·7 to 70·3)	5·96 (5·12 to 6·81	−41·6% (−44·2 to −39·5)	2760 (2510 to 3020)	117·0% (110·0 to 125·0)	45·6 (41·4 to 49·9)	−3·9% (−5·6 to −2·2)
Australasia	68·9 (59·1 to 78·8)	71·0% (61·6 to 81·9)	1·48 (1·27 to 1·68)	−19·3% (−22·5 to −16·0)	750 (679 to 821)	78·0% (70·1 to 85·8)	20·3 (18·4 to 22·3)	1·1% (−1·8 to 4·3)
Caribbean	260 (221 to 296)	43·6% (40·6 to 46·8)	4·96 (4·24 to 5·63)	−27·6% (−28·9 to −26·3)	1550 (1410 to 1700)	63·8% (59·1 to 68·2)	30·6 (27·8 to 33·5)	−3·7% (−4·9 to −2·3)
Central Asia	301 (256 to 344)	21·9% (18·2 to 25·2)	4·07 (3·47 to 4·67)	−24·3% (−25·8 to −23·0)	2950 (2660 to 3270)	50·2% (46·8 to 53·5)	36·7 (33·2 to 40·4)	−3·8% (−5·1 to −2·6)
Central Europe	327 (280 to 373)	15·6% (12·3 to 19·5)	1·69 (1·46 to 1·91)	−19·5% (−21·0 to −18·1)	3950 (3490 to 4420)	30·3% (26·6 to 34·0)	21·7 (19·5 to 24·1)	−2·4% (−3·2 to −1·6)
Central Latin America	1270 (1100 to 1420)	76·0% (71·4 to 80·1)	5·09 (4·41 to 5·72)	−38·7% (−39·9 to −37·6)	9840 (8910 to 10800)	115·0% (108·0 to 122·0)	38·4 (34·8 to 42·0)	−5·8% (−6·5 to −5·1)
Central sub-Saharan Africa	287 (247 to 327)	88·1% (82·8 to 93·9)	4·86 (4·14 to 5·54)	−25·8% (−27·7 to −23·5)	2010 (1810 to 2230)	137·0% (131·0 to 142·0)	29·0 (25·8 to 32·3)	−3·0% (−5·0 to −0·8)
East Asia	9090 (7890 to 10300)	71·0% (62·3 to 79·8)	4·66 (4·12 to 5·23)	−25·1% (−27·6 to −22·2)	53 900 (47 800 to 60 400)	144·0% (135·0 to 152·0)	27·8 (24·9 to 30·7)	10·7% (9·5 to 12·0)
Eastern Europe	790 (690 to 890)	−3·4% (−5·7 to −0·8)	2·44 (2·14 to 2·74)	−24·9% (−26·1 to −23·7)	11 100 (9860 to 12 300)	16·3% (14·3 to 18·4)	36·4 (32·8 to 40·1)	−1·7% (−2·5 to −1·0)
Eastern sub-Saharan Africa	1970 (1740 to 2200)	70·9% (68·0 to 73·8)	10·7 (9·24 to 12·0)	−26·7% (−27·6 to −25·7)	7010 (6380 to 7670)	115·0% (112·0 to 118·0)	32·5 (29·4 to 35·6)	−6·9% (−7·9 to −6·0)
High-income Asia Pacific	535 (470 to 597)	43·5% (35·1 to 53·3)	1·45 (1·28 to 1·62)	−26·9% (−28·6 to −25·2)	5340 (4790 to 5860)	69·1% (60·1 to 78·9)	17·7 (16·0 to 19·4)	0·7% (−0·3 to 1·5)
High-income North America	712 (626 to 801)	71·0% (67·5 to 74·9)	1·24 (1·09 to 1·39)	0·3% (−0·6 to 1·2)	7440 (6750 to 8110)	50·2% (46·9 to 53·4)	16·0 (14·4 to 17·5)	0·2% (−0·5 to 0·8)
North Africa and Middle East	3090 (2650 to 3520)	53·0% (49·5 to 56·9)	7·00 (5·93 to 8·02)	−41·5% (−42·7 to −40·3)	21 800 (19 900 to 23 900)	110·0% (104·0 to 115·0)	43·1 (39·1 to 47·2)	−6·1% (−7·3 to −4·5)
Oceania	39·3 (33·8 to 44·6)	76·5% (71·9 to 81·4)	5·54 (4·71 to 6·31)	−23·8% (−25·5 to −21·8)	385 (349 to 425)	123·0% (118·0 to 128·0)	49·3 (44·6 to 54·0)	−1·0% (−3·1 to 1·2)
South Asia	11 900 (10 400 to 13 400)	37·3% (33·5 to 41·2)	8·95 (7·80 to 10·1)	−46·7% (−47·5 to −45·8)	96 200 (86 400 to 107 000)	96·9% (92·7 to 102·0)	64·4 (57·9 to 71·3)	−13·5% (−14·5 to −12·6)
Southeast Asia	5950 (5160 to 6680)	46·1% (43·1 to 49·1)	10·0 (8·71 to 11·3)	−38·9% (−39·9 to −38·0)	28 800 (26 500 to 31 100)	92·4% (87·2 to 97·2)	46·5 (43·0 to 50·1)	−7·0% (−8·8 to −5·0)
Southern Latin America	158 (136 to 180)	32·7% (28·1 to 36·9)	1·93 (1·66 to 2·18)	−28·3% (−30·8 to −26·2)	2120 (1920 to 2310)	56·3% (51·9 to 60·7)	28·1 (25·5 to 30·7)	−2·2% (−4·2 to −0·3)
Southern sub-Saharan Africa	477 (415 to 532)	45·4% (42·0 to 48·6)	8·24 (7·18 to 9·21)	−28·6% (−30·0 to −27·0)	1560 (1410 to 1710)	79·4% (75·2 to 83·4)	23·9 (21·7 to 26·2)	−2·7% (−3·7 to −1·8)
Tropical Latin America	1780 (1560 to 1990)	91·5% (85·4 to 97·6)	7·40 (6·49 to 8·28)	−27·6% (−28·4 to −26·8)	10 300 (9350 to 11 300)	87·8% (80·7 to 95·2)	43·6 (39·5 to 47·6)	−4·5% (−5·3 to −3·8)
Western Europe	1530 (1320 to 1760)	24·0% (19·3 to 29·2)	1·78 (1·55 to 2·00)	−23·9% (−25·0 to −22·7)	15 400 (13 900 to 16 900)	36·5% (32·9 to 40·1)	23·9 (21·6 to 26·2)	−2·1% (−2·8 to −1·3)
Western sub-Saharan Africa	2350 (2070 to 2640)	69·0% (66·1 to 72·4)	11·1 (9·52 to 12·6)	−27·3% (−28·0 to −26·4)	9860 (8940 to 10 800)	124·0% (122·0 to 127·0)	40·6 (36·4 to 45·0)	−3·4% (−4·0 to −2·8)

Data in parentheses are 95% uncertainty intervals. Count data are presented to three significant figures, and percentage changes to one decimal place. Crude prevalence rates are in the appendix (p 105).

In 2020, we estimated that the largest number of people with blindness resided in south Asia, followed by east Asia and southeast Asia (table 1). The crude prevalence of blindness ranged from 1·94 (95% UI 1·70–2·18) cases per 1000 in high-income North America to 8·75 (7·58–9·82) cases per 1000 in southeast Asia (appendix p 105). The age-standardised prevalence of blindness ranged from 1·24 (1·09–1·39) cases per 1000 people in high-income North America to 11·1 (9·52–12·6) per 1000 in western sub-Saharan Africa (table 1).

We estimated that moderate and severe vision impairment affected 295 million (95% UI 267–325) people in 2020 (table 1), with 37·4 (33·9–41·2) cases per 1000 people in the global population, of whom 163 million (55%; 147–179) were female (appendix p 105). Of this category of vision loss, 260 million (232–289) people were moderately vision impaired and 34·8 million (30·7–39·3) were severely vision impaired. The largest number of people with moderate and severe vision impairment also resided in south Asia, followed by east Asia, and southeast Asia (table 1).

We estimated 258 million (95% UI 233–285) people were affected by mild vision impairment in 2020 (crude prevalence 32·7 [29·5–36·2] cases per 1000), of whom 142 million (55%; 128–157) were female (table 2; appendix p 106).

**Table 2:** Number of cases and age-standardised prevalence of mild vision impairment and vision impairment from uncorrected presbyopia in 2020 and change since 1990, all ages, by region

	**Mild vision impairment**				**Vision impairment from uncorrected presbyopia**			
	Cases (in thousands)		Age-standardised prevalence (per 1000)		Cases (in thousands)		Age-standardised prevalence (per 1000)	
	2020	Percentage change 1990–2020	2020	Percentage change 1990–2020	2020	Percentage change 1990–2020	2020	Percentage change 1990–2020
Global	258 000 (233 000 to 285 000)	62·8% (57·5 to 68·1)	32·0 (28·9 to 35·4)	−3·9% (−4·4 to −3·4)	510 000 (371 000 to 667 000)	124·4% (120·1 to 129·2)	59·7 (43·6 to 78·1)	6·3% (4·8 to 7·8)
Andean Latin America	2140 (1930 to 2370)	94·4% (87·1 to 103·0)	35·2 (31·7 to 39·1)	−8·6% (−10·1 to −6·8)	2740 (1980 to 3680)	174·0% (159·0 to 191·0)	47·2 (34·0 to 63·5)	−4·5% (−9·2 to 1·2)
Australasia	427 (382 to 475)	71·8% (64·3 to 79·7)	11·6 (10·4 to 12·9)	−2·9% (−5·4 to 0·3)	324 (219 to 454)	58·5% (21·8 to 94·0)	6·62 (4·42 to 9·31)	−25·1% (−42·7 to −9·5)
Caribbean	1730 (1550 to 1920)	49·1% (44·9 to 53·3)	34·6 (31·2 to 38·3)	−9·7% (−11·0 to −8·4)	2690 (1950 to 3630)	91·2% (82·3 to 99·2)	50·6 (36·7 to 68·0)	−6·7% (−11·0 to −2·6)
Central Asia	2200 (1990 to 2440)	33·7% (30·0 to 37·2)	25·8 (23·3 to 28·6)	−6·7% (−8·0 to −5·6)	5040 (3590 to 6710)	62·4% (55·2 to 70·1)	65·4 (46·6 to 87·0)	−3·8% (−7·4 to −0·2)
Central Europe	1980 (1770 to 2220)	3·1% (−0·5 to 6·9)	13·7 (12·3 to 15·2)	−9·0% (−9·8 to −8·1)	12 200 (8740 to 16 500)	37·9% (30·3 to 44·8)	57·1 (41·3 to 76·5)	−6·2% (−8·5 to −3·6)
Central Latin America	9110 (8200 to 10100)	91·3% (83·4 to 99·5)	35·7 (32·1 to 39·6)	−9·6% (−10·4 to −8·9)	13 700 (9880 to 18 200)	199·0% (188·0 to 212·0)	54·8 (39·7 to 73·0)	−1·8% (−4·3 to 1·0)
Central sub-Saharan Africa	3840 (3430 to 4330)	115·0% (110·0 to 120·0)	38·3 (34·4 to 42·5)	−10·2% (−12·0 to −8·3)	4710 (3400 to 6310)	131·0% (117·0 to 148·0)	82·8 (60·3 to 109)	−5·9% (−11·3 to 0·0)
East Asia	60 100 (53 300 to 67 200)	53·4% (44·8 to 62·2)	34·1 (30·6 to 37·8)	−10·3% (−11·1 to −9·6)	164 000 (117 000 to 216 000)	146·0% (137·0 to 155·0)	73·8 (53·2 to 97·0)	−2·2% (−4·8 to 0·7)
Eastern Europe	5340 (4770 to 5960)	2·5% (0·1 to 4·9)	20·4 (18·4 to 22·7)	−5·5% (−6·2 to −4·8)	26 000 (18 800 to 34 400)	37·5% (33·4 to 41·5)	75·2 (54·7 to 98·8)	9·1% (6·3 to 12·2)
Eastern sub-Saharan Africa	11 300 (10 100 to 12 800)	94·0% (91·6 to 96·8)	34·9 (31·6 to 38·6)	−14·1% (−14·9 to −13·2)	17 300 (12 900 to 21 800)	114·0% (107·0 to 121·0)	95·2 (71·8 to 120)	−7·9% (−10·7 to −4·9)
High-income Asia Pacific	9840 (8670 to 11000)	58·8% (50·3 to 68·6)	33·4 (30·3 to 37·1)	−5·5% (−6·1 to −4·7)	1980 (1330 to 2810)	121·0% (93·0 to 147·0)	4·57 (3·07 to 6·43)	0·2% (−3·7 to 4·5)
High-income North America	5140 (4580 to 5700)	46·0% (42·3 to 49·7)	11·3 (10·2 to 12·6)	−2·3% (−2·9 to −1·6)	4930 (3480 to 6680)	77·3% (71·9 to 82·6)	7·94 (5·67 to 10·8)	−0·3% (−2·7 to 1·9)
North Africa and Middle East	14 400 (13 100 to 16 000)	80·5% (74·3 to 87·5)	26·8 (24·2 to 29·7)	−10·1% (−10·9 to −9·2)	14 500 (10 300 to 19 500)	136·0% (127·0 to 143·0)	32·1 (22·9 to 43·5)	−14·0% (−16·8 to −11·5)
Oceania	376 (338 to 421)	99·2% (94·6 to 104·0)	36·5 (32·9 to 40·5)	−6·6% (−8·3 to −4·5)	403 (284 to 544)	125·0% (109·0 to 145·0)	59·1 (42·1 to 79·5)	−4·3% (−10·5 to 3·6)
South Asia	60 100 (54 100 to 66 900)	73·9% (67·3 to 80·8)	38·3 (34·5 to 42·6)	−12·1% (−12·8 to −11·4)	167 000 (122 000 to 218 000)	136·0% (129·0 to 144·0)	110 (80·9 to 143)	−4·0% (−6·2 to −1·4)
Southeast Asia	30 500 (27 600 to 33 800)	49·5% (43·7 to 55·8)	48·0 (43·5 to 53·0)	−11·9% (−13·0 to −10·9)	29 500 (21 100 to 39 800)	129·0% (120·0 to 137·0)	47·5 (34·0 to 63·6)	−9·6% (−12·3 to −6·7)
Southern Latin America	1290 (1160 to 1440)	44·8% (39·7 to 49·9)	17·5 (15·8 to 19·5)	−6·2% (−8·0 to −4·1)	489 (332 to 696)	75·6% (62·2 to 90·9)	5·78 (3·92 to 8·21)	−5·8% (−13·8 to 2·1)
Southern sub-Saharan Africa	2530 (2290 to 2810)	53·7% (49·3 to 58·1)	35·8 (32·3 to 39·6)	−9·1% (−10·0 to −8·2)	9570 (7130 to 12 100)	126·0% (118·0 to 136·0)	150 (113 to 190)	3·2% (−0·6 to 7·5)
Tropical Latin America	8490 (7620 to 9460)	77·4% (69·7 to 85·2)	36·4 (32·8 to 40·3)	−10·0% (−10·8 to −9·2)	8840 (6250 to 12 000)	162·0% (148·0 to 176·0)	35·8 (25·3 to 48·7)	−7·8% (−10·7 to −5·1)
Western Europe	10 900 (9640 to 12 200)	32·7% (28·8 to 37·0)	17·3 (15·6 to 19·1)	−5·1% (−5·9 to −4·4)	4490 (3010 to 6340)	50·6% (43·7 to 59·5)	5·08 (3·41 to 7·21)	−3·3% (−6·1 to 0·3)
Western sub-Saharan Africa	16 000 (14 400 to 17 900)	124·0% (122·0 to 127·0)	44·0 (39·8 to 48·7)	−7·1% (−7·5 to −6·5)	20 000 (14 600 to 26 000)	148·0% (140·0 to 155·0)	92·6 (68·1 to 120)	4·9% (2·7 to 6·9)

Data in parentheses are 95% uncertainty intervals. Count data are presented to three significant figures, and percentage changes to one decimal place. Crude prevalence rates are in the appendix (p 106).

We estimated 510 million (95% UI 371–667) people were affected by vision impairment from uncorrected presbyopia in 2020 (crude prevalence 64·6 [47·1–84·5] cases per 1000), of whom 280 million (55%; 205–365) were female (table 2; appendix p 106). Among those aged 50 years and older, 221 (155–296) individuals per 1000 population had vision impairment from uncorrected presbyopia (appendix p 107). As with blindness and moderate and severe vision impairment, most of those with mild vision impairment and vision impairment from uncorrected presbyopia resided in south Asia, followed by east Asia and southeast Asia (table 2).

In 2020, age-standardised prevalence for all severities of vision impairment was higher in females aged 50 years and older than in males of the same age group (difference between means and 95% UIs for blindness: 0·00212 [0·00167–0·00265]; moderate and severe vision impairment: 0·0121 [0·0106–0·0137]; mild: 0·0114 [0·00969–0·0130]; and due to presbyopia: 0·0215 [0·0148–0·0286]). This between-sex difference was largest for vision impairment from presbyopia, and smallest for blindness.

**Figure 1:**
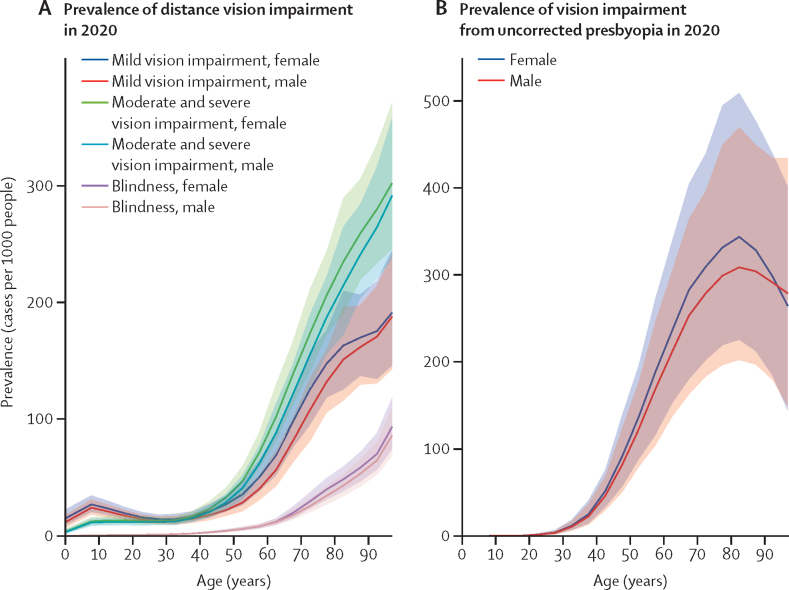
Estimated age-specific prevalence of distance vision impairment (A) and vision impairment from uncorrected presbyopia (B), by sex, in 2020 Solid lines show sex-specific prevalence estimates, with shaded areas indicating 95% uncertainty intervals.

We estimated that in 2020 the population aged 50 years and older (24·1% of the global population) accounted for 33·6 million (78%; 95% UI 28·6–38·5) of 43·3 million people who were blind, 206 million (70%; 182–233) of 295 million people with moderate and severe vision impairment, 143 million (55%; 122–163) of 258 million people with mild vision impairment, and 419 million (82·2%; 295–562) of 510 million people with vision impairment from uncorrected presbyopia (appendix p 107). The strong association between age and vision impairment is presented for various severities of vision impairment in figure 1. For example, in terms of global blindness, 1·67 (1·36–2·01) per 1000 people aged 30–34 years, 6·24 (4·78–7·90) per 1000 people aged 50–54 years, 27·8 (22·2–34·7) per 1000 people aged 70–74 years, and 68·6 (55·5–85·9) per 1000 people aged 90–94 years, were blind (appendix p 107). Crude prevalence of vision impairment categories by age group are given in the appendix (p 107).

To compare patterns and trends in the prevalence of vision impairment without being confounded by the age structure, we calculated age-standardised prevalence, focusing on older adults (aged ≥50 years), who had the largest burden of vision impairment (appendix p 108). In 2020, the age-standardised prevalence of blindness in this age group was 18·5 (95% UI 15·7–21·1) per 1000 people. Regions where prevalence of blindness exceeded 30 cases per 1000 people included southern (30·9 per 1000), eastern (40·0 per 1000), and western (42·2 per 1000) sub-Saharan Africa; south Asia (35·3 per 1000); and southeast Asia (37·2 per 1000). By contrast, the prevalence of blindness was less than 5 cases per 1000 people in the high-income North America (3·98 per 1000), high-income Asia Pacific (4·24 per 1000), and Australasia (4·87 per 1000).

Global age-standardised prevalence of moderate and severe vision impairment was 112 (95% UI 99·0–126) cases per 1000 people in this older age group (appendix p 108). The region with the highest prevalence of moderate and severe vision impairment was south Asia (229 cases per 1000 people) with a considerably higher prevalence than the next two regions of highest prevalence, Oceania (173 per 1000) and southeast Asia (154 per 1000). Mild vision impairment affected 77·2 (66·2–88·2) people per 1000 of the global population in this older age group, with highest prevalence noted in southeast Asia (117 per 1000) and south Asia (108 per 1000; appendix p 108).

**Figure 2:**
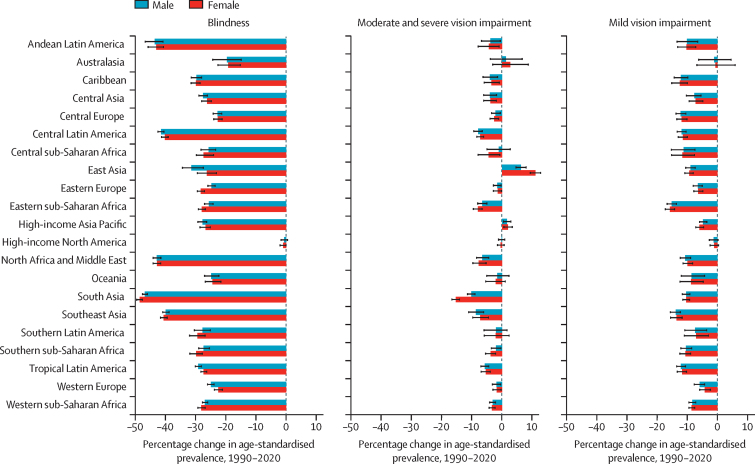
Estimated percentage change in age-standardised prevalence of blindness, moderate and severe vision impairment, and mild vision impairment among individuals aged 50 years and older, by region and sex, 1990–2020 Bars show the percentage change in age-standardised prevalence, with errors bars indicating 95% uncertainty intervals.

Change in age-standardised prevalence of mild vision impairment, moderate and severe vision impairment, and blindness in adults aged 50 years and older between 1990 and 2020 is shown in figure 2, globally and by region and sex; numbers affected, and change in age-standardised prevalence are shown in the appendix (p 108), as are numbers affected and crude prevalence of vision impairment categories in 1990 for all ages (appendix p 109).

Globally between 1990 and 2020, among older adults, we estimated that age-standardised prevalence of blindness decreased by 28·5% (95% UI −29·4 to −27·7; from 25·8 [22·2 to 29·6] cases per 1000 in 1990, to 18·5 [15·7 to 21·1] cases per 1000 in 2020), with the decrease being larger among males (–33·2%, −34·0 to −32·3) than among females (–25·0%, −26·0 to −23·9). Change in crude prevalence for those aged 50 years and older for this period was −21·9% (–23·3 to −20·6) in females and −27·4% (–28·7 to −26·2) in males; and for all ages was 4·6% (2·6 to 6·6) for females and −0·7 (–2·6 to 1·1) for males. This reduction among both sexes was noted in all regions, but in particular in south Asia, where a change of −47·6% (–48·4 to −46·9) was observed for both sexes combined in those aged 50 years and older. In this time period, for moderate and severe vision impairment, global estimated age-standardised prevalence among those aged 50 years and older increased slightly by 2·5% (1·9 to 3·2; from 109 [96·7 to 123] cases per 1000 in 1990 to 112 [99·0 to 126] cases per 1000 in 2020), and a more substantial increase was seen among females (4·27%, 1·86 to 3·17) than among males (0·35%, −0·31 to 1·03). We found considerable inter-region variation in this change—eg, for both sexes combined, south Asia (–12·6%, −13·8 to −11·6) and southeast Asia (–7·7%, −10·0 to −5·2) had the most substantial decreases, whereas an increase of 8·5% (7·1 to 9·9) was found for east Asia. For mild vision impairment, age-standardised prevalence reduced slightly by 0·3% (–0·8 to −0·2; from 77·8 [66·3–88·4] cases per 1000 in 1990 to 77·3 [66·2–88·2] cases per 1000 in 2020) globally and in all regions, with a reduction of 3·1% (–3·7 to 2·4) in males whereas the prevalence in females increased by 1·9% (1·2 to 2·5). For vision impairment from uncorrected presbyopia, we were not able to do global and inter-regional analyses due to sparsity of data.

**Figure 3:**
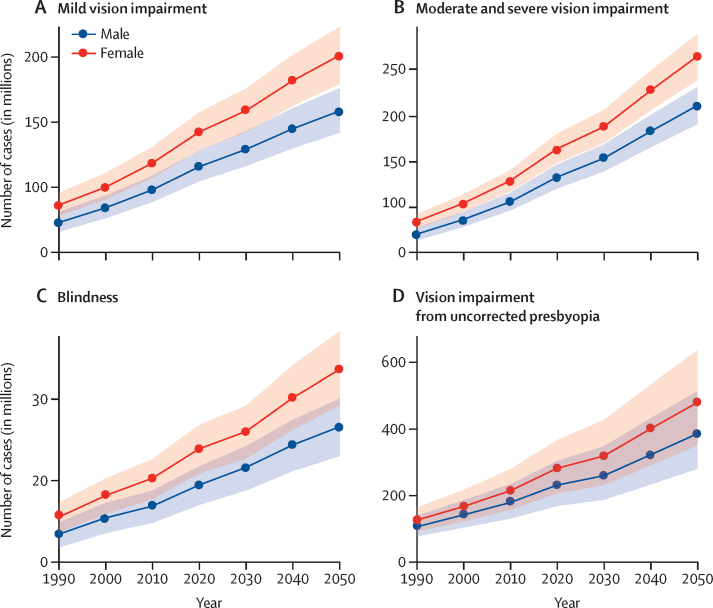
Forecast of number of people affected by mild vision impairment (A), moderate and severe vision impairment (B), blindness (C), and vision impairment from uncorrected presbyopia (D), all ages by sex, 1990–2050 Datapoints are estimates number of people affected in millions, with lines linking the datapoints and shaded areas indicating the 95% uncertainty intervals.

By 2050, the number of people with blindness in the global population is predicted to increase to 61·0 million (95% UI 52·9–69·3). Moderate and severe vision impairment is predicted to affect 474 million (428–518) people, mild vision impairment to affect 360 million (322–400) people, and vision impairment from uncorrected presbyopia to affect 866 million (629–1150) people. A breakdown of estimates by sex is given in figure 3 and by region and sex in the appendix (pp 112–113).

GBD has published YLD data for 2019;^23^ therefore, the report and analyses that follow relate to 2019. Sense organ diseases form an aggregate of disability resulting from blindness and vision loss due to eye diseases (near vision loss, uncorrected refractive error, glaucoma, age-related macular degeneration, cataract, and other vision loss), age-related and other hearing loss, and other sense organ diseases. These diseases resulted in 66·1 million (95% UI 45·1–93·0) YLDs or 7·7% (6·4–9·3) of total YLDs in 2019. Blindness and vision loss resulted in 22·6 million (15·6–31·7) global YLDs in 2019, a 20·3% (18·9–21·9) increase since 2010; YLD data can be viewed through the GBD Compare tool.

When ranked against all causes of disease by YLDs in the Global Burden of Diseases, Injuries and Risk Factors Study 2019, blindness and low vision ranked eighth (contributing 3·8% [95% UI 3·0–4·9] of all YLDs) in the aged 50–69 years age group for both sexes (following, in order of the highest ranking, low back pain, diabetes, age-related hearing loss, other musculoskeletal causes, depressive disorders, osteoarthritis, and headache disorders), and fourth (contributing 6·4% [5·4–7·4] of all YLDs) in the aged 70 years and older age group (following, in order of the highest ranking, age-related hearing loss, diabetes, and low back pain).

## Discussion

In 2013, the World Health Assembly adopted resolution 66.4, which included *Towards universal eye health: a global action plan 2014–2019*.^3^ The vision was “A world in which nobody is needlessly visually impaired, where those with unavoidable vision loss can achieve their full potential and where there is universal access to comprehensive eye care services.”^3^ The primary indicator of the global action plan was the prevalence and causes of vision impairment, which included moderate and severe vision impairment and blindness. The global action plan also set a specific global target: a reduction in the prevalence of avoidable vision impairment of 25% by 2019 from the baseline of 2010. Has this target been met? In 2010, of the global population of 6·9 billion people, we estimate 37·0 million people were blind and an additional 233·5 million people had moderate and severe vision impairment. Hence, the prevalence of vision impairment, as defined by the global action plan, was 3·92%. In the current study, we estimate that there are 43·3 million people who are blind and an additional 295 million people with moderate and severe vision impairment in a total world population of 7·79 billion in 2020. Hence, the prevalence of vision impairment in 2020, as defined by the global action plan, was 4·34%. Although in this study we are not specifically addressing so-called avoidable vision impairment that the global action plan specified in its global target (the causal breakdown into avoidable and unavoidable vision impairment is covered in another report by our group^27^), in terms of crude prevalence, the target has clearly not been met. The increase in prevalence is determined by at least two variables: the need and the provision of services. Factors contributing to the extent of provision of services include the extent of services available (dependent on the availability of limited resources allocated to blindness alleviation *vs* other competing priorities); awareness of the population that the services are available; perceived need for services among the population (including assurance of quality); and the extent to which barriers such as cost of travel, fees, and fears can be overcome. Sustainability of the efforts put into provision of these services is one of many long-term needs. Although the observed decrease in age-adjusted burden of vision impairment suggests systematic improvements are occurring, more improvements are needed to alleviate the problem of vision impairment to the extent envisioned in the global action plan. These measures would need to address both the supply of services and factors affecting demand for the services.

However, encouragingly, the age-adjusted prevalence of blindness was estimated to have reduced by 27·0% (95% UI −27·8 to −26·1) globally between 1990 to 2020. Conversely, the age-adjusted prevalence of moderate and severe vision impairment increased slightly (1·1%, 0·6 to 1·6). This slight increase is probably due to resources for cataract being directed to more severe disease (cataract surgical coverage is substantially higher for eyes with visual acuity of less than 3/60 than for eyes with a visual acuity of less than 6/18, especially in the most recent RAAB surveys and in low-income and middle-income countries,^5^ although the causal contribution to the change in age-standardised prevalence has been investigated in another report by our group^27^).

As we noted in the 2015 estimates,^4^ approximately 75% of the global population who are blind are aged 50 years and older. Although the growth rate of the world's population has been decreasing since the late 1960s, the world's population has continued to increase, predicted to reach an inflection point in 2020. The current total population of 7·79 billion is expected to plateau at approximately 11 billion by 2100.^28^ The rate of population growth remains especially high in the group of 47 countries designated by the UN as the least developed, including 32 countries in sub-Saharan Africa.^28^ In 2018, for the first time in human history, people aged 65 years or older outnumbered children younger than 5 years.^29^ Between 2020 and 2050, the proportion of the world's population aged 65 years or older is expected to double, from approximately 1 billion to 2 billion.^28^ The ageing of the world's population will have critical ramifications on age-related diseases, including age-related blindness from cataract, uncorrected refractive error, age-related macular degeneration, and glaucoma.

Furthermore, the increasing proportion of the world's population that are now in the oldest age groups deserves specific attention. In this study, we found that the prevalence of blindness in those aged 90–94 years was more than 11 times greater than in those aged 50–54 years. However, we should be mindful that although the regions undergoing development with increasing life expectancies face the challenge of age-related vision impairment, the most under-developed and economically disadvantaged regions, particularly sub-Saharan Africa, still have the highest prevalence of blindness in older adults.

Another important finding made possible with the more granular data we used in this analysis than in previous analyses, was the sex difference in the vision impairment burden. The VLEG and others have previously noted a higher prevalence of vision impairment in women than in men, particularly in low-income and middle-income countries.^4^, ^29^ The factors contributing to this disparity are complex and can be attributed to biological (eg, women tending to live longer than men and are more at risk of developing some conditions such as cataract) and social factors (eg, social discrimination that appears to result in more women having vision loss than men when no such a magnitude of difference in incidence of disease by sex is clearly seen). In the current study, we observed that, between 1990 and 2020, the age-standardised prevalence of blindness in older adults reduced by 25·0% in women and 33·2% in men. However, for mild vision impairment and moderate and severe vision impairment, the global age-standardised prevalence for women increased. These data provide evidence-based motivation to understand the region-specific reasons for the sex discrepancy and initiate strategies to close this gap.

The age-standardised prevalence of moderate and severe vision impairment in east Asia increased between 1990 and 2020 by 10·7%. This increase is most likely related to the myopia epidemic that began approximately 30 years ago in this region.^30^ It might also result from a reduction in the prevalence of blindness, with people who were formerly blind moving to the category of moderate and severe vision impairment because of residual or coexisting ocular disease or damage. An increase in progressive axial myopia has paralleled the increase in education levels during the past 30 years in parts of east Asia.^30^ In a similar way that trachoma was once described as a so-called disease of the crèche (preschool child care), converging evidence has indicated that myopia is largely a so-called disease of the classroom, especially the competitive classroom.^30^ Genetic factors have a relatively minor role.^31^ The recognition that myopia is driven largely by environmental factors related to intensive schoolwork has motivated successful treatment strategies that increase time spent outdoors for schoolchildren.^32^ These simple and inexpensive strategies will need to be widely adopted, particularly as development and associated improvements in education levels increase over the coming decades.

Presbyopia was reported to affect more than 1 billion people globally in 2005; however, this estimate was based on robust data from only four countries (India, Tanzania, Brazil, and Timor-Leste).^33^ In response to the paucity of data, WHO developed a standardised protocol for assessing the prevalence of vision impairment from uncorrected presbyopia.^34^, ^35^ A 2018 meta-analysis estimated that, in 2015, 826 million people had near vision impairment due to no, or inadequate, presbyopic correction.^36^ The relative sparsity of data about uncorrected presbyopia remains problematic. Until 2019, the International Classification of Diseases did not have a definition of near vision impairment. This limitation prevents the presentation of meaningful analysis of the temporal change in prevalence of vision impairment from uncorrected presbyopia. However, it exemplifies the importance of surveys incorporating a near vision component to better understand future trends.

This study highlights the large burden of mild, moderate, and severe vision impairment that could become overwhelming as the global population ages in both high-income countries and low-income and middle-income-countries. While low-income and middle-income countries are challenged with detecting and delivering care to those already in an advanced state of vision loss, high-income countries face service delivery challenges to manage chronic age-related eye diseases in an ageing population with increasing life expectancy.^37^

The VLEG contributed data on vision impairment prevalence to WHO's recent *World report on vision,* based on the VLEG's 2015 estimates.^5^ WHO recognises the challenges being faced and recommends that eye care become an integral part of universal health coverage.^5^ Such integration would be beneficial, but impactful reduction in the prevalence of vision loss will also require concerted action targeting both avoidable blindness due to cataract and uncorrected refractive error and strategies to restrict vision impairment from chronic age-related diseases and diabetic eye disease.

The strengths of this updated review and analysis for 2020 include the substantial addition of new data sources and the wider geographical region that these sources cover. In particular, the contribution of the RAAB surveys, most of which took place in low-income and middle-income countries, has been enhanced by the extraction of data disaggregated into smaller age categories, which has greatly strengthened the fitting of age patterns to the data from these surveys. However, our study also had several limitations. For example, several regions, such as central sub-Saharan Africa, central and eastern Europe, and central Asia, still have little or no population-based data, so estimates in these regions rely on extrapolation from other regions. Sparse data also restricted our ability to estimate temporal trends. Few data were available for children, and although we were able to include data from the Refractive Errors Studies in Children, which involved robust population-based sampling,^38^ many studies containing data on children were done in school settings. Such reporting might lead to bias in countries with low school enrolment. Additionally, approximately 15–20% of included sources reported best-corrected vision instead of presenting vision, and therefore we adjusted these data to the reference definition. These adjustments were based on data that matched presenting and best-corrected data. Because the uncertainty of this adjustment is carried forwards, these datapoints have wide uncertainty intervals, but were weighted accordingly in the Bayesian modelling. We describe inclusion criteria used when assessing potential sources of vision data in the appendix. During data processing, we applied data adjustments to studies that did not match our reference definition to make use of as much data as possible. For example, our reference definition is presenting visual acuity, so we adjusted best-corrected visual acuity systematically on the basis of the results of a regression analysis comparing the prevalence of the two methods. Our estimation tool (DisMod-MR) quantifies between-study heterogeneity (the part of variance not ascribed to fixed effects or geographical random effects) and adds uncertainty based on that finding. Notably, this process does not preclude any remaining measurement error that we were unable to account for.

For the first time in our reports, we have incorporated YLDs into our analysis. Although VLEG remains concerned over the disproportionally low disability weights assigned to blindness and vision impairment,^39^, ^40^ these disabilities are responsible for 2·6% (95% UI 2·2–3·2) of total global YLDs, and are highly ranked against other age-related diseases.^24^ Younger people with blindness (and moderate and severe vision impairment) are more likely to have an irreversible cause of vision impairment than older people. This fact and the longer remaining life expectancy mean that blindness and vision impairment at a younger age are potentially more important than at an older age in terms of YLDs.

The WHO *World report on vision* observes that “Concerted action during the past 30 years has yielded many successes: global advocacy efforts have been launched; World Health Assembly resolutions adopted; and actions plans implemented. Recent scientific and technological developments promise to further accelerate these advances. Nonetheless, progress is not keeping pace with population eye care needs.”^5^ We are encouraged by the reduction in the global prevalence of age-related blindness but the increase in the crude prevalence of blindness is sobering. This evidence indicates that we face enormous challenges as the global population grows and ages. Strategic and cost-effective approaches targeting cataract and uncorrected refractive error need renewed impetus and clever use of emerging technologies towards solving the problem of global vision impairment should be a health priority. Because most cases of vision impairment require individual-level clinical care, training and sustainable delivery system development require renewed attention.

Correspondence to: Prof Rupert RA Bourne, Vision and Eye Research Institute, Anglia Ruskin University, Cambridge CB1 1PT, UK rb@rupertbourne.co.uk

## Data sharing

To download the data used in these analyses, please visit the Global Health Data Exchange GBD 2019 website.
