# Supplementary material for: Trends in prevalence of blindness and distance and near vision impairment over 30 years: an analysis for the Global Burden of Disease Study
Source: Lancet Glob Health. 2020 Dec 1;9(2):e130–43. doi: 10.1016/S2214-109X(20)30425-3 (PMC7820390; doi:10.1016/S2214-109X(20)30425-3)
Supplement: Supplementary appendix [file mmc1.pdf]

THE LANCET
Global Health

Supplementary appendix

This appendix formed part of the original submission and has been peer reviewed. We post it as supplied by the authors.

Supplement to: GBD 2019 Blindness and Vision Impairment Collaborators on behalf of the Vision Loss Expert Group of the Global Burden of Disease Study. Trends in prevalence of blindness and distance and near vision impairment over 30 years: an analysis for the Global Burden of Disease Study. *Lancet Glob Health* 2020; published online Dec 1. http://dx.doi.org/10.1016/S2214-109X(20)30425-3.

APPENDIX 1: SUPPLEMENTARY METHODOLOGY

Contents

Text 1. Definitions and analytic framework

Text 2. Data identification and access

Text 2.1. Developing the Search Strategy (distance visual acuity data sources)

Text 2.2. Final Search Strategies (distance visual acuity data sources)

Text 2.3. Results and Discussion (distance visual acuity data sources)

Text 2.4. Systematic review of studies of vision impairment from uncorrected presbyopia

Text 3. Methods for comparability of vision impairment definition

Text 4. Methods for comparability of data aggregated by age

Text 5. Methods for calculating prevalence of vision impairment by country, year, age, and sex

Text 6. Calculation of Years Lived with Disability (YLDs)

Text 7. GATHER compliance and access to code used in the analytical model

Table 1. Countries and territories in analysis regions.

Table 2. Characteristics of data sources used in the analysis that reported distance vision impairment.

Table 3. Characteristics of data sources used in the analysis that reported vision impairment from uncorrected presbyopia

Table 4. PRISMA-P checklist

Table 5. Sources searched for the 2018 update

Table 6: Literature search results

Table 7. Adjustment factors for non-reference definition data.

Table 8. Fixed effects for sex (study-level covariate), healthcare access and quality index (country-level covariate), and/or sociodemographic index that were included in Dismod models.

Table 9. Health state descriptions and accompanying disability weight.

Figure 1. PRISMA flowchart

Figure 2. Meta-regression results to split mixed severity data into single severity data points

APPENDIX 2: SUPPLEMENTARY RESULTS

Contents

Table 1A. Crude prevalence of moderate and severe vision impairment (MSVI) and blindness in 2020, globally, by region and by sex

Table 1B. Crude prevalence of mild vision impairment and vision impairment from uncorrected presbyopia in 2020, globally, by region and by sex

Table 2. Crude prevalence of blindness, moderate and severe vision impairment, mild vision impairment and vision impairment from uncorrected presbyopia in 2020, globally, by age group

Table 3A. Number of persons affected and age-standardised prevalence of moderate and severe vision impairment (MSVI) and blindness in 2020 and change since 1990 in adults aged 50 years and older.

Table 3B. Number of persons affected and age-standardised prevalence of mild vision impairment and vision impairment from uncorrected presbyopia in 2020 and change since 1990 in adults aged 50 years and older.

Table 4. Number of persons and crude prevalence of persons blind, with MSVI, with mild VI, and vision impairment from uncorrected presbyopia in 1990

Table 5A. Forecasted number of persons and age-standardized prevalence of moderate and severe vision impairment and blindness in 2050 by region

Table 5B. Forecasted number of persons and age-standardized prevalence of mild vision impairment and vision impairment from uncorrected presbyopia in 2050 by region

Figure 1: Data Sources used in the analysis from RAAB (Map A) and non-RAAB (Map B) studies.

Figure 2A-D. Forecast of numbers of people affected by blindness, moderate and severe vision impairment, mild vision impairment and vision impairment from uncorrected presbyopia to 2050- by region.

APPENDIX 3: CONTRIBUTORS

APPENDIX 1: METHODOLOGY

Text 1. Definitions and analytic framework

We estimate the prevalence of vision loss for the period 1990-2020 by age, sex, and year in 187 countries (Table 1), for the following extended vision loss categories:

•Blind: < 3/60 and/or a visual field of no greater than 10° in radius around central fixation

•Severe vision loss: < 6/60 and ≥ 3/60
•Moderate vision loss: < 6/18 and ≥ 6/60
•Mild vision loss: < 6/12 and ≥ 6/18

•Vision impairment from uncorrected presbyopia: VA of <6/12 distance equivalent in those with presenting distance vision ≥6/12

Our study was carried out in seven steps:

•Data identification and access

•Separation of raw data into datasets called "vision loss envelopes" for all-cause mild, moderate, and severe vision loss, and blindness.
•Studies only specifying "both" sex information split into male- and female-specific data points using MR-BRT*

•Adjustment of non-reference data to the reference definition of presenting vision data that fit within the WHO severity categories

• Application of an age pattern to data with age groups greater than or equal to 25 years

• Dismod-MR 2.1 modeling

• Modeling and Post-Processing
• Extrapolation, Age-standardisation & forecasting for 2020 and 2050 estimates

*MR-BRT (Meta Regression; Bayesian; Regularized; Trimmed)- a mixed-effects meta-regression tool developed at the Institute for Health Metrics and Evaluation This tool was developed in part to allow for the ability to propagate between-study heterogeneity as part of the uncertainty adjustment, and to allow trimming of outlier input data. A detailed description of MR-BRT has been published elsewhere.[1 2]

Text 2. Data identification and access

We used data from epidemiologic studies and surveys which we collected to update a previously published systematic review. Following Bourne and colleagues,[3] we identified studies or surveys fulfilling the following inclusion criteria:

- The studies reported prevalence of vision impairment from cross-sectional surveys of representative populations of a country or area of a country.
- The definitions of vision impairment were clearly stated, using thresholds of visual acuity in the better eye that matched the extended definitions of vision impairment.
- Best corrected and/or presenting visual acuity was given.
- The procedures used for measurement of visual acuity were clearly stated.

The authors carried out a systematic review of the literature for distance vision impairment data. They also obtained unpublished data sources. Distance visual acuity data was used from 512 sources; 485 reported or could be used to estimate the prevalence of blindness, 380 reported or could be used to estimate the prevalence of moderate plus severe vision impairment (MSVI), and 59 reported or could be used to estimate the prevalence of mild vision impairment. Data sources used in this analysis are listed in Table 2. 25 studies were used to estimate vision impairment from uncorrected presbyopia, as shown in Table 3. The current systematic review of distance visual acuity data sources used the same search terms of a systematic review published previously,[4] but extended the review to include more recently published studies up to 1 October 2018. The methodology for this systematic review extension is described in Figure 1 as a PRISMA (Preferred Reporting Items for Systematic Reviews and Meta-Analyses) flowchart with a PRISMA-P checklist in Table 4.

The Search Strategy for the Systematic Review is described in the following sections:

2.1 Developing the Search Strategy (distance visual acuity data sources)
2.2 Final Search Strategies (distance visual acuity data sources)
2.3 Results & Discussion (distance visual acuity data sources)

2.4 Systematic review of studies of presbyopia prevalence

Text 2.1. Developing the Search Strategy (distance visual acuity data sources)

The search strategy was required to capture epidemiological studies of vision loss and blindness published in the period 1980 to 2018 inclusive. The search excluded animal studies.

This report describes update searches undertaken for the Global Vision Database project in October 2018. These searches update the original search undertaken in 2008 and the subsequent searches run in 2012 and 2013 and 2014.

Several approaches to capturing the search concepts were developed and tested out in Ovid MEDLINE. The objective was to achieve a focused strategy that would identify epidemiological studies of blindness. The strategy needed to balance adequate sensitivity (not missing too many relevant records) against reasonable precision (not producing high proportions of irrelevant records) bearing in mind the resource available to process the records produced by the searches. To achieve this balance required the use, in a master strategy for MEDLINE, of the various indexing options that were available. The strategy also had to take account of variability in the way authors describe their research and indexers index research with Medical Subject Headings (MeSH). Ideally, we would expect all records about the epidemiology of blindness to be indexed with the MeSH and appropriate subheading, for example BLINDNESS/ep. In practice, however, there are many records about that topic that are not indexed in that way. The strategy takes account of such variability in indexing approaches. The strategy also had to cope with the fact that the search would be undertaken in both indexed and unindexed records (e.g. MEDLINE In Process records) so had to search for text words (in the title and abstract) as well as MeSH.

As well as the concept of the epidemiology of blindness, the strategy had to retrieve studies about the epidemiology of selected specific eye diseases which might result in blindness or visual impairment.

After several iterations, checking the impact of changes at each stage, a strategy with three elements was agreed as follows for Ovid MEDLINE (see also Box 1).

Element 1

A search on the concepts of blindness and visual impairment linked to epidemiological terms through three approaches:

1. Precoordination of a MeSH blindness/visual impairment term with its epidemiology subheading, e.g.

   exp blindness/ep

2. Searching for the occurrence of a MeSH blindness/visual impairment term along with the occurrence of an epidemiological MeSH term in the same record, e.g.

   exp blindness/ and (incidence/ or prevalence/ or mortality/ or morbidity/)

3. Searching for the occurrence of a MeSH blindness/visual impairment term and an epidemiological concept ex- pressed as a text word in the title or abstract of a record, e.g.

   exp blindness/ and (epidemiology or incidence or prevalence or mortality).ti,ab.

Element 2

A search for specific eye diseases linked to epidemiological terms and also to terms indicating blindness using the following approaches:

1. Precoordination of a MeSH eye disease heading with the epidemiology subheading and then looking for the occurrence of that combination in the same record as one where blindness/visual impairment is a term in the title or abstract, e.g.

   • conjunctival diseases/ep
   • exp blindness/ or (blindness or (visual adj3 impair$) or (vision adj3 impair$)).ti,ab. • 1 and 2

2. Searching for the occurrence of a MeSH eye disease heading in the same record as an epidemiological MeSH heading and as blindness/visual impairment as a term in the title or abstract, e.g.

   • conjunctival diseases/ and (incidence/ or prevalence/ or age distribution/ or sex distribution/ or morbidity/) • exp blindness/ or (blindness or (visual adj3 impair$) or (vision adj3 impair$)).ti,ab.
   • 1 and 2

3. Searching for the occurrence of eye disease text words in close proximity to epidemiology text words in the title and abstract of records, in the same records as blindness MeSH or blindness/visual impairment text words, e.g.

   • (retinitis adj3 (incidence or prevalence or epidemiology or mortality)).ti,ab.
   • exp blindness/ or (blindness or (visual adj3 impair$) or (vision adj3 impair$)).ti,ab. • 1 and 2

Element 3

Searching for records that specifically mention population-based eye surveys using a range of synonyms for that concept in the title and abstract, e.g.

(population adj3 eye adj3 survey$).ti,ab.

To improve the precision of the search a range of limits were explored and agreed

- Removing studies about animals by using a safe exclusion approach: {Result set} NOT (animals/ NOT humans/)
- Excluding letters coded in the MEDLINE Publication Type field
- Excluding comments coded in the MEDLINE Publication Type field
- Excluding editorials coded in the MEDLINE Publication Type field
- Excluding clinical trials coded in the MEDLINE Publication Type field
- Excluding reviews coded in the MEDLINE Publication Type field or as a text word in the title

The original 2008 search was limited to records with a publication date of 1980 to current. The update searches were date limited using various update codes relevant to the specific databases. These date limits were intended to restrict the results to records added to the databases, indexed or otherwise revised since the search was last run. The results of each update were deduplicated against each other and the results of the previous searches. The following databases were searched in 2008 and for each of the subsequent updates:

- Ovid MEDLINE(R) In-Process & Other Non-Indexed Citations and Ovid MEDLINE(R)
- Ovid EMBASE
- WHOLIS library catalogue (http://dosei.who.int/uhtbin/webcat)

For the 2018 update, the search structure and terms used were identical to those used previously. No further revisions of the strategy were made. The original strategies were translated appropriately for the sources newly searched for this update e.g. SciELO, Open Grey, and the new interface of WHOLIS. Full search strategies for all resources are provided below in Text 2.2.

The original 2008 search downloaded records from MEDLINE and Embase by country. This was not repeated in the 2012, 2013 or 2014 update searches. We followed the approach used in previous update searches and downloaded the results as one batch, without an additional search concept to allow records to be exported geographically.

The previous update searches (2012, 2013, 2014) were date limited using various update codes relevant to the specific databases. These date limits were intended to restrict the results to records added to the databases, indexed or otherwise revised since the search was last run. Whilst this approach of date limiting updates is pragmatic, and reduces the resource burden of loading and deduplicating the large number of records a search unrestricted by date would require, there is currently no agreed best method to achieve this. Due to uncertainty about the way date fields are implemented in database interfaces, many older records are inevitably retrieved and must be removed at deduplication stage. As a result the 2018 update searches were not limited by date. We re-ran all the searches from 1980 to 1 October 2018 and removed records already identified by previous searches.

Text 2.2. Final Search Strategies (distance visual acuity data sources)

The databases searched mirrored those used in the original search with the addition of SciELO and sources to identify grey literature. Table 5 presents the sources searched. The choice of grey literature sources was discussed and agreed with Rupert Bourne and colleagues from The Vision Loss Expert Group before the searches were run.

The following web pages of highly relevant organisations were also searched/browsed for relevant evidence:

- World Health Organization;
- International Agency for the Prevention of Blindness;
- International Council of Ophthalmology;
- International Eye Foundation;
- Commonwealth Eye Health Consortium;
- International Centre for Eye Health (ICEH) at LSHTM;
- Brien Holden Vision Institute;
- Sightsavers.

The final search strategies are shown below.

Key to Ovid MEDLINE search syntax

Exp	Explodes a MeSH to capture more specific MeSH
.ti,ab.	Searches for a word in the title and abstract of a record
/	Indicates that the search term is a Medical Subject Heading (MeSH)
/ep	Searches for a subheading linked to a MeSH, in this case epidemiology
And	Achieves a Boolean AND combination
Or	Achieves a Boolean OR combination
Adj3	Adjacency operator, searches for words up to 3 words apart
$	Truncation operator, searches for words beginning with the stem, e.g. impair$ retrieves impair, impairment, impairments, impaired, impairing and impairs

2.2.1 Medline search

Source: Ovid MEDLINE(R) and Epub Ahead of Print, In-Process & Other Non-Indexed Citations and Daily <1946 to October 01, 2018>
Interface / URL: Ovid
Database coverage dates: 1946 to October 01, 2018
Search date: 02/10/18
Retrieved records: 14219
Search strategy:

Database: Ovid MEDLINE(R) and Epub Ahead of Print, In-Process & Other Non-Indexed Citations and Daily <1946 to October 01, 2018>
Search Strategy:
----
1 exp blindness/ep or exp blindness/mo (2367)
2 exp blindness/ and (incidence/ or prevalence/ or mortality/ or morbidity/) (1464)
3 exp blindness/ and (age distribution/ or sex distribution/) (389)
4 exp blindness/ and exp eye diseases/ep (2775)
5 exp blindness/ and (epidemiology or incidence or prevalence or mortality).ti,ab. (2056)

6 (blindness and (epidemiology or incidence or prevalence or mortality)).ti,ab. (4060)
7 vision/ep or vision/mo (0)
8 vision/ and (age distribution/ or sex distribution/) (13)
9 (vision and (epidemiology or incidence or prevalence or mortality)).ti,ab. (6951)
10 vision/ and (incidence/ or prevalence/ or mortality/ or morbidity/) (101)
11 exp visual acuity/ and (incidence/ or prevalence/ or mortality/ or morbidity/) (3113)
12 exp visual acuity/ and (age distribution/ or sex distribution/) (753)
13 (visual acuity and (epidemiology or incidence or prevalence or mortality)).ti,ab. (5640)
14 vision, binocular/ and (incidence/ or prevalence/ or morbidity/ or mortality/) (89)
15 vision, binocular/ and (age distribution/ or sex distribution/) (25)
16 ((vision adj3 binocular) and (epidemiology or incidence or prevalence or mortality)).ti,ab. (151)
17 Vision, Low/ep or vision, low/mo (613)
18 vision, low/ and (incidence/ or prevalence/ or mortality/ or morbidity/) (485)
19 vision, low/ and (age distribution/ or sex distribution/) (195)
20 (low vision and (epidemiology or incidence or prevalence or mortality)).ti,ab. (463)
21 Night Blindness/ep or night blindness/mo (102)
22 night blindness/ and (incidence/ or prevalence/ or morbidity/ or mortality/) (64)
23 night blindness/ and (age distribution/ or sex distribution/) (7)
24 Presbyopia/ep or presbyopia/mo (82)
25 presbyopia/ and (incidence/ or prevalence/ or mortality/ or morbidity/) (48)
26 presbyopia/ and (age distribution/ or sex distribution/) (24)
27 (presbyopi$ and (epidemiology or incidence or prevalence or morbidity)).ti,ab. (128)
28 Visually Impaired Persons/ and (incidence/ or prevalence/ or morbidity/ or mortality/) (480)
29 visually impaired persons/ and (age distribution/ or sex distribution/) (160)
30 (visual$ adj3 impair$ adj3 (incidence or prevalence or epidemiolog$ or morbidity)).ti,ab. (542)
31 (vision adj3 impair$ adj3 (incidence or prevalence or epidemiolog$ or morbidity)).ti,ab. (95)
32 ((amaurosis or deaf-blind) adj3 (incidence or prevalence or epidemiolog$ or morbidity)).ti,ab. (10)
33 conjunctival diseases/ep or conjunctival diseases/mo (137)
34 conjunctival diseases/ and (incidence/ or prevalence/ or age distribution/ or sex distribution/ or morbidity/ or mortality/) (118)
35 conjunctivitis/ep, mo (509)
36 conjunctivitis/ and (incidence/ or prevalence/ or age distribution/ or sex distribution/ or morbidity/ or mortality/) (175)
37 ((conjunctivitis or conjunctival) adj3 (incidence or prevalence or epidemiolog$ or mortality)).ti,ab. (272)
38 ophthalmia neonatorum/ep, mo (85)
39 ophthalmia neonatorum/ and (incidence/ or prevalence/ or age distribution/ or sex distribution/ or morbidity/ or mortality/) (21)
40 (ophthalmia neonatorum adj3 (incidence or prevalence or epidemiology or mortality)).ti,ab. (11)
41 trachoma/ep, mo (1085)
42 trachoma/ and (incidence/ or prevalence/ or age distribution/ or sex distribution/ or morbidity/ or mortality/) (475)
43 (trachoma adj3 (incidence or prevalence or epidemiology or mortality)).ti,ab. (418)
44 pterygium/ep, mo (173)
45 pterygium/ and (incidence/ or prevalence/ or age distribution/ or sex distribution/ or morbidity/ or mortality/) (124)
46 (pterygium adj3 (incidence or prevalence or epidemiology or mortality)).ti,ab. (99)
47 xerophthalmia/ep, mo (236)
48 xerophthalmia/ and (incidence/ or prevalence/ or age distribution/ or sex distribution/ or morbidity/ or mortality/) (112)
49 (xerophthalmia adj3 (incidence or prevalence or epidemiology or mortality)).ti,ab. (77)

50 corneal diseases/ep, mo or (corneal diseases/ and (incidence/ or prevalence/ or age distribution/ or sex distribution/ or morbidity/ or mortality/)) (540)
51 (corneal adj3 disease$ adj3 (incidence or prevalence or epidemiology or mortality)).ti,ab. (21)
52 corneal opacity/ep, mo or (corneal opacity/ and (incidence/ or prevalence/ or age distribution/ or sex distribution/ or morbidity/ or mortality/)) (126)
53 (cornea$1 adj3 (opaque$ or opac$) adj3 (incidence or prevalence or epidemiology or mortality)).ti,ab. (33)
54 keratitis/ep, mo or (keratitis/ and (incidence/ or prevalence/ or age distribution/ or sex distribution/ or morbidity/ or mortality/)) (461)
55 (keratitis adj3 (incidence or prevalence or epidemiology or mortality)).ti,ab. (197)
56 corneal ulcer/ep, mo or (corneal ulcer/ and (incidence/ or prevalence/ or age distribution/ or sex distribution/ or morbidity/ or mortality/)) (342)
57 (cornea$ adj3 ulcer$ adj3 (incidence or prevalence or epidemiology or mortality)).ti,ab. (49)
58 keratoconus/ep, mo or (keratoconus/ and (incidence/ or prevalence/ or age distribution/ or sex distribution/ or morbidity/ or mortality/)) (208)
59 (keratoconus adj3 (incidence or prevalence or epidemiology or mortality)).ti,ab. (65)
60 eye diseases, hereditary/ep, mo or (eye diseases, hereditary/ and (incidence/ or prevalence/ or age distribution/ or sex distribution/ or morbidity/ or mortality/)) (67)
61 (hereditary adj3 eye adj3 (incidence or prevalence or epidemiology or mortality)).ti,ab. (1)
62 retinitis pigmentosa/ep, mo or (retinitis pigmentosa/ and (incidence/ or prevalence/ or age distribution/ or sex distribution/ or morbidity/ or mortality/)) (242)
63 (retinitis pigmentosa adj3 (incidence or prevalence or epidemiology or mortality)).ti,ab. (37)
64 exp eye infections/ep, mo or (exp eye infections/ and (incidence/ or prevalence/ or age distribution/ or sex distribution/ or morbidity/ or mortality/)) (4443)
65 (infection$ adj3 eye$1 adj3 (incidence or prevalence or epidemiology or mortality)).ti,ab. (13)
66 lens diseases/ep, mo or (lens diseases/ and (incidence/ or prevalence/ or age distribution/ or sex distribution/ or morbidity/ or mortality/)) (72)
67 (lens adj3 disease$ adj3 (incidence or prevalence or epidemiology or mortality)).ti,ab. (3)
68 exp aphakia/ep, mo or (exp aphakia/ and (incidence/ or prevalence/ or age distribution/ or sex distribution/ or morbidity/ or mortality/)) (101)
69 (aphakia adj3 (incidence or prevalence or epidemiology or mortality)).ti,ab. (14)
70 cataract/ep, mo or (cataract/ and (incidence/ or prevalence/ or age distribution/ or sex distribution/ or morbidity/ or mortality/)) (2782)
71 (cataract$ adj3 (incidence or prevalence or epidemiology or mortality)).ti,ab. (1151)
72 ocular hypertension/ep, mo or (ocular hypertension/ and (incidence/ or prevalence/ or age distribution/ or sex distribution/ or morbidity/ or mortality/)) (407)
73 ((ocular or intraocular) adj hypertens$ adj3 (incidence or prevalence or epidemiology or mortality)).ti,ab. (66)
74 glaucoma/ep, mo or (glaucoma/ and (incidence/ or prevalence/ or age distribution/ or sex distribution/ or morbidity/ or mortality/)) (1797)
75 glaucoma, angle-closure/ep, mo or (glaucoma, angle-closure/ and (incidence/ or prevalence/ or age distribution/ or sex distribution/ or morbidity/ or mortality/)) (324)
76 glaucoma, open-angle/ep, mo or (glaucoma, open-angle/ and (incidence/ or prevalence/ or age distribution/ or sex distribution/ or morbidity/ or mortality/)) (1024)
77 (glaucoma adj3 (incidence or prevalence or epidemiology or mortality)).ti,ab. (809)
78 optic nerve diseases/ep, mo or (optic nerve diseases/ and (incidence/ or prevalence/ or age distribution/ or sex distribution/ or morbidity/ or mortality/)) (366)
79 (optic nerve adj3 (incidence or prevalence or epidemiology or mortality)).ti,ab. (60)
80 exp optic atrophy/ep, mo or (exp optic atrophy/ and (incidence/ or prevalence/ or age distribution/ or sex distribution/ or morbidity/ or mortality/)) (242)
81 (optic atrophy adj3 (incidence or prevalence or epidemiology or mortality)).ti,ab. (22)

82 refractive errors/ep, mo or (refractive errors/ and (incidence/ or prevalence/ or age distribution/ or sex distribution/ or morbidity/ or mortality/)) (1118)
83 (refractive error$ adj3 (incidence or prevalence or epidemiology or mortality)).ti,ab. (326)
84 astigmatism/ep, mo or (astigmatism/ and (incidence/ or prevalence/ or age distribution/ or sex distribution/ or morbidity/ or mortality/)) (410)
85 (astigmatism adj3 (incidence or prevalence or epidemiology or mortality)).ti,ab. (200)
86 hyperopia/ep, mo or (hyperopia/ and (incidence/ or prevalence/ or age distribution/ or sex distribution/ or morbidity/ or mortality/)) (256)
87 (hyperopia adj3 (incidence or prevalence or epidemiology or mortality)).ti,ab. (107)
88 exp myopia/ep, mo or (exp myopia/ and (incidence/ or prevalence/ or age distribution/ or sex distribution/ or morbidity/ or mortality/)) (1303)
89 ((myopia or myopic) adj3 (incidence or prevalence or epidemiology or mortality)).ti,ab. (744)
90 retinal diseases/ep, mo or (retinal diseases/ and (incidence/ or prevalence/ or age distribution/ or sex distribution/ or morbidity/ or mortality/)) (847)
91 (retina$1 adj3 disease$ adj3 (incidence or prevalence or epidemiology or mortality)).ti,ab. (33)
92 diabetic retinopathy/ep, mo or (diabetic retinopathy/ and (incidence/ or prevalence/ or age distribution/ or sex distribution/ or morbidity/ or mortality/)) (3079)
93 (diabetic retinopath$ adj3 (incidence or prevalence or epidemiology or mortality)).ti,ab. (634)
94 retinal degeneration/ep, mo or (retinal degeneration/ and (incidence/ or prevalence/ or age distribution/ or sex distribution/ or morbidity/ or mortality/)) (149)
95 (retina$1 adj3 degenerat$ adj3 (incidence or prevalence or epidemiology or mortality)).ti,ab. (26)
96 exp macular degeneration/ep, mo or (exp macular degeneration/ and (incidence/ or prevalence/ or age distribution/ or sex distribution/ or morbidity/ or mortality/)) (2013)
97 (macular adj3 degenerat$ adj3 (incidence or prevalence or epidemiology or mortality)).ti,ab. (70)
98 retinal detachment/ep, mo or (retinal detachment/ and (incidence/ or prevalence/ or age distribution/ or sex distribution/ or morbidity/ or mortality/)) (756)
99 (retina$1 adj3 detach$ adj3 (incidence or prevalence or epidemiology or mortality)).ti,ab. (317)
100 retinal vein occlusion/ep, mo or (retinal vein occlusion/ and (incidence/ or prevalence/ or age distribution/ or sex distribution/ or morbidity/ or mortality/)) (285)
101 (retina$1 adj3 vein adj3 occlu$ adj3 (incidence or prevalence or epidemiology or mortality)).ti,ab. (48)
102 retinitis/ep, mo or (retinitis/ and (incidence/ or prevalence/ or age distribution/ or sex distribution/ or morbidity/ or mortality/)) (86)
103 (retinitis adj3 (incidence or prevalence or epidemiology or mortality)).ti,ab. (128)
104 chorioretinitis/ep, mo or (chorioretinitis/ and (incidence/ or prevalence/ or age distribution/ or sex distribution/ or morbidity/ or mortality/)) (94)
105 (chorioretinitis adj3 (incidence or prevalence or epidemiology or mortality)).ti,ab. (12)
106 cytomegalovirus retinitis/ep, mo or (cytomegalovirus retinitis/ and (incidence/ or prevalence/ or age distribution/ or sex distribution/ or morbidity/ or mortality/)) (194)
107 retinopathy of prematurity/ep, mo or (retinopathy of prematurity/ and (incidence/ or prevalence/ or age distribution/ or sex distribution/ or morbidity/ or mortality/)) (987)
108 (retinopathy adj3 prematurity adj3 (incidence or prevalence or epidemiology or mortality)).ti,ab. (248)
109 exp choroid diseases/ep, mo or (exp choroid diseases/ and (incidence/ or prevalence/ or age distribution/ or sex distribution/ or morbidity/ or mortality/)) (828)
110 (choroid adj3 disease$ adj3 (incidence or prevalence or epidemiology or mortality)).ti,ab. (0)
111 exp uveitis/ep, mo or (exp uveitis/ and (incidence/ or prevalence/ or age distribution/ or sex distribution/ or morbidity/ or mortality/)) (1886)
112 (uveitis adj3 (incidence or prevalence or epidemiology or mortality)).ti,ab. (248)
113 vision disorders/ep, mo or (vision disorders/ and (incidence/ or prevalence/ or age distribution/ or sex distribution/ or morbidity/ or mortality/)) (2636)

114 amblyopia/ep, mo or (amblyopia/ and (incidence/ or prevalence/ or age distribution/ or sex distribution/ or morbidity/ or mortality/)) (417)
115 (amblyopia adj3 (incidence or prevalence or epidemiology or mortality)).ti,ab. (220)
116 (population adj3 eye adj3 survey$).ti,ab. (29)
117 (population adj3 vision adj3 survey$).ti,ab. (1)
118 (population adj3 blindness adj3 survey$).ti,ab. (13)
119 (population adj3 visual adj3 survey$).ti,ab. (2)
120 animals/ not humans/ (4466715)
121 (letter or comment or editorial).pt. (1660989)
122 (clinical trial or controlled clinical trial).pt. (534257)
123 randomized controlled trial.pt. (468991)
124 review.pt. (2433961)
125 exp blindness/ or (blindness or (visual adj3 impair$) or (vision adj3 impair$)).ti,ab. (53846)
126 or/1-32 (17386)
127 or/33-115 (27402)
128 or/116-119 (45)
129 126 or (127 and 125) or 128 (18412)
130 129 not (120 or 121 or 122 or 123 or 124) (14219)

2.2.2 Embase search

Source: Embase
Interface / URL: Ovid
Database coverage dates: 1974 to 2018 October 2
Search date: 03/10/18
Retrieved records: 23973
Search strategy:

Database: Embase <1974 to 2018 October 2>
Search Strategy:
----
1 exp blindness/ep (1747)
2 exp blindness/ and (prevalence/ or incidence/ or morbidity/ or mortality/) (4126)
3 exp blindness/ and (age distribution/ or sex ratio/) (490)
4 exp blindness/ and exp eye disease/ep (2586)
5 exp blindness/ and (epidemiology or incidence or prevalence or mortality).ti,ab. (4791)
6 (blindness and (incidence or prevalence or epidemiology or mortality)).ti,ab. (5230)
7 vision/ and (age distribution/ or sex ratio/) (372)
8 (vision and (epidemiology or incidence or prevalence or mortality)).ti,ab. (10276)
9 vision/ and (incidence/ or prevalence/ or mortality/ or morbidity/) (1381)
10 exp visual acuity/ and (incidence/ or prevalence/ or mortality/ or morbidity/) (5303)
11 exp visual acuity/ and (age distribution/ or sex ratio/) (1277)
12 (visual acuity and (epidemiology or incidence or prevalence or mortality)).ti,ab. (7105)
13 binocular vision/ and (incidence/ or prevalence/ or morbidity/ or mortality/) (155)
14 binocular vision/ and (age distribution/ or sex ratio/) (38)
15 ((vision adj3 binocular) and (epidemiology or incidence or prevalence or mortality)).ti,ab. (163)
16 visual disorder/ep (862)
17 visual disorder/ and (incidence/ or prevalence/ or mortality/ or morbidity/) (2314)
18 visual disorder/ and (age distribution/ or sex ratio/) (345)
19 (low vision and (epidemiology or incidence or prevalence or mortality)).ti,ab. (573)

20 night blindness/ep (71)
21 night blindness/ and (incidence/ or prevalence/ or morbidity/ or mortality/) (195)
22 night blindness/ and (age distribution/ or sex ratio/) (17)
23 presbyopia/ep (56)
24 presbyopia/ and (incidence/ or prevalence/ or mortality/ or morbidity/) (108)
25 presbyopia/ and (age distribution/ or sex ratio/) (41)
26 (presbyopi$ and (epidemiology or incidence or prevalence or morbidity)).ti,ab. (142)
27 (visual$ adj3 impair$).ti,ab. and (incidence/ or prevalence/ or morbidity/ or mortality/) (2607)
28 (visual$ adj3 impair$).ti,ab. and (age distribution/ or sex ratio/) (435)
29 (visual$ adj3 impair$ adj3 (incidence or prevalence or epidemiolog$ or morbidity)).ti,ab. (642)
30 (vision adj3 impair$ adj3 (incidence or prevalence or epidemiolog$ or morbidity)).ti,ab. (120)
31 ((amaurosis or deaf-blind) adj3 (incidence or prevalence or epidemiolog$ or morbidity)).ti,ab. (11)
32 conjunctiva disease/ep (81)
33 conjunctiva disease/ and (incidence/ or prevalence/ or age distribution/ or sex ratio/ or morbidity/ or mortality/) (281)
34 conjunctivitis/ep (473)
35 conjunctivitis/ and (incidence/ or prevalence/ or age distribution/ or sex ratio/ or morbidity/ or mortality/) (1476)
36 ((conjunctivitis or conjunctival) adj3 (incidence or prevalence or epidemiolog$ or mortality)).ti,ab. (306)
37 newborn ophthalmia/ep (55)
38 newborn ophthalmia/ and (incidence/ or prevalence/ or age distribution/ or sex ratio/ or morbidity/ or mortality/) (72)
39 (ophthalmia adj3 (neonat$ or newborn) adj3 (incidence or prevalence or epidemiology or mortality)).ti,ab. (14)
40 trachoma/ep (775)
41 trachoma/ and (incidence/ or prevalence/ or age distribution/ or sex ratio/ or morbidity/ or mortality/) (865)
42 (trachoma adj3 (incidence or prevalence or epidemiology or mortality)).ti,ab. (438)
43 pterygium/ep (155)
44 pterygium/ and (incidence/ or prevalence/ or age distribution/ or sex ratio/ or morbidity/ or mortality/) (279)
45 (pterygium adj3 (incidence or prevalence or epidemiology or mortality)).ti,ab. (116)
46 xerophthalmia/ep (164)
47 xerophthalmia/ and (incidence/ or prevalence/ or age distribution/ or sex ratio/ or morbidity/ or mortality/) (276)
48 (xerophthalmia adj3 (incidence or prevalence or epidemiology or mortality)).ti,ab. (81)
49 cornea disease/ep (179)
50 cornea disease/ and (incidence/ or prevalence/ or age distribution/ or sex ratio/ or morbidity/ or mortality/) (532)
51 (cornea$ adj3 disease$ adj3 (incidence or prevalence or epidemiology or mortality)).ti,ab. (21)
52 cornea opacity/ep (59)
53 cornea opacity/ and (incidence/ or prevalence/ or age distribution/ or sex ratio/ or morbidity/ or mortality/) (370)
54 (cornea$ adj3 (opaque$ or opac$) adj3 (incidence or prevalence or epidemiology or mortality)).ti,ab. (34)
55 keratitis/ep (373)
56 keratitis/ and (incidence/ or prevalence/ or age distribution/ or sex ratio/ or morbidity/ or mortality/) (739)
57 (keratitis adj3 (incidence or prevalence or epidemiology or mortality)).ti,ab. (241)
58 cornea ulcer/ep (137)
59 cornea ulcer/ and (incidence/ or prevalence/ or age distribution/ or sex ratio/ or morbidity/ or mortality/) (313)
60 (cornea$ adj3 ulcer$ adj3 (incidence or prevalence or epidemiology or mortality)).ti,ab. (66)
61 keratoconus/ep (113)

62 keratoconus/ and (incidence/ or prevalence/ or age distribution/ or sex ratio/ or morbidity/ or mortality/) (346)
63 (keratoconus adj3 (incidence or prevalence or epidemiology or mortality)).ti,ab. (76)
64 (hereditary adj3 eye).ti,ab. and (incidence/ or prevalence/ or age distribution/ or sex distribution/ or morbidity/ or mortality/) (9)
65 (hereditary adj3 eye adj3 (incidence or prevalence or epidemiology or mortality)).ti,ab. (1)
66 retinitis pigmentosa/ep (135)
67 retinitis pigmentosa/ and (incidence/ or prevalence/ or age distribution/ or sex ratio/ or morbidity/ or mortality/) (329)
68 (retinitis pigmentosa adj3 (incidence or prevalence or epidemiology or mortality)).ti,ab. (42)
69 exp eye infection/ep (1601)
70 exp eye infection/ and (incidence/ or prevalence/ or age distribution/ or sex ratio/ or morbidity/ or mortality/) (2347)
71 (infection$ adj3 eye$1 adj3 (incidence or prevalence or epidemiology or mortality)).ti,ab. (19)
72 lens disease/ep (32)
73 lens disease/ and (incidence/ or prevalence/ or age distribution/ or sex ratio/ or morbidity/ or mortality/) (103)
74 (lens adj3 disease$ adj3 (incidence or prevalence or epidemiology or mortality)).ti,ab. (3)
75 exp aphakia/ep (43)
76 exp aphakia/ and (incidence/ or prevalence/ or age distribution/ or sex ratio/ or morbidity/ or mortality/) (243)
77 (aphakia adj3 (incidence or prevalence or epidemiology or mortality)).ti,ab. (15)
78 exp cataract/ep (1804)
79 exp cataract/ and (incidence/ or prevalence/ or age distribution/ or sex ratio/ or morbidity/ or mortality/) (4874)
80 (cataract$ adj3 (incidence or prevalence or epidemiology or mortality)).ti,ab. (1361)
81 exp glaucoma/ep (1873)
82 exp glaucoma/ and (incidence/ or prevalence/ or age distribution/ or sex ratio/ or morbidity/ or mortality/) (4768)
83 (glaucoma adj3 (incidence or prevalence or epidemiology or mortality)).ti,ab. (1005)
84 ((ocular hypertens$ or intraocular hypertens$) adj3 (incidence or prevalence or epidemiology or mortality)).ti,ab. (87)
85 optic nerve disease/ep (216)
86 optic nerve disease/ and (incidence/ or prevalence/ or age distribution/ or sex ratio/ or morbidity/ or mortality/) (868)
87 (optic nerve adj3 (incidence or prevalence or epidemiology or mortality)).ti,ab. (79)
88 exp optic nerve atrophy/ep (162)
89 exp optic nerve atrophy/ and (incidence/ or prevalence/ or age distribution/ or sex ratio/ or morbidity/ or mortality/) (599)
90 (optic adj3 atroph$ adj3 (incidence or prevalence or epidemiology or mortality)).ti,ab. (33)
91 refraction error/ep (667)
92 refraction error/ and (incidence/ or prevalence/ or age distribution/ or sex ratio/ or morbidity/ or mortality/) (1674)
93 (refract$ adj3 error$ adj3 (incidence or prevalence or epidemiology or mortality)).ti,ab. (390)
94 astigmatism/ep (289)
95 astigmatism/ and (incidence/ or prevalence/ or age distribution/ or sex ratio/ or morbidity/ or mortality/) (805)
96 (astigmatism adj3 (incidence or prevalence or epidemiology or mortality)).ti,ab. (229)
97 hypermetropia/ep (232)

98 hypermetropia/ and (incidence/ or prevalence/ or age distribution/ or sex ratio/ or morbidity/ or mortality/) (723)
99 ((hyperopia or hypermetropia) adj3 (incidence or prevalence or epidemiology or mortality)).ti,ab. (133)
100 exp myopia/ep (799)
101 exp myopia/ and (incidence/ or prevalence/ or age distribution/ or sex ratio/ or morbidity/ or mortality/) (1822)
102 ((myopia or myopic) adj3 (incidence or prevalence or epidemiology or mortality)).ti,ab. (864)
103 retina disease/ep (190)
104 retina disease/ and (incidence/ or prevalence/ or age distribution/ or sex ratio/ or morbidity/ or mortality/) (594)
105 (retina$1 adj3 disease$ adj3 (incidence or prevalence or epidemiology or mortality)).ti,ab. (40)
106 diabetic retinopathy/ep (1679)
107 diabetic retinopathy/ and (incidence/ or prevalence/ or age distribution/ or sex distribution/ or morbidity/ or mortality/) (4842)
108 (diabetic retinopath$ adj3 (incidence or prevalence or epidemiology or mortality)).ti,ab. (860)
109 retina degeneration/ep (67)
110 retina degeneration/ and (incidence/ or prevalence/ or age distribution/ or sex ratio/ or morbidity/ or mortality/) (193)
111 (retina$1 adj3 degenerat$ adj3 (incidence or prevalence or epidemiology or mortality)).ti,ab. (30)
112 exp retina maculopathy/ep (430)
113 exp retina maculopathy/ and (incidence/ or prevalence/ or age distribution/ or sex ratio/ or morbidity/ or mortality/) (2492)
114 (macular adj3 degenerat$ adj3 (incidence or prevalence or epidemiology or mortality)).ti,ab. (95)
115 retina detachment/ep (264)
116 retina detachment/ and (incidence/ or prevalence/ or age distribution/ or sex ratio/ or morbidity/ or mortality/) (1347)
117 (retina$1 adj3 detach$ adj3 (incidence or prevalence or epidemiology or mortality)).ti,ab. (366)
118 exp retina vein occlusion/ep (150)
119 exp retina vein occlusion/ and (incidence/ or prevalence/ or age distribution/ or sex ratio/ or morbidity/ or mortality/) (560)
120 (retina$1 adj3 vein adj3 occlu$ adj3 (incidence or prevalence or epidemiology or mortality)).ti,ab. (58)
121 exp retinitis/ep (661)
122 exp retinitis/ and (incidence/ or prevalence/ or age distribution/ or sex ratio/ or morbidity/ or mortality/) (2010)
123 (retinitis adj3 (incidence or prevalence or epidemiology or mortality)).ti,ab. (131)
124 (chorioretinitis adj3 (incidence or prevalence or epidemiology or mortality)).ti,ab. (13)
125 retrolental fibroplasia/ep (495)
126 retrolental fibroplasia/ and (incidence/ or prevalence/ or age distribution/ or sex ratio/ or morbidity/ or mortality/) (1348)
127 (((retinopathy adj3 prematurity) or retrolental fibroplasia) adj3 (incidence or prevalence or epidemiology or mortality)).ti,ab. (322)
128 exp choroid disease/ep (386)
129 exp choroid disease/ and (incidence/ or prevalence/ or age distribution/ or sex ratio/ or morbidity/ or mortality/) (1580)
130 (choroid adj3 disease$ adj3 (incidence or prevalence or epidemiology or mortality)).ti,ab. (0)
131 exp uveitis/ep (1234)
132 exp uveitis/ and (incidence/ or prevalence/ or age distribution/ or sex ratio/ or morbidity/ or mortality/) (3850)
133 (uveitis adj3 (incidence or prevalence or epidemiology or mortality)).ti,ab. (375)
134 visual disorder/ep (862)

135 visual disorder/ and (incidence/ or prevalence/ or age distribution/ or sex ratio/ or morbidity/ or mortality/) (2523)
136 amblyopia/ep (285)
137 amblyopia/ and (incidence/ or prevalence/ or age distribution/ or sex distribution/ or morbidity/ or mortality/) (693)
138 (amblyopia adj3 (incidence or prevalence or epidemiology or mortality)).ti,ab. (255)
139 (population adj3 eye adj3 survey$).ti,ab. (34)
140 (population adj3 vision adj3 survey$).ti,ab. (1)
141 (population adj3 blindness adj3 survey$).ti,ab. (17)
142 (population adj3 visual adj3 survey$).ti,ab. (3)
143 or/1-31 (28865)
144 or/32-138 (39144)
145 exp blindness/ or (blindness or (visual adj3 impair$) or (vision adj3 impair$)).ti,ab. (70451)
146 144 and 145 (5523)
147 143 or 146 or (or/139-142) (29282)
148 limit 147 to (amphibia or ape or bird or cat or cattle or chicken or dog or "ducks and geese" or fish or "frogs and toads" or goat or guinea pig or "hamsters and gerbils" or horse or monkey or mouse or "pigeons and doves" or "rabbits and hares" or rat or reptile or sheep or swine) (563)
149 limit 147 to animal studies (459)
150 limit 147 to animals (519)
151 limit 147 to human (27190)
152 (148 or 149 or 150) not 151 (595)
153 147 not 152 (28687)
154 (letter or editorial or review).pt. (3965506)
155 153 not 154 (23973)

2.2.3 WHOLIS search
Source: WHOLIS
Interface / URL: http://kohahq.searo.who.int/
Database coverage dates:
Search date: 04/10/18
Retrieved records: 79
Search strategy:

The search interface and functionality of WHOLIS has changed since the previous searches – hence the different structure in the update.
Advanced search
http://kohahq.searo.who.int/cgi-bin/koha/opac-search.pl
All terms search via Keyword field search
All searches date limited 1980 to current
Email to WHO Library confirmed that truncation is supported with the * character
Nested Boolean does not seem to be supported in this interface
All searches conducted individually and the results added to the "Cart". Here duplicate records were automatically removed – all results were downloaded as one batch of unique records.
Blindness AND epidemiolog* = 15 results
Blindness AND incidence = 1 result
Blindness AND prevalence = 3 results
Blindness AND morbidity = 1 result
Blindness AND mortality = 0 result
Visual AND epidemiolog = 6 results
Visual AND incidence = 3 results
Visual AND prevalence = 2 results
Visual AND morbidity = 2 results
Visual AND mortality = 4 results

Vision AND epidemiolog = 5 results
Vision AND incidence = 1 result
Vision AND prevalence = 1 result
Vision AND morbidity = 0 results
Vision AND mortality = 1 results
Acuity = 0 results
Amaurosis = 0 results
Conjunctiv* = 5 results
Ophthalmia = 1 result
Trachoma AND epidemiolog = 3 results
Trachoma AND incidence = 2 results
Trachoma AND prevalence = 3 results
Trachoma AND morbidity = 1 result
Trachoma AND mortality = 0 results
Pterygium = 1 result
Xerophthalmia AND epidemiolog = 2 results
Xerophthalmia AND incidence = 0 results
Xerophthalmia AND prevalence = 1 result
Xerophthalmia AND morbidity = 1 result
Xerophthalmia AND mortality = 1 result
Cornea* = 7 results
Keratitis = 2 results
Keratoconus = 0 results
Eye AND epidemiolog = 20 results
Eye AND incidence = 2 results
Eye AND prevalence = 7 results
Eye AND morbidity = 1 result
Eye AND mortality = 2 results
Lens AND epidemiolog = 1 result
Lens AND incidence = 2 results
Lens AND prevalence = 3 results
Lens AND morbidity = 0 results
Lens AND mortality = 0 results
Aphakia = 0 results
Cataract* = 10 results
Ocular AND epidemiolog = 5 results
Ocular AND incidence = 1 result
Ocular AND prevalence = 1 result
Ocular AND morbidity = 0 results
Ocular AND mortality = 0 results
Intraocular = 1 result
Glaucoma = 2 results
Optic* AND epidemiolog = 1 result
Optic* AND incidence = 0 results
Optic* AND prevalence = 0 results
Optic* AND morbidity = 0 results
Optic* AND mortality = 1 results
Refractive = 0 results
Astigmatism = 0 results
Hyperopia = 0 results
Hypermetropia = 0 results
Myopia = 3 results
Retinitis = 0 results
Retina* = 1 result
Retinopathy = 5 results
Macular = 3 results
Chorioretinitis = 0 results
Choroid = 0 results
Uveitis = 0 results
Amblyopia = 0 results

2.2.4 SciELO search

Source: SciELO Citation Index (SCIELO)
Interface / URL: Web of Science
Database coverage dates: 1997-2018. Last updated 27/09/18
Search date: 04/10/2018
Retrieved records: 709
Search strategy:

# 53 #52 OR #51 OR #50 709
Indexes=SCIELO Timespan=All years

# 52 #49 OR #48 OR #47 OR #46 0
Indexes=SCIELO Timespan=All years

# 51 #45 OR #44 OR #43 OR #42 OR #41 OR #40 OR #39 OR #38 OR #37 OR #36 OR #35 OR #34 OR #33 OR #32 OR #31 OR #30 OR #29 OR #28 OR #27 OR #26 OR #25 OR #24 OR #23 OR #22 OR #21 OR #20 OR #19 OR #18 OR #17 OR #16 OR #15 OR #14 OR #13 OR #12 OR #11 OR #10 177
Indexes=SCIELO Timespan=All years

# 50 #9 OR #8 OR #7 OR #6 OR #5 OR #4 OR #3 OR #2 OR #1 612
Indexes=SCIELO Timespan=All years

# 49 TS=("population" NEAR/3 "visual" NEAR/3 survey*) 0
Indexes=SCIELO Timespan=All years

# 48 TS=("population" NEAR/3 "blindness" NEAR/3 survey*) 0
Indexes=SCIELO Timespan=All years

# 47 TS=("population" NEAR/3 "vision" NEAR/3 survey*) 0
Indexes=SCIELO Timespan=All years

# 46 TS=("population" NEAR/3 "eye" NEAR/3 survey*) 0
Indexes=SCIELO Timespan=All years

# 45 TS=("amblyopia" NEAR/3 ("incidence" OR "prevalence" OR "epidemiology" OR "mortality")) 12
Indexes=SCIELO Timespan=All years

# 44 TS=("uveitis" NEAR/3 ("incidence" OR "prevalence" OR "epidemiology" OR "mortality")) 12
Indexes=SCIELO Timespan=All years

# 43 TS=("choroid" NEAR/3 disease* NEAR/3 ("incidence" OR "prevalence" OR "epidemiology" OR "mortality")) 0
Indexes=SCIELO Timespan=All years

# 42 TS=("retinopathy" NEAR/3 "prematurity" NEAR/3 ("incidence" OR "prevalence" OR "epidemiology" OR "mortality")) 19
Indexes=SCIELO Timespan=All years

# 41 TS=("chorioretinitis" NEAR/3 ("incidence" OR "prevalence" OR "epidemiology" OR "mortality")) 0
Indexes=SCIELO Timespan=All years

# 40 TS=("retinitis" NEAR/3 ("incidence" OR "prevalence" OR "epidemiology" OR "mortality")) 1
Indexes=SCIELO Timespan=All years

# 39 TS=(retina* NEAR/3 "vein" NEAR/3 occlu* NEAR/3 ("incidence" OR "prevalence" OR "epidemiology" OR "mortality")) 0
Indexes=SCIELO Timespan=All years

# 38 TS=(retina* NEAR/3 detach* NEAR/3 ("incidence" OR "prevalence" OR "epidemiology" OR "mortality")) 2
Indexes=SCIELO Timespan=All years

# 37 TS=(macular* NEAR/3 degenerat* NEAR/3 ("incidence" OR "prevalence" OR "epidemiology" OR "mortality")) 3
Indexes=SCIELO Timespan=All years

# 36 TS=(retina* NEAR/3 degenerat* NEAR/3 ("incidence" OR "prevalence" OR "epidemiology" OR "mortality")) 1
Indexes=SCIELO Timespan=All years

# 35 TS=("diabetic retinopath*" NEAR/3 ("incidence" OR "prevalence" OR "epidemiology" OR "mortality")) 30
Indexes=SCIELO Timespan=All years

# 34 TS=(retina* NEAR/3 disease* NEAR/3 ("incidence" OR "prevalence" OR "epidemiology" OR "mortality")) 3
Indexes=SCIELO Timespan=All years

# 33 TS=(("myopia" OR "myopic") NEAR/3 ("incidence" OR "prevalence" OR "epidemiology" OR "mortality")) 5
Indexes=SCIELO Timespan=All years

# 32 TS=("hyperopia" NEAR/3 ("incidence" OR "prevalence" OR "epidemiology" OR "mortality")) 4
Indexes=SCIELO Timespan=All years

# 31 TS=("astigmatism" NEAR/3 ("incidence" OR "prevalence" OR "epidemiology" OR "mortality")) 6
Indexes=SCIELO Timespan=All years

# 30 TS=("refractive error*" NEAR/3 ("incidence" OR "prevalence" OR "epidemiology" OR "mortality")) 11
Indexes=SCIELO Timespan=All years

# 29 TS=("optic atrophy" NEAR/3 ("incidence" OR "prevalence" OR "epidemiology" OR "mortality")) 0
Indexes=SCIELO Timespan=All years

# 28 TS=("optic nerve" NEAR/3 ("incidence" OR "prevalence" OR "epidemiology" OR "mortality")) 1
Indexes=SCIELO Timespan=All years

# 27 TS=("glaucoma" NEAR/3 ("incidence" OR "prevalence" OR "epidemiology" OR "mortality")) 11
Indexes=SCIELO Timespan=All years

# 26 TS=(("ocular hypertens*" OR "intraocular hypertens*") NEAR/3 ("incidence" OR "prevalence" OR "epidemiology" OR "mortality")) 2
Indexes=SCIELO Timespan=All years

# 25 TS=(cataract* NEAR/3 ("incidence" OR "prevalence" OR "epidemiology" OR "mortality")) 25
Indexes=SCIELO Timespan=All years

# 24 TS=("aphakia" NEAR/3 ("incidence" OR "prevalence" OR "epidemiology" OR "mortality")) 0
Indexes=SCIELO Timespan=All years

# 23 TS=("lens" NEAR/3 disease* NEAR/3 ("incidence" OR "prevalence" OR "epidemiology" OR "mortality")) 0
Indexes=SCIELO Timespan=All years

# 22 TS=(infection* NEAR/3 eye* NEAR/3 ("incidence" OR "prevalence" OR "epidemiology" OR "mortality")) 0
Indexes=SCIELO Timespan=All years

# 21 TS=("retinitis pigmentosa" NEAR/3 ("incidence" OR "prevalence" OR "epidemiology" OR "mortality")) 1
Indexes=SCIELO Timespan=All years

# 20 TS=("hereditary" NEAR/3 "eye" NEAR/3 ("incidence" OR "prevalence" OR "epidemiology" OR "mortality")) 0
Indexes=SCIELO Timespan=All years

# 19 TS=("keratoconus" NEAR/3 ("incidence" OR "prevalence" OR "epidemiology" OR "mortality")) 0
Indexes=SCIELO Timespan=All years

# 18 TS=(cornea* NEAR/3 ulcer* NEAR/3 ("incidence" OR "prevalence" OR "epidemiology" OR "mortality")) 0
Indexes=SCIELO Timespan=All years

# 17 TS=("keratitis" NEAR/3 ("incidence" OR "prevalence" OR "epidemiology" OR "mortality")) 7
Indexes=SCIELO Timespan=All years

# 16 TS=((cornea* NEAR/3 (opaque* OR opac*)) NEAR/3 ("incidence" OR "prevalence" OR "epidemiology" OR "mortality")) 1
Indexes=SCIELO Timespan=All years

# 15 TS=("corneal" NEAR/3 disease* NEAR/3 ("incidence" OR "prevalence" OR "epidemiology" OR "mortality")) 0
Indexes=SCIELO Timespan=All years

14 TS=("xerophthalmia" NEAR/3 ("incidence" OR "prevalence" OR "epidemiology" OR "mortality")) 0

Indexes=SCIELO Timespan=All years

# 13 TS=("pterygium" NEAR/3 ("incidence" OR "prevalence" OR "epidemiology" OR "mortality")) 7
Indexes=SCIELO Timespan=All years

# 12 TS=("trachoma" NEAR/3 ("incidence" OR "prevalence" OR "epidemiology" OR "mortality")) 20
Indexes=SCIELO Timespan=All years

# 11 TS=("ophthalmia neonatorum" NEAR/3 ("incidence" OR "prevalence" OR "epidemiology" OR "mortality")) 0
Indexes=SCIELO Timespan=All years

# 10 TS=(("conjunctivitis" OR "conjunctival") NEAR/3 ("incidence" OR "prevalence" OR epidemiolog* OR "mortality")) 10
Indexes=SCIELO Timespan=All years

# 9 TS=(("amaurosis" OR "deaf-blind") NEAR/3 ("incidence" OR "prevalence" OR epidemiolog* OR "morbidity")) 0
Indexes=SCIELO Timespan=All years

# 8 TS=(vision* NEAR/3 impair* NEAR/3 ("incidence" OR "prevalence" OR epidemiolog* OR "morbidity")) 0
Indexes=SCIELO Timespan=All years

# 7 TS=(visual* NEAR/3 impair* NEAR/3 ("incidence" OR "prevalence" OR epidemiolog* OR "morbidity")) 10
Indexes=SCIELO Timespan=All years

# 6 TS=(presbyopi* AND ("epidemiology" OR "incidence" OR "prevalence" OR "morbidity")) 7
Indexes=SCIELO Timespan=All years

# 5 TS=(("low vision") AND ("epidemiology" OR "incidence" OR "prevalence" OR "mortality")) 25
Indexes=SCIELO Timespan=All years

# 4 TS=(("vision" NEAR/3 "binocular") AND ("epidemiology" OR "incidence" OR "prevalence" OR "mortality")) 3
Indexes=SCIELO Timespan=All years

# 3 TS=("visual acuity" AND ("epidemiology" OR "incidence" OR "prevalence" OR "mortality")) 180
Indexes=SCIELO Timespan=All years

# 2 TS=("vision" AND ("epidemiology" OR "incidence" OR "prevalence" OR "mortality")) 378
Indexes=SCIELO Timespan=All years

1 TS=("blindness" AND ("epidemiology" OR "incidence" OR "prevalence" OR "mortality")) 156

2.2.5 OpenGrey search
Source: OpenGrey
Interface / URL: http://www.opengrey.eu/
Database coverage dates: 1997-
Search date: 09/10/18-10/10/18

Retrieved records: 54
Search strategy:

Each search line searched individually. The records were cut and pasted into a MS Word document as the option to export as an XML was not working. Duplicate records (those already found in OpenGrey by a previous search line) were excluded. The total of 54 identified records therefore excludes these duplicates.

blindness NEAR/5 (epidemiology OR incidence OR prevalence OR mortality) 1 result

(vision NEAR/5 (epidemiology OR incidence OR prevalence OR mortality) 2 results

(visual NEAR/5 (epidemiology OR incidence OR prevalence OR mortality) 5 results

(presbyopi* NEAR/5 (epidemiology OR incidence OR prevalence OR morbidity) 0 results

(visual* NEAR/5 impair* NEAR/5 (incidence OR prevalence OR epidemiolog* OR morbidity)) 2 results

(vision* NEAR/5 impair* NEAR/5 (incidence OR prevalence OR epidemiolog* OR morbidity)) 0 results

(amaurosis OR deaf-blind) NEAR/5 (incidence OR prevalence OR epidemiolog* OR morbidity) 0 results

conjunctiv* NEAR/5 (incidence OR prevalence OR epidemiolog* OR mortality) 3 results

ophthalmia neonatorum NEAR/5 (incidence OR prevalence OR epidemiology OR mortality) 0 results

trachoma NEAR/5 (incidence OR prevalence OR epidemiology OR mortality) 1 result

pterygium NEAR/5 (incidence OR prevalence OR epidemiology OR mortality) 0 results

xerophthalmia NEAR/5 (incidence OR prevalence OR epidemiology OR mortality) 0 results

cornea* NEAR/5 (incidence OR prevalence OR epidemiology OR mortality) 1 result

keratitis NEAR/5 (incidence OR prevalence OR epidemiology OR mortality) 1 result

keratoconus NEAR/5 (incidence OR prevalence OR epidemiology OR mortality) 1 result

eye NEAR/5 (incidence OR prevalence OR epidemiology OR mortality) 5 results

retinitis pigmentosa NEAR/5 (incidence OR prevalence OR epidemiology OR mortality)
2 results

lens NEAR/5 (incidence OR prevalence OR epidemiology OR mortality) 3 results

aphakia NEAR/5 (incidence OR prevalence OR epidemiology OR mortality) 0 results

cataract* NEAR/5 (incidence OR prevalence OR epidemiology OR mortality) 7 results

(intraocular OR ocular) NEAR/5 (incidence OR prevalence OR epidemiology OR mortality) 3 results

glaucoma NEAR/5 (incidence OR prevalence OR epidemiology OR mortality) 1 result

optic NEAR/5 (incidence OR prevalence OR epidemiology OR mortality) 2 results

refractive error* NEAR/5 (incidence OR prevalence OR epidemiology OR mortality) 11 results

astigmatism NEAR/5 (incidence OR prevalence OR epidemiology OR mortality) 0 results

hyperopia NEAR/5 (incidence OR prevalence OR epidemiology OR mortality) 0 results

(myopia OR myopic) NEAR/5 (incidence OR prevalence OR epidemiology OR mortality) 5 results

retina* NEAR/5 (incidence OR prevalence OR epidemiology OR mortality) 1 result

retinitis NEAR/5 (incidence OR prevalence OR epidemiology OR mortality) 0 results

retinopath* NEAR/5 (incidence OR prevalence OR epidemiology OR mortality) 3 results

macular* degenerat* NEAR/5 (incidence OR prevalence OR epidemiology OR mortality) 8 results

chorioretinitis NEAR/5 (incidence OR prevalence OR epidemiology OR mortality) 0 results

choroid NEAR/5 (incidence OR prevalence OR epidemiology OR mortality) 0 results

uveitis NEAR/5 (incidence OR prevalence OR epidemiology OR mortality) 0 results

amblyopia NEAR/5 (incidence OR prevalence OR epidemiology OR mortality) 0 results

"eye survey*" OR "vision survey*" OR "blindness survey*" OR "visual survey*" 4 results

2.2.6 World Health Organization search

Source: World Health Organization webpages
Interface / URL: http://www.who.int/
Database coverage dates: N/A
Search date: 10/10/18
Retrieved records: 9 (6 after duplicates removed)
Search strategy:

Blindness and visual impairment section of the webpage located using the "Health Topics" drop down menu http://www.who.int/blindness/vision-report/en/.

Content scanned by the information specialist for documents that may provide epidemiological data.

2.2.7 International Agency for the Prevention of Blindness search

Source: International Agency for the Prevention of Blindness
Interface / URL: https://www.iapb.org/
Database coverage dates: N/A
Search date: 10/10/18
Retrieved records: 38 (29 after duplicates removed)
Search strategy:

Document Library https://www.iapb.org/knowledge/document-library/ browsed by information specialist for potentially relevant publications likely to report epidemiological data.

2.2.8 International Council of Ophthalmology search

Source: International Council of Ophthalmology
Interface / URL: http://www.icoph.org/
Database coverage dates: N/A
Search date: 10/10/18
Retrieved records: 8 (6 after duplicates removed)
Search strategy:

Web pages browsed by information specialist for potentially relevant publications likely to report epidemiological data.

2.2.9: International Eye Foundation search

Source: International Eye Foundation
Interface / URL: www.iefusa.org/
Database coverage dates: N/A
Search date: 11/10/187
Retrieved records: 0
Search strategy:

Web pages browsed by information specialist for potentially relevant publications likely to report epidemiological data.

2.2.10: Commonwealth Eye Health Consortium search

Source: Commonwealth Eye Health Consortium
Interface / URL: http://cehc.lshtm.ac.uk/
Database coverage dates: N/A
Search date: 11/10/18
Retrieved records: 0
Search strategy:

Web pages browsed by information specialist for potentially relevant publications likely to report epidemiological data.

2.2.11: International Centre for Eye Health (ICEH) search

Source: International Centre for Eye Health (ICEH)
Interface / URL: http://iceh.lshtm.ac.uk/
Database coverage dates: N/A
Search date: 11/10/18
Retrieved records: 89 (26 after duplicates removed)

Search strategy:

Web pages in the “Research” section browsed by information specialist for potentially relevant publications likely to report epidemiological data.

These were all references to ICEH authored journal publications rather than non-journal reports and other grey literature.

2.2.12: Brien Holden Vision Institute search

Source: Brien Holden Vision Institute
Interface / URL: https://www.brienholdenvision.org/
Database coverage dates: N/A
Search date: 11/10/18
Retrieved records: 47 (14 after duplicates removed)
Search strategy:

Web pages in the "Public Health Research" section browsed by information specialist for potentially relevant publications likely to report epidemiological data.

2.12.13: Sightsavers search

Source: Sightsavers
Interface / URL: https://www.sightsavers.org/
Database coverage dates: N/A
Search date: 11/10/18
Retrieved records: 11 (7 after duplicates removed)
Search strategy:

Web pages in the "Research Centre" section browsed by information specialist for potentially relevant publications likely to report epidemiological data.

Text 2.3. Results and Discussion (distance visual acuity data sources)

The combined previous searches for this project had identified 19040 unique records:

- The original search in 2008 yielded 10871 records;
- The 2012 update retrieved an additional 4038 unique records;
- The 2013 update retrieved an additional 2228 unique records;
- The 2014 update retrieved an additional 1903 unique records.

The 2018 update searches identified 39290 records (Table 6). Following deduplication against a) each other and b) the 19040 results of the previous searches, and the removal of records published before 1980 (841 records), 10092 records remained.

All searches are compromises. Strategies trade off sensitivity against precision, and decisions are made by research teams about the numbers of records that they are able to process in the time available. The strategy developed is reasonably sensitive and precise. It could have been made more sensitive, for example by increasing the value of the adj operator (so that words could occur further apart but still be retrieved), by using AND rather than adj or by using ep as a floating subheading that is not linked to specific MeSH. Sensitivity might also have been increased by identifying more synonyms and spelling variants for the eye diseases which may result in blindness. These approaches would have generated many more studies to assess for relevance, but might have retrieved additional relevant studies.

The strategy could also have been made more precise perhaps by omitting some of the combinations of eye diseases and epidemiology terms as text words in the title and abstract. However, increasing search precision usually risks losing relevant studies.

Text 2.4. Systematic review of studies of presbyopia prevalence

For the updated systematic review, the search strategy for distance sources included the 'presbyopia' term and studies that we identified reporting presbyopia prevalence were added to the database.

Only population-based surveys with one or more measurements of near vision and/or the prevalence of spectacle wear for presbyopia were included. On full text review, papers were additionally excluded if they did not specify the number of eligible participants or participation rate, if data were from a specific population that could not be generalized to the population as a whole or there was an unspecified method for determining whether near vision impairment was the result of ocular disease or from refractive error.

Text 3. Methods for comparability of vision impairment definition

MR-BRT [2] was used to adjust non-reference data to the reference definition of presenting vision data that fit within the WHO severity categories using data from studies that did not involve RAAB methodology. Data that spanned thresholds (either prevalence data for moderate and severe vision loss combined, or severe vision loss and blindness combined) were split into reference severity groups (moderate vision loss, severe vision loss, blindness) using a log ratio meta-regression with a cubic spline on age with linear tails. The input data for this meta-regression came from studies that provided matched age, year, sex and location data for each severity level (for example, moderate vision loss and severe vision loss separately).

Figure 2 shows, across ages, model fit with grey shading for uncertainty intervals overlaid with the model input data. Input data that were trimmed from the model are shown in red. Figure 2 demonstrates that the ratio of moderate to severe vision loss prevalence got smaller as age increased, whereas the ratio of blindness to severe vision loss got larger as age increased.

Studies that reported best-corrected visual acuity only were adjusted to the reference definition of presenting visual acuity, and studies that were collected using RAAB methodology were adjusted to the reference definition of non-RAAB vision tests using logit difference meta-regressions by severity level. The beta coefficients from the rapid and best-corrected meta-regressions for each severity level are shown in the Table 7. A negative coefficient indicates that the data were adjusted upward.

The best-corrected adjustment impacted a proportion of the data for each model: 11 of 60 sources for mild vision loss, 51 of 367 sources for moderate vision loss, 61 of 353 sources for severe vision loss, and 70 of 437 sources for blindness. The rapid methodology adjustment impacted a larger proportion of the data for each model: 29 of 60 sources for mild vision loss, 212 of 367 sources for moderate vision loss, 210 of 353 sources for severe vision loss, and 236 of 437 sources for blindness.

Text 4. Methods for comparability of data aggregated by age

If input data were collected for age ranges of greater than or equal to 25 years, these data were split into 5-year age bins using the super-regional age pattern generated in preliminary models that only included input data with age ranges of less than or equal to 25 years for a given severity and cause of vision loss. An inherent flaw in the method of age splitting is that some wide age data points after age splitting can reach implausibly high values at oldest ages. We decided in those cases not to age split the data.

Text 5. Methods for calculating prevalence of vision impairment by country, year, age, and sex

Studies that only reported "both" sex information were split into male- and female-specific data points by identifying within-study data points matched on age, year, and location that did report male and female data separately, and then using the log ratio of female to male prevalence from these studies as input data into a mixed-effects meta-regression tool developed at the IHME called MR-BRT (Meta Regression; Bayesian; Regularized; Trimmed). This tool was developed in part to allow for the ability to propagate between-study heterogeneity as part of the uncertainty adjustment, and to allow trimming of outlier input data. A detailed description of MR-BRT has been published elsewhere.[2] Results of this model and demographics data on population by location were used to determine male prevalence:

$$prev_{male} = prev_{both} * \frac{pop_{both}}{(pop_{male} + ratio * pop_{female})}$$

And then female prevalence:

$$prev_{female} = ratio * prev_{male}$$

For Dismod modeling, all input data from all locations were used in a mixed effects nonlinear model for a global estimate of disease burden. Model outputs (global fit plus fixed effects plus random effects) were used in a cascade as a prior for estimates in seven super-regions, which in turn were used as priors for 21 regional estimates, and then country estimates, and finally subnational estimates for a subset of countries. Final estimates for each geographical level were calculated by aggregation, where the country final was the sum of subnational estimates, regional final was the sum of country final estimates, etc. For Dismod modelling, predictive covariates included sociodemographic index and/or healthcare access quality index, and sex, and remaining unexplained variance went into random effects for locations with input data. Table 8 presents the fixed effects for sex (study-level covariate), healthcare access quality index (country-level covariate), and/or sociodemographic index that were included in Dismod models.

Age-standardization was computed using a standard population age structure that is updated in each GBD round. Currently, the standard population is taken as the average of age-specific distributions (non-weighted) from GBD 2019 population estimates for countries with at least 5 million people in the year 2019.

Text 6. Calculation of Years Lived with Disability (YLDs)

Final estimates for vision loss were used to calculate Years Lived with Disability (YLDs) based on disability weights assigned to each severity of vision loss, and, finally, adjusted for comorbidity with any other causes of non-fatal health loss. The health states and corresponding disability weights for vision are listed in the Table 9.

Text 7. GATHER compliance and access to code used in the analytical model

Estimates were produced in compliance with the Guidelines for Accurate and Transparent Health Estimates Reporting (GATHER).[6] Access to the GBD 2019 code (including Dismod engine and wrapper)can be obtained via this link:
http://ghdx.healthdata.org/gbd-2019/code

References:

[1] Global burden of 359 diseases, injuries, and impairments, 1990-2019: a systematic analysis for the Global Burden of Disease Study 2019" by GBD 2019 Diseases, Injuries, and Impairments Collaborators, in press.

[2] Zheng P, Aravkin AY, Barber R, Sorensen RJD, Murray CJL. Trimmed Constrained Mixed Effects Models: Formulations and Algorithms. 25 Sept 2019. https://arxiv.org/abs/1909.10700 (accessed 20 June 2020).

[3] Bourne R, Price H, Taylor H. New Systematic Review Methodology for Visual Impairment and Blindness for the 2010 Global Burden of Disease Study. Ophthalmic Epidemiology, 20(1):33–39, 2013.

[4] Bourne RRA, Flaxman SR, Braithwaite T, et al. Magnitude, temporal trends, and projections of the global prevalence of blindness and distance and near vision impairment: a systematic review and meta-analysis. Lancet Glob Health 2017; 5(9): e888-e97.

[5] Holden Brien A, Fricke Timothy R, Ho S May, Wong Reg, Schlenther Gerhard, Cronj´e Sonja, Burnett Anthea, Papas Eric, Naidoo Kovin S, Frick Kevin D. Global vision impairment due to uncorrected presbyopia. Archives of ophthalmology, 126(12):1731–1739, 2008.

[6] Stevens GA, Alkema L, Black RE, Boerma JT, Collins GS, Ezzati M, Grove JT, Hogan DR, Hogan MC, Horton R, Lawn JE, Marušić A, Mathers CD, Murray CJ, Rudan I, Salomon JA, Simpson PJ, Vos T, Welch V; (The GATHER Working Group). Guidelines for Accurate and Transparent Health Estimates Reporting: the GATHER statement. Lancet. 2016 Dec 10;388(10062):e19-e23.

Table 1. Countries and territories in analysis regions.

Central Europe, eastern Europe, and central Asia	
Central Asia	Armenia, Azerbaijan, Georgia, Kazakhstan, Kyrgyzstan, Mongolia, Tajikistan, Turkmenistan, Uzbekistan
Central Europe	Albania, Bosnia and Herzegovina, Bulgaria, Croatia, Czech Republic, Hungary, Montenegro, North Macedonia, Poland (subnational), Romania, Serbia, Slovakia, Slovenia
Eastern Europe	Belarus, Estonia, Latvia, Lithuania, Moldovia, Russia (subnational), Ukraine
High income	
Australasia	Australia, New Zealand (subnational Maori + non-Maori)
High-income Asia Pacific	Brunei, Japan (subnational), Singapore, South Korea
High-income North America	Canada, Greenland, United States (subnational)
Southern Latin America	Argentina, Chile, Uruguay
Western Europe	Andorra, Austria, Belgium, Cyprus, Denmark, Finland, France, Germany, Greece, Iceland, Ireland, Israel, Italy (subnational), Luxembourg, Malta, Monaco, Netherlands, Norway (subnational), Portugal, Sweden (subnational Stockholm + not Stockholm), United Kingdom (subnational; two levels of subnats)
Latin America and Caribbean	
Andean Latin America	Bolivia, Ecuador, Peru
Caribbean	Antigua and Barbuda, Bahamas, Barbados, Belize, Bermuda, Cuba, Dominica, Dominican Republic, Grenada, Guyana, Haiti, Jamaica, Puerto Rico, Saint Kitts and Nevis, Saint Lucia, Saint Vincent and the Grenadines, Suriname, Trinidad and Tobago, US Virgin Islands
Central Latin America	Colombia, Costa Rica, El Salvador, Guatemala, Honduras, Mexico (subnational), Nicaragua, Panama, Venezuela
Tropical Latin America	Brazil (subnational), Paraguay
North Africa and Middle East	

North Africa and Middle East	Afghanistan, Algeria, Bahrain, Egypt, Iran (subnational), Iraq, Jordan, Kuwait, Lebanon, Libya, Morocco, Oman, Palestine, Qatar, Saudi Arabia, Sudan, Syria, Tunisia, United Arab Emirates, Yemen
South Asia	
South Asia	Bangladesh, Bhutan, India (subnational; by state + rural and urban), Nepal, Pakistan (subnational)
Southeast Asia, east Asia, Oceania	
East Asia	China, North Korea, Taiwan (province of China)
Oceania	American Samoa, Cook Islands, Federated States of Micronesia, Fiji, Guam, Kiribati, Marshall Islands, Nauru, Niue, Northern Mariana Islands, Palau, Papua New Guinea, Samoa, Solomon Islands, Tokelau, Tonga, Tuvalu, Vanuatu
Southeast Asia	Cambodia, Indonesia (subnational), Laos, Malaysia, Maldives, Mauritius, Myanmar, Philippines (subnational), Seychelles, Sri Lanka, Thailand, Timor-Leste, Vietnam
Sub-Saharan Africa	
Central sub-Saharan Africa	Angola, Central African Republic, Congo (Brazzaville), Democratic Republic of the Congo, Equatorial Guinea, Gabon
Eastern sub-Saharan Africa	Burundi, Comoros, Djibouti, Eritrea, Ethiopia (subnational), Kenya (subnational), Madagascar, Malawi, Mozambique, Rwanda, Somalia, South Sudan, Tanzania, Uganda, Zambia
Southern sub-Saharan Africa	Botswana, eSwatini, Lesotho, Namibia, South Africa (subnational)
Western sub-Saharan Africa	Benin, Burkina Faso, Cape Verde, Cameroon, Chat, Côte D'Ivoire, Gambia, Ghana, Guinea, Guinea-Bissau, Liberia, Mali, Mauritania, Niger, Nigeria (subnational), São Tomé and Príncipe, Senegal, Sierra Leone, Togo

Table 2. Characteristics of data sources used in the analysis that reported distance vision impairment.

Country	Coverage	Rapid	Presenting or best-corrected	Core visual acuity level(s)	Year study started	Year study ended	Reference
Afghanistan	Subnational	Yes	Presenting	MSVI, blind, moderate, severe	2010	2010	Comprehensive Health and Education Forum International (CHEF) (Pakistan), International Centre for Eye Health (ICEH). Afghanistan Rapid Assessment of Avoidable Blindness 2010.
Argentina	National	Yes	Presenting	blind, moderate, severe	2013	2013	Barrenechea R, de la Fuente I, Plaza RG, Flores N, Segovia L, Villagómez Z, Camarero EE, Zepeda-Romero LC, Lansingh VC, Limburg H, Silva JC. National survey of blindness and avoidable visual impairment in Argentina, 2013. Rev Panam Salud Publica. 2015; 37(1): 7-12.
Argentina	Subnational	Yes	Both	MSVI, blind, moderate, severe	2003	2003	International Centre for Eye Health (ICEH). Argentina - Buenos Aires Rapid Assessment of Avoidable Blindness 2003. Grootebroek, Netherlands: RAAB Repository.
Argentina	National	Yes	Both	MSVI, blind, moderate, severe	2013	2013	International Centre for Eye Health (ICEH). Argentina Rapid Assessment of Avoidable Blindness Survey 2013. Grootebroek, Netherlands: RAAB Repository, 2013.
Australia	Subnational	No	Best-corrected	blind, moderate, severe	1992	1992	Casson R, Giles L, Newland HS. Prevalence of blindness and visual impairment in an elderly urban population. Aust N Z J Ophthalmol. 1996; 24(3): 239-43.
Australia	Subnational	No	Presenting	blind, moderate, severe	1997	1999	Chia E-M, Mitchell P, Rochtchina E, Foran S, Golding M, Wang JJ. Association between vision and hearing impairments and their combined effects on quality of life. Arch

							Ophthalmol. 2006; 124(10): 1465-70.
Australia	Subnational	No	Presenting	blind, mild, moderate, severe	1992	1999	Dimitrov PN, Mukesh BN, McCarty CA, Taylor HR. Five-year incidence of bilateral cause-specific visual impairment in the Melbourne Visual Impairment Project. Invest Ophthalmol Vis Sci. 2003; 44(12): 5075-81.
Australia	Subnational	No	Presenting	blind, moderate, severe	1992	1994	Foran S, Wang JJ, Mitchell P. Causes of visual impairment in two older population cross-sections: the Blue Mountains Eye Study. Ophthalmic Epidemiol. 2003; 10(4): 215-25.
Australia	Subnational	No	Presenting	blind, moderate, severe	2007	2009	Pai AS-I, Wang JJ, Samarawickrama C, Burlutsky G, Rose KA, Varma R, Wong TY, Mitchell P. Prevalence and risk factors for visual impairment in preschool children the Sydney Paediatric Eye Disease Study. Ophthalmology. 2011; 118(8): 1495-500.
Australia	Subnational	No	Presenting	blind, mild, moderate, near vision loss, severe	1992, 1994	1994, 1996	Taylor HR, Livingston PM, Stanislavsky YL, McCarty CA. Visual impairment in Australia: distance visual acuity, near vision, and visual field findings of the Melbourne Visual Impairment Project. Am J Ophthalmol. 1997; 123(3): 328-37.
Australia	Subnational	No	Presenting	blind, moderate, severe	1992, 1996	1995, 1996	VanNewkirk MR, Weih L, McCarty CA, Taylor HR. Cause-specific prevalence of bilateral visual impairment in Victoria, Australia: the Visual Impairment Project. Ophthalmology. 2001; 108(5): 960-7.
Australia	Subnational	No	Best-corrected	blind, moderate, severe	1992	1994	Wang JJ, Foran S, Mitchell P. Age-specific prevalence and causes of bilateral and unilateral visual impairment in older Australians: the Blue Mountains Eye Study. Clin Experiment

							Ophthalmol. 2000; 28(4): 268-73.
Bangladesh	Subnational	Yes	Both	MSVI, blind, moderate, severe	2013	2013	CSF Global (Bangladesh), International Centre for Eye Health (ICEH). Bangladesh - Barisal Rapid Assessment of Avoidable Blindness 2013.
Bangladesh	Subnational	Yes	Presenting	MSVI, blind, moderate, severe	2012	2012	CSF Global (Bangladesh), International Centre for Eye Health (ICEH). Bangladesh - Brahmanbaria and Satkhira Districts Rapid Assessment of Avoidable Blindness 2012.
Bangladesh	Subnational	Yes	Both	MSVI, blind, moderate, severe	2010	2010	CSF Global (Bangladesh), International Centre for Eye Health (ICEH). Bangladesh - Gazipur, Kishoreganj, and Cox's Bazar Districts Rapid Assessment of Avoidable Blindness 2010. Grootebroek, Netherlands: RAAB Repository.
Bangladesh	Subnational	Yes	Both	MSVI, blind, moderate, severe	2010	2010	CSF Global (Bangladesh), International Centre for Eye Health (ICEH). Bangladesh - Narail and Jamalpur Districts Rapid Assessment of Avoidable Blindness 2010.
Bangladesh	Subnational	Yes	Both	MSVI, blind, moderate, severe	2005	2005	CSF Global (Bangladesh), International Centre for Eye Health (ICEH). Bangladesh - Satkhira District Rapid Assessment of Avoidable Blindness 2005. Grootebroek, Netherlands: RAAB Repository.
Bangladesh	Subnational	Yes	Both	MSVI, blind, moderate, severe	2011	2011	CSF Global (Bangladesh), International Centre for Eye Health (ICEH). Bangladesh - Tangail Rapid Assessment of Avoidable Blindness 2011. Grootebroek, Netherlands: RAAB Repository.

Bangladesh	National	No	Both	MSVI, blind, mild, moderate, severe	1999	2000	International Centre for Eye Health (ICEH), National Institute of Ophthalmology (Bangladesh). Bangladesh National Blindness and Low Vision Prevalence Survey 1999-2000.
Bangladesh	Subnational	Yes	Both	MSVI, blind, moderate, severe	2011	2011	International Centre for Eye Health (ICEH). Bangladesh - Kushtia Rapid Assessment of Avoidable Blindness Survey 2011. Grootebroek, Netherlands: RAAB Repository, 2011.
Bangladesh	Subnational	Yes	Presenting	MSVI, blind, moderate, severe	2005	2005	Wadud Z, Kuper H, Polack S, Lindfield R, Akm MR, Choudhury KA, Lindfield T, Limburg H, Foster A. Rapid assessment of avoidable blindness and needs assessment of cataract surgical services in Satkhira District, Bangladesh. Br J Ophthalmol. 2006; 90(10): 1225-9.
Bangladesh, Thailand	National	No	Presenting	MSVI, moderate	1994, 1999	1995, 2000	Bourne R, Global Burden of Disease Vision Loss Expert Group. Vision Loss Database - Survey Data on Vision Loss by Severity and Etiology. [Unpublished].
Barbados	National	No	Best-corrected	blind, moderate, severe	1988	1992	Hyman L, Wu SY, Connell AM, Schachat A, Nemesure B, Hennis A, Leske MC. Prevalence and causes of visual impairment in The Barbados Eye Study. Ophthalmology. 2001; 108(10): 1751-6.
Bhutan	National	Yes	Both	MSVI, blind, moderate, severe	2009	2009	International Centre for Eye Health (ICEH). Bhutan Rapid Assessment of Avoidable Blindness Survey 2009. Grootebroek, Netherlands: RAAB Repository, 2009.
Bhutan	National	Yes	Presenting	MSVI, blind, moderate, severe	2010	2012	Lepcha NT, Chettri CK, Getshen K, Rai BB, Ramaswamy SB, Saibaba S, Nirmalan PK, Demarchis EH, Tabin G,

							Morley M, Morley K. Rapid assessment of avoidable blindness in Bhutan. Ophthalmic Epidemiol. 2013; 20(4): 212-9.
Botswana	National	Yes	Presenting	MSVI, blind, moderate, severe	2013	2014	International Centre for Eye Health (ICEH). Botswana Rapid Assessment of Avoidable Blindness 2013-2014. Grootebroek, Netherlands: RAAB Repository.
Brazil	Subnational	No	Both	MSVI, blind, severe	2002	2002	Araújo Filho A, Salomão SR, Berezovsky A, Cinoto RW, Morales PHA, Santos FRG, Belfort R Jr. Prevalence of visual impairment, blindness, ocular disorders and cataract surgery outcomes in low-income elderly from a metropolitan region of São Paulo-Brazil. *Arq Bras Oftalmol*. 2008; 71(2): 246-53.
Brazil	Subnational	No	Presenting	blind, mild, moderate, severe	2014	2015	Furtado JM, Berezovsky A, Ferraz NN, Muñoz S, Fernandes AG, Watanabe SS, Cunha CC, Vasconcelos GC, Sacai PY, Cypel M, Mitsuhiro MH, Morales PH, Cohen MJ, Campos M, Cohen JM, Belfort R Jr, Salomão SR. Prevalence and Causes of Visual Impairment and Blindness in Adults Aged 45 Years and Older from Parintins: The Brazilian Amazon Region Eye Survey. Ophthalmic Epidemiol. 2019; 1-10.
Brazil	Subnational	Yes	Both	MSVI, blind, moderate, severe	2003, 2004	2003, 2004	International Centre for Eye Health (ICEH). Brazil - Campinas Rapid Assessment of Avoidable Blindness 2004. Grootebroek, Netherlands: RAAB Repository.
Brazil	Subnational	No	Presenting	blind, moderate, severe	2004	2004	Salomao SR, Cinoto RW, Berezovsky A, Araujo-Filho A, Mitsuhiro MRKH, Mendieta L, Morales PHA, Pokharel GP, Belfort R Jr, Ellwein

							LB. Prevalence and causes of vision impairment and blindness in older adults in Brazil: the Sao Paulo Eye Study. Ophthalmic Epidemiol. 2008; 15(3): 167-75.
Brazil	Subnational	No	Presenting	MSVI, blind, moderate, severe	2006	2007	Schellini SA, Durkin SR, Hoyama E, Hirai F, Cordeiro R, Casson RJ, Selva D, Padovani CR. Prevalence and causes of visual impairment in a Brazilian population: the Botucatu Eye Study. BMC Ophthalmol. 2009; 9: 8.
Bulgaria	Subnational	No	Presenting	MSVI, blind, moderate, severe	1993	1993	Vassileva P, Gieser SC, Vitale S, Cholakova T, Katz J, West S. Blindness and visual impairment in western Bulgaria. Ophthalmic Epidemiol. 1996; 3(3): 143-9.
Burkina Faso	Subnational	Yes	Both	MSVI, blind, moderate, severe	2011	2011	International Centre for Eye Health (ICEH). Burkina Faso - West Central Rapid Assessment of Avoidable Blindness 2011. Grootebroek, Netherlands: RAAB Repository.
Burundi	Subnational	Yes	Both	MSVI, blind, moderate, severe	2010	2010	International Centre for Eye Health (ICEH). Burundi - Ngozi and Kayanza Rapid Assessment of Avoidable Blindness 2010. Grootebroek, Netherlands: RAAB Repository.
Burundi	Subnational	Yes	Presenting	MSVI, blind, moderate, severe	2009, 2010	2010	Kandeke L, Mathenge W, Giramahoro C, Undendere F-PA, Ruhagaze P, Habiyakare C, Courtright P, Lewallen S. Rapid assessment of avoidable blindness in two northern provinces of Burundi without eye services. Ophthalmic Epidemiol. 2012; 19(4): 211-5.
Cabo Verde	National	No	Presenting	MSVI, blind, moderate, severe	1998	1998	Schémann JF, Inocencio F, de Lourdes Monteiro M, Andrade J, Auzemery A, Guelfi Y. Blindness and low vision in Cape Verde Islands: results of

							a national eye survey. *Ophthalmic Epidemiol*. 2006; 13(4): 219-26.
Cambodia	Subnational	Yes	Presenting	MSVI, blind, moderate, severe	2002	2002	International Centre for Eye Health (ICEH), Subcommittee for the Prevention of Blindness (Cambodia). Cambodia - Battambang Rapid Assessment of Cataract Surgical Services 2002.
Cambodia	Subnational	Yes	Presenting	MSVI, blind, moderate, severe	2002	2002	International Centre for Eye Health (ICEH), Subcommittee for the Prevention of Blindness (Cambodia). Cambodia - Kampong Cham Rapid Assessment of Cataract Surgical Services 2002.
Cambodia	Subnational	Yes	Presenting	MSVI, blind, moderate, severe	2002	2002	International Centre for Eye Health (ICEH), Subcommittee for the Prevention of Blindness (Cambodia). Cambodia - Kampot Rapid Assessment of Cataract Surgical Services 2002.
Cambodia	Subnational	Yes	Both	MSVI, blind, moderate, severe	2011	2012	International Centre for Eye Health (ICEH). Cambodia - Takeo Rapid Assessment for Avoidable Blindness 2011-2012. Grootebroek, Netherlands: RAAB Repository.
Cambodia	National	Yes	Presenting	MSVI, blind, moderate, severe	2007	2007	International Centre for Eye Health (ICEH). Cambodia Rapid Assessment for Avoidable Blindness 2007.
Cambodia	Subnational	No	Presenting	MSVI, blind, moderate, severe	1996	1996	Rutzen AR, Ellish NJ, Schwab L, Graham PJ, Pizzarello LD, Hemady RK, Maldonado MJ. Blindness and eye disease in Cambodia. Ophthalmic Epidemiol. 2007; 14(6): 360-6.
Cameroon	Subnational	No	Presenting	blind, moderate, severe	2013	2013	Oye J, Mactaggart I, Polack S, Schmidt E, Tamo V, Okwen M, Kuper H. Prevalence and Causes of Visual Impairment in Fundong District, North West Cameroon: Results of a Population-Based Survey. Ophthalmic

							Epidemiol. 2017; 24(6): 394-400.
Cameroon	Subnational	Yes	Presenting	MSVI, blind, moderate, severe	2005	2005	Oye JE, Kuper H, Dineen B, Befidi-Mengue R, Foster A. Prevalence and causes of blindness and visual impairment in Muyuka: a rural health district in South West Province, Cameroon. Br J Ophthalmol. 2006; 90(5): 538-42.
Cameroon	Subnational	Yes	Presenting	MSVI, blind, moderate, severe	2005	2005	Oye JE, Kuper H. Prevalence and causes of blindness and visual impairment in Limbe urban area, South West Province, Cameroon. Br J Ophthalmol. 2007; 91(11): 1435-9.
Cameroon	Subnational	No	Presenting	blind, moderate, severe	1992	1992	Wilson MR, Mansour M, Ross-Degnan D, Moukouri E, Fobi G, Alemayehu W, Martone JF, Casey R, Bazargan M. Prevalence and causes of low vision and blindness in the Extreme North Province of Cameroon, West Africa. Ophthalmic Epidemiol. 1996; 3(1): 23-33.
Chile	Subnational	Yes	Both	MSVI, blind, moderate, severe	2006	2006	International Centre for Eye Health (ICEH). Chile - Biobio Rapid Assessment of Avoidable Blindness 2006. Grootebroek, Netherlands: RAAB Repository.
Chile, China, India, Japan, Malaysia, Nepal, South Africa	Subnational	No	Both	MSVI, blind, moderate, severe	1997, 1998, 2000, 2002, 2003	1998, 2000, 2001, 2002, 2003	Gilbert CE, Ellwein LB. Prevalence and causes of functional low vision in school-age children: results from standardized population surveys in Asia, Africa, and Latin America. Invest Ophthalmol Vis Sci. 2008; 49(3): 877-81.
China	Subnational	No	Presenting	MSVI, blind, moderate, severe	1987, 2006	1987, 2006	Chen H, Wu X, Wei M, Eichner JE, Fan Y, Zhang Z, Lei C, Stone DU, Yang J. Changes in the prevalence of visual impairment due to blinding trachoma in Sichuan province, China: a comparative study between 1987 and 2006.

							Ophthalmic Epidemiol. 2012; 19(1): 29-37.
China	Subnational	No	Both	MSVI, blind, moderate, severe	2016	2017	Chen X, Zhou D, Shen J, Wu Y, Sun Q, Dong J, Yu J. Prevalence and Causes of Visual Impairment in Adults in Binhu District, Wuxi, China. Med Sci Monit. 2018; 24: 317-323.
China	Subnational	No	Both	MSVI, blind, moderate, near vision loss, severe	2009	2009	Cheng F, Shan L, Song W, Fan P, Yuan H. Distance- and near-visual impairment in rural Chinese adults in Kailu, Inner Mongolia. Acta Ophthalmol. 2016; 94(4): 407-13.
China	Subnational	No	Presenting	blind, moderate, severe	2007	2007	Congdon N, Wang Y, Song Y, Choi K, Zhang M, Zhou Z, Xie Z, Li L, Liu X, Sharma A, Wu B, Lam DSC. Visual disability, visual function, and myopia among rural Chinese secondary school children: the Xichang Pediatric Refractive Error Study (X-PRES)–Report 1. *Invest Ophthalmol Vis Sci*. 2008; 49(7): 2888-94.
China	Subnational	No	Presenting	blind, moderate, severe	2015	2015	Gan S, Zhou X, Yan J, Liu X, Yi J, Zhou X, Liu D, Xie Q, Geng J, Lu Y. The prevalence and risk factors of visual impairment among rural residents aged 50 years and above in Yugan county, China. Ophthalmic Epidemiol. 2018; 25(5-6): 331-337.
China	Subnational	No	Both	blind, moderate, severe	2005	2005	He M, Huang W, Zheng Y, Huang L, Ellwein LB. Refractive error and visual impairment in school children in rural southern China. Ophthalmology. 2007; 114(2): 374-82.
China	Subnational	No	Both	blind, moderate, severe	2002	2003	He M, Zeng J, Liu Y, Xu J, Pokharel GP, Ellwein LB. Refractive error and visual impairment in urban children in southern China. Invest Ophthalmol Vis Sci. 2004; 45(3): 793-9.

China	Subnational	No	Presenting	blind, moderate, severe	2003	2004	Huang S, Zheng Y, Foster PJ, Huang W, He M. Prevalence and causes of visual impairment in Chinese adults in urban southern China. Arch Ophthalmol. 2009; 127(10): 1362-7.
China	Subnational	Yes	Both	MSVI, blind, moderate, severe	2007	2007	International Centre for Eye Health (ICEH). China - Gao'an Rapid Assessment of Avoidable Blindness 2007. Grootebroek, Netherlands: RAAB Repository.
China	Subnational	Yes	Both	MSVI, blind, moderate, severe	2010	2010	International Centre for Eye Health (ICEH). China - Inner Mongolia Shangdu Rapid Assessment of Avoidable Blindness 2010.
China	Subnational	Yes	Both	MSVI, blind, moderate, severe	2010	2010	International Centre for Eye Health (ICEH). China - Inner Mongolia Tuoketuo Rapid Assessment of Avoidable Blindness 2010.
China	Subnational	Yes	Presenting	MSVI, blind, moderate, severe	2012	2012	International Centre for Eye Health (ICEH). China - Jianchuan Rapid Assessment of Avoidable Blindness 2012. Grootebroek, Netherlands: RAAB Repository.
China	Subnational	Yes	Both	MSVI, blind, moderate, severe	2006	2006	International Centre for Eye Health (ICEH). China - Kunming Rapid Assessment of Avoidable Blindness 2006. Grootebroek, Netherlands: RAAB Repository.
China	Subnational	Yes	Presenting	MSVI, blind, moderate, severe	2012	2012	International Centre for Eye Health (ICEH). China - Lancang Rapid Assessment of Avoidable Blindness 2012. Grootebroek, Netherlands: RAAB Repository.
China	Subnational	Yes	Both	MSVI, blind, moderate, severe	2011	2011	International Centre for Eye Health (ICEH). China - Sichuan Dechang Rapid Assessment of Avoidable Blindness 2011.
China	Subnational	Yes	Both	MSVI, blind,	2017	2018	International Centre for Eye Health (ICEH).

				mild, moderate, severe			China - Sichuan Garzé Rapid Assessment of Avoidable Blindness Survey 2017. Grootebroek, Netherlands: RAAB Repository.[Unpublished].
China	Subnational	Yes	Both	MSVI, blind, moderate, severe	2011	2011	International Centre for Eye Health (ICEH). China - Sichuan Mianning Rapid Assessment of Avoidable Blindness 2011.
China	Subnational	Yes	Both	MSVI, blind, moderate, severe	2007	2007	International Centre for Eye Health (ICEH). China - Wanzai Rapid Assessment of Avoidable Blindness 2007. Grootebroek, Netherlands: RAAB Repository.
China	Subnational	Yes	Both	MSVI, blind, moderate, severe	2007	2007	International Centre for Eye Health (ICEH). China - Xingan Rapid Assessment of Avoidable Blindness 2007. Grootebroek, Netherlands: RAAB Repository.
China	Subnational	Yes	Both	MSVI, blind, mild, moderate, severe	2015	2015	International Centre for Eye Health (ICEH). China - Xinjiang Rapid Assessment of Avoidable Blindness 2015. Grootebroek, Netherlands: RAAB Repository.
China	Subnational	No	Presenting	MSVI, blind	2008	2008	International Centre for Eye Health (ICEH). China - Yunnan Luliang County Rapid Assessment for Avoidable Blindness 2008. Grootebroek, Netherlands: RAAB Repository, 2008.
China	Subnational	Yes	Both	MSVI, blind, moderate, severe	2008	2008	International Centre for Eye Health (ICEH). China - Yunnan Luliang Rapid Assessment of Avoidable Blindness 2008. Grootebroek, Netherlands: RAAB Repository, 2008.
China	Subnational	Yes	Presenting	MSVI, blind, moderate, severe	2009, 2010	2010	Li EY, Liu Y, Zhan X, Liang YB, Zhang X, Zheng C, Jhanji V, Xu P, Chang DF, Lam DS. Prevalence of blindness

							and outcomes of cataract surgery in Hainan Province in South China. Ophthalmology. 2013; 120(11.0): 2176-83.
China	Subnational	No	Presenting	MSVI, blind, moderate , severe	2009, 2010	2010, 2011	Li J, Zhong H, Cai N, Luo T, Li J, Su X, Li X, Qiu X, Yang Y, Yuan Y, Yu M. The prevalence and causes of visual impairment in an elderly Chinese Bai ethnic rural population: the Yunnan minority eye study. Invest Ophthalmol Vis Sci. 2012; 53(8): 4498-504.
China	Subnational	No	Both	MSVI, blind, moderate , severe	1998, 2003	1998, 2003	Li L, Guan H, Xun P, Zhou J, Gu H. Prevalence and causes of visual impairment among the elderly in Nantong, China. Eye (Lond). 2008; 22(8): 1069-75.
China	Subnational	No	Presenting	blind, moderate , severe	1997	1997	Li S, Xu J, He M, Wu K, Munoz SR, Ellwein LB. A survey of blindness and cataract surgery in Doumen County, China. Ophthalmology. 1999; 106(8): 1602-8.
China	Subnational	No	Both	MSVI, blind, moderate , severe	2006	2006	Li T, Du L, Du L. Prevalence and Causes of Visual Impairment and Blindness in Shanxi Province, China. Ophthalmic Epidemiol. 2015; 22(4): 239-45.
China	Subnational	No	Presenting	blind, moderate , severe	2010	2010	Li X, Zhou Q, Sun L, Wang Z, Han S, Wu S, Wang N. Prevalence of blindness and low vision in a rural population in northern China: preliminary results from a population-based survey. Ophthalmic Epidemiol. 2012; 19(5): 272-7.
China	Subnational	No	Both	MSVI, blind, moderate , severe	2008, 2010	2008, 2012	Li Y, Bi HS, Wang LH, Wang T, Yang SY, Liu LP, Zhou CC. Causes of moderate to severe visual impairment and blindness in population aged 50 years or more in rural Shandong province. Chin J Ophthalmol. 2013; 49(2): 144-50.

China	Subnational	No	Presenting	MSVI, blind, moderate, severe	2006	2007	Li Z, Cui H, Liu P, Zhang L, Yang H, Zhang L. Prevalence and causes of blindness and visual impairment among the elderly in rural southern Harbin, China. Ophthalmic Epidemiol. 2008; 15(5): 334-8.
China	Subnational	No	Presenting	blind, moderate, severe	2006	2007	Liang YB, Friedman DS, Wong TY, Zhan SY, Sun LP, Wang JJ, Duan XR, Yang XH, Wang FH, Zhou Q, Wang NL. Prevalence and causes of low vision and blindness in a rural chinese adult population: the Handan Eye Study. Ophthalmology. 2008; 115(11): 1965-72.
China	Subnational	Yes	Both	MSVI, blind, moderate, severe	1986	1986	Lo WB, Fang QX, Zhou JH, Han JJ, Kuang JB, Zhou YF, Yao XL, Tang ZW, Chang Y, Lo B. The epidemiological survey of blindness and low vision in Sichuan Province, China. Eye Sci. 1987; 3(4): 223-6.
China	Subnational	No	Both	blind, moderate, severe	2006	2006	Lu H, Guan HJ, Dai Z, Li M, Wang Y, Hu JY, Shi J, Zhao JL, Ellwein LB, Wang Y, Gao XC. [Prevalence of blindness and moderate and severe visual impairment among adults aged 50 years or above in Qidong City of Jiangsu Province: the China Nine-Province Survey]. Chin J Ophthalmol. 2012; 48(3): 205-10.
China	Subnational	No	Presenting	MSVI, blind, moderate	2004	2004	Lu Q, Zheng Y, Sun B, Cui T, Congdon N, Hu A, Chen J, Shi J. A population-based study of visual impairment among pre-school children in Beijing: the Beijing study of visual impairment in children. Am J Ophthalmol. 2009; 147(6): 1075-81.
China	Subnational	No	Both	MSVI, blind, mild, moderate, severe	2012	2013	Tang Y, Wang X, Wang J, Huang W, Gao Y, Luo Y, Lu Y. Prevalence and Causes of Visual Impairment in a Chinese

							Adult Population: The Taizhou Eye Study. Ophthalmology. 2015; 122(7): 1480-8.
China	Subnational	No	Best-corrected	blind, moderate, severe	2010	2010	Wang G-Q, Bai Z-X, Shi J, Luo S, Chang H-F, Sai X-Y. Prevalence and risk factors for eye diseases, blindness, and low vision in Lhasa, Tibet. Int J Ophthalmol. 2013; 6(2): 237-41.
China	Subnational	No	Presenting	MSVI	2009	2009	Wang H, Zhang Y, Li Z, Wang T, Liu P. Prevalence and causes of corneal blindness. Clin Experiment Ophthalmol. 2013.
China	Subnational	No	Presenting	MSVI, blind	2009	2009	Wang L, Huang W, He M, Zheng Y, Huang S, Liu B, Jin L, Congdon NG, He M. Causes and five-year incidence of blindness and visual impairment in urban Southern China: the Liwan Eye Study. Invest Ophthalmol Vis Sci. 2013; 54(6): 4117-21.
China	Subnational	No	Best-corrected	MSVI, blind, moderate, severe	2000, 2006	2000, 2006	Wei M, Chen H, Fan Y-C, Pathai S. Prevalence and causes of visual impairment and blindness in Sichuan province of China. Int J Ophthalmol. 2010; 3(1): 83-8.
China	Subnational	Yes	Presenting	MSVI, blind, moderate, severe	2006	2006	Wu M, Yip JLY, Kuper H. Rapid assessment of avoidable blindness in Kunming, China. Ophthalmology. 2008; 115(6): 969-74.
China	Subnational	Yes	Both	MSVI, blind, moderate, severe	2007	2007	Xiao B, Kuper H, Guan C, Bailey K, Limburg H. Rapid assessment of avoidable blindness in three counties, Jiangxi Province, China. Br J Ophthalmol. 2010; 94(11): 1437-42.
China	Subnational	No	Both	MSVI, blind, mild, moderate, severe	2001	2001	Xu J, Xu L, Du KF, Shao L, Chen CX, Zhou JQ, Wang YX, You QS, Jonas JB, Wei WB. Subfoveal choroidal thickness in diabetes and diabetic retinopathy [Unpublished data]. Ophthalmology. 2013 Oct;120(10):2023-8.

China	Subnational	No	Presenting	blind, moderate , severe	2006, 2011	2006, 2011	Xu J, Xu L, Du KF, Shao L, Chen CX, Zhou JQ, Wang YX, You QS, Jonas JB, Wei WB. Subfoveal choroidal thickness in diabetes and diabetic retinopathy [Unpublished data]. Ophthalmology. 2013 Oct;120(10):2023-8.
China	Subnational	No	Presenting	blind, moderate , severe	2008	2009	Xu L, Jonas JB, Cui TT, You QS, Wang YX, Yang H, Li JJ, Wei WB, Liang QF, Wang S, Yang XH, Zhang L, Beijing Institute of Ophthalmology, Beijing Tongren Hospital, Capital Medical University. Beijing Eye Public Health Care Project. Ophthalmology. 2012; 119(6): 1167-74.
China	Subnational	No	Both	blind, moderate , severe	2010	2011	Yang M, Zhang JF, Zhu RR, Kang LH, Qin B, Guan HJ. Epidemiological survey of visual impairment in Funing County, Jiangsu. Chin J Ophthalmol. 2017; 53(7): 502-508.
China	Subnational	No	Presenting	MSVI	2008	2009	You QS, Xu L, Wang YX, Liang QF, Cui TT, Yang XH, Yang H, Jonas JB. Prevalence of diabetic retinopathy as cause for visual impairment: the Beijing Public Health Care Project. Clin Experiment Ophthalmol. 2013; 41(6.0): 608-9.
China	Subnational	No	Both	MSVI, blind	2013	2013	Zhang G, Li Y, Teng X, Wu Q, Gong H, Ren F, Guo Y, Liu L, Zhang H. Prevalence and causes of low vision and blindness in Baotou: A cross-sectional study. Medicine (Baltimore). 2016; 95(37): e4905.
China	Subnational	No	Both	MSVI, blind, moderate , severe	1987, 1998	1987, 1998	Zhang SY, Zou LH, Gao YQ, Di Y, Wang XD. National epidemiological survey of blindness and low vision in China. Chin Med J (Engl). 1992; 105(7): 603-8.

China	Subnational	Yes	Presenting	blind, moderate, severe	2012	2012	Zhang X, Li EY, Leung CK, Musch DC, Tang X, Zheng C, He M, Chang DF, Lam DS. Prevalence of visual impairment and outcomes of cataract surgery in Chaonan, South China. PLoS One. 2017; 12(8): e0180769.
China	Subnational	No	Both	MSVI, blind, moderate, severe	2008	2009	Zhang Y, Wang H, Liu J, Wang T, Cao S, Zhou D, Du L, Li Z, Liu P. Prevalence of blindness and low vision: a study in the rural Heilongjiang Province of China. Clin Experiment Ophthalmol. 2012; 40(5): 484-9.
China	Subnational	No	Presenting	blind, moderate, severe	2006	2007	Zhao J, Ellwein LB, Cui H, Ge J, Guan H, Lv J, Ma X, Yin J, Yin ZQ, Yuan Y, Liu H. Prevalence of vision impairment in older adults in rural China: the China Nine-Province Survey. Ophthalmology. 2010; 117(3): 409-16.
China	Subnational	No	Both	MSVI, blind, mild, moderate, severe	2006, 2014	2007, 2014	Zhao J, Xu X, Ellwein LB, Guan H, He M, Liu P, Lv J, Sheng X, Yang P, Yi J, Cai N, Yang M, Chen M, Deng L, Ding X, Du L, Li F, Liu X, Lu H, Shao C, Wang J, Zhuang W, An L. Causes of Visual Impairment and Blindness in the 2006 and 2014 Nine-Province Surveys in Rural China. Am J Ophthalmol. 2019; 197: 80-87.
China	Subnational	No	Presenting	blind, mild, moderate, severe	2009	2009	Zhu M, Tong X, Zhao R, He X, Zhao H, Liu M, Zhu J. Visual impairment and spectacle coverage rate in Baoshan district, China: population-based study. BMC Public Health. 2013; 13(1): 311.
China	Subnational	No	Both	blind, moderate, severe	2010	2011	Zhu RR, Shi J, Yang M, Guan HJ. Prevalences and causes of vision impairment in elderly Chinese: a socioeconomic perspective of a comparative report nested in Jiangsu Eye Study. Int

							J Ophthalmol. 2016; 9(7): 1051-6.
Congo	National	No	Best-corrected	blind, moderate, severe	1988	1988	World Health Organization (WHO). Blindness prevention: prevalence and causes of blindness and visual loss. Wkly Epidemiol Rec. 1990: 65(33): 249-51.
Costa Rica	National	Yes	Both	MSVI, blind, mild, moderate, severe	2015	2015	International Centre for Eye Health (ICEH). Costa Rica Rapid Assessment of Avoidable Blindness Survey 2015. Grootebroek, Netherlands: RAAB Repository, 2018.
Cuba	Subnational	yes	Presenting	MSVI, blind, moderate, severe	2004, 2005	2005	Hernández Silva JR, Río Torres M, Padilla González CM. Resultados del RACSS en Ciudad de La Habana, Cuba, 2005. *Rev Cubana Oftalmol*. 2006; 19(1): 0-0.
Democratic Republic of the Congo	Subnational	Yes	Both	MSVI, blind, moderate, severe	2015	2015	International Centre for Eye Health (ICEH). Democratic Republic of Congo - Orientale Rapid Assessment of Avoidable Blindness 2015. Grootebroek, Netherlands: RAAB Repository.
Denmark	Subnational	No	Both	blind, moderate, severe	1991	1994	Buch H, Vinding T, La Cour M, Appleyard M, Jensen GB, Nielsen NV. Prevalence and causes of visual impairment and blindness among 9980 Scandinavian adults: the Copenhagen City Eye Study. Ophthalmology. 2004; 111(1): 53-61.
Denmark	Subnational	No	Both	MSVI, moderate, severe	1986	1988	Buch H, Vinding T, Nielsen NV. Prevalence and causes of visual impairment according to World Health Organization and United States criteria in an aged, urban Scandinavian population: the Copenhagen City Eye Study. Ophthalmology. 2001; 108(12): 2347-57.
Dominican Republic	National	Yes	Both	MSVI, blind, moderate, severe	2008	2008	International Centre for Eye Health (ICEH). Dominican Republic Rapid Assessment for

							Avoidable Blindness Survey 2008.
Ecuador	National	No	Presenting	MSVI, blind	2009	2009	Ecuador Rapid Assessment for Avoidable Blindness Survey 2009. [Unpublished].
Ecuador	National	Yes	Both	MSVI, blind, moderate, severe	2008	2009	International Centre for Eye Health (ICEH). Ecuador Rapid Assessment of Avoidable Blindness 2008-2009. Grootebroek, Netherlands: RAAB Repository.
Egypt	Subnational	No	Best-corrected	blind, moderate, severe	1994	1994	El-Bayoumy BM, Saad A, Choudhury AH. Prevalence of refractive error and low vision among schoolchildren in Cairo. East Mediterr Health J. 2007; 13(3): 575-9.
Egypt	Subnational	No	Presenting	blind, moderate, severe	2001	2002	Fouad D, Mousa A, Courtright P. Sociodemographic characteristics associated with blindness in a Nile Delta governorate of Egypt. Br J Ophthalmol. 2004; 88(5): 614-8.
Egypt	Subnational	No	Presenting	blind, moderate, severe	2007	2008	Mousa A, Courtright P, Kazanjian A, Bassett K. Prevalence of visual impairment and blindness in Upper Egypt: a gender-based perspective. Ophthalmic Epidemiol. 2014; 21(3.0): 190-6.
El Salvador	National	Yes	Both	MSVI, blind, moderate, severe	2011	2011	International Centre for Eye Health (ICEH). El Salvador Rapid Assessment of Avoidable Blindness 2011.
Eritrea	National	Yes	Both	MSVI, blind, moderate, severe	2008	2008	International Centre for Eye Health (ICEH), Ministry of Health (Eritrea). Eritrea Rapid Assessment for Avoidable Blindness Survey 2008.
Estonia, France, Greece, Italy, Norway, United Kingdom	Subnational	No	Best-corrected	blind, moderate, severe	2006	2007	Seland JH, Vingerling JR, Augood CA, Bentham G, Chakravarthy U, deJong PTVM, Rahu M, Soubrane G, Tomazzoli L, Topouzis F, Fletcher AE. Visual impairment and quality of life in the

							older European population, the EUREYE study. Acta Ophthalmol. 2011; 89(7): 608-13.
Ethiopia	National	No	Presenting	MSVI, blind	2005	2006	Addis Ababa University, Ethiopian Public Health Association, Johns Hopkins University, Ministry of Health (Ethiopia). Ethiopia National Blindness and Low Vision Survey 2005-2006.
Ethiopia	National	No	Presenting	MSVI, blind	2005	2006	Berhane Y, Worku A, Bejiga A, Adamu L, Alemayehu W, Bedri A, Haile Z, Ayalew A, Adamu Y, Gebre T, Kebede TD, West E, West S. Prevalence and causes of blindness and Low Vision in Ethiopia. Ethiop J Health Dev. 2008; 21(3): 204-10.
Ethiopia	Subnational	No	Presenting	blind, moderate , severe	1981	1982	Cerulli L, Cedrone C, Assefa C, Scuderi GL. Assessment of visual status of the population in seven regions of Ethiopia. Rev Int Trach Pathol Ocul Trop Subtrop Sante Publique. 1984; 2-4: 127-42.
Ethiopia	Subnational	No	Both	blind, moderate , severe	2009	2009	Kedir J, Girma A. Prevalence of refractive error and visual impairment among rural school-age children of Goro District, Gurage Zone, Ethiopia. Ethiop J Health Sci. 2014; 24(4): 353-8.
Ethiopia	Subnational	No	Presenting	blind, moderate , severe	2010	2011	Mehari ZA, Yimer AW. Prevalence of refractive errors among schoolchildren in rural central Ethiopia. Clin Exp Optom. 2013; 96(1): 65-9.
Ethiopia	Subnational	No	Presenting	blind, moderate , severe	1998	1998	Melese M, Alemayehu W, Bayu S, Girma T, Hailesellasie T, Khandekar R, Worku A, Courtright P. Low vision and blindness in adults in Gurage Zone, central Ethiopia. Br J Ophthalmol. 2003; 87(6): 677-80.

Ethiopia	Subnational	No	Presenting	MSVI, blind, moderate	1994	1995	Zerihun N, Mabey D. Blindness and low vision in Jimma Zone, Ethopia: results of a population-based survey. Ophthalmic Epidemiol. 1997; 4(1): 19-26.
Fiji	Subnational	No	Presenting	MSVI, blind, moderate, severe	2009	2009	Brian G, Ramke J, Szetu J, Qoqonokana MQ. Cataract and its surgery in Fiji. Clin Experiment Ophthalmol. 2011; 39(5): 449-55.
Fiji	National	No	Best-corrected	blind, moderate, severe	2006	2007	Cama AT, Sikivou BT, Keeffe JE. Childhood visual impairment in Fiji. Arch Ophthalmol. 2010; 128(5): 608-12.
Gambia	National	No	Presenting	blind, moderate, severe	1986, 1996	1986, 1996	Faal H, Minassian D, Sowa S, Foster A. National survey of blindness and low vision in The Gambia: results. Br J Ophthalmol. 1989; 73(2): 82-7.
Gambia	National	No	Presenting	MSVI, blind, moderate, severe	1996	1996	Faal H, Minassian DC, Dolin PJ, Mohamed AA, Ajewole J, Johnson GJ. Evaluation of a national eye care programme: re-survey after 10 years. Br J Ophthalmol. 2000; 84(9): 948-51.
Gambia	National	Yes	Both	MSVI, blind, moderate, severe	2007	2007	International Centre for Eye Health (ICEH). Gambia Rapid Assessment of Avoidable Blindness 2007.
Ghana	Subnational	No	Both	blind, mild, moderate, severe	2006, 2009	2008, 2011	Budenz DL, Bandi JR, Barton K, Nolan W, Herndon L, Whiteside-de Vos J, Hay-Smith G, Kim H, Tielsch J. Blindness and Visual Impairment in an Urban West African Population: The Tema Eye Survey. Ophthalmology. 2012; 119(9): 1744-53.
Guatemala	Subnational	Yes	Both	MSVI, blind, moderate, severe	2004	2004	International Centre for Eye Health (ICEH). Guatemala - Four Departments Rapid Assessment of Avoidable Blindness 2004. Grootebroek, Netherlands: RAAB Repository.
Guatemala	National	Yes	Both	MSVI, blind,	2015	2015	International Centre for Eye Health (ICEH).

				moderate, severe			Guatemala Rapid Assessment of Avoidable Blindness 2015. Grootebroek, Netherlands: RAAB Repository.
Guinea-Bissau	National	Yes	Both	MSVI, blind, moderate, severe	2010	2010	International Centre for Eye Health (ICEH). Guinea-Bissau Rapid Assessment of Avoidable Blindness 2010. Grootebroek, Netherlands: RAAB Repository.
Haiti	Subnational	No	Presenting	moderate, severe	2013	2013	Tousignant B, Brûlé J. Prevalence of eye disease and visual impairment in Île de la Gonave, Haïti. *Med Sante Trop*. 2017; 27(3): 326-328.
Honduras	National	Yes	Presenting	MSVI, blind	2013	2013	Alvarado D, Rivera B, Lagos L, Ochoa M, Starkman I, Castillo M, et al. Encuesta nacional de ceguera y deficiencia visual evitables en Honduras [Honduras national survey of blindness and avoidable visual impairments]. Rev Panam Salud Publica. 2014; 36(5): 300-5.
Honduras	National	Yes	Both	MSVI, blind, moderate, severe	2013	2013	International Centre for Eye Health (ICEH). Honduras Rapid Assessment of Avoidable Blindness 2013.
Hungary	National	Yes	Both	MSVI, blind, mild, moderate, severe	2014	2015	International Centre for Eye Health (ICEH). Hungary Rapid Assessment of Avoidable Blindness 2015. Grootebroek, Netherlands: RAAB Repository.
Iceland	Subnational	No	Both	MSVI, blind, moderate, severe	1996	1996	Gunnlaugsdottir E, Arnarsson A, Jonasson F. Prevalence and causes of visual impairment and blindness in Icelanders aged 50 years and older: the Reykjavik Eye Study. Acta Ophthalmol. 2008; 86(7): 778-85.
India	Subnational	Yes	Presenting	MSVI, blind, moderate, severe	2013	2015	All India Institute of Medical Sciences, New Delhi (AIIMS). India Multi-centric Collaborative Study on the Impact of Global

							Warming and Ultra Violet Radiation Exposure on Ocular Health in India 2013-2015.
India	Subnational	Yes	Presenting	MSVI, blind, moderate, severe	2011	2011	Bettadapura GS, Donthi K, Datti NP, Ranganath BG, Ramaswamy SB, Jayaram TS. Assessment of avoidable blindness using the rapid assessment of avoidable blindness methodology. N Am J Med Sci. 2012; 4(9): 389-93.
India	Subnational	No	Presenting	moderate, severe	1996	1997	Dandona L, Dandona R, Naduvilath TJ, McCarty CA, Srinivas M, Mandal P, Nanda A, Rao GN. Burden of moderate visual impairment in an urban population in southern India. Ophthalmology. 1999; 106(3): 497-504.
India	Subnational	Yes	Presenting	blind, moderate, severe	2009	2009	Dhake PV, Dole K, Khandekar R, Deshpande M. Prevalence and causes of avoidable blindness and severe visual impairment in a tribal district of Maharashtra, India. Oman J Ophthalmol. 2011; 4(3): 129-34.
India	Subnational	Yes	Presenting	blind, moderate, severe	2013	2013	Govekar PK, Sharma KM. Blindness and Visual Impairment in Delhi Region. Natl J Community Med. 5(4): 370-372.
India	Subnational	Yes	Both	MSVI, blind, moderate, severe	2008	2008	International Centre for Eye Health (ICEH). India - Chitrakoot Rapid Assessment of Avoidable Blindness 2008. Grootebroek, Netherlands: RAAB Repository.
India	Subnational	Yes	Presenting	MSVI, blind, moderate, severe	2011	2011	International Centre for Eye Health (ICEH). India - Surat Rapid Assessment of Avoidable Blindness 2011.
India	Subnational	Yes	Presenting	MSVI, blind	2014	2016	Jonas JB, Nangia V, Gupta R, Bhojwani K, Nangia P, Panda-Jonas S. Prevalence of myopic retinopathy in rural Central India. Acta

							Ophthalmol. 2017; 95(5): e399-e404.
India	Subnational	No	Presenting	blind, mild, moderate, severe	1993	1995	Kalikivayi V, Naduvilath TJ, Bansal AK, Dandona L. Visual impairment in school children in southern India. Indian J Ophthalmol. 1997; 45(2): 129-34.
India	Subnational	No	Presenting	blind, moderate, severe	1996	2000	Krishnaiah S, Das T, Nirmalan PK, Nutheti R, Shamanna BR, Rao GN, Thomas R. Risk factors for age-related macular degeneration: findings from the Andhra Pradesh eye disease study in South India. Invest Ophthalmol Vis Sci. 2005; 46(12): 4442-9.
India	Subnational	Yes	Both	MSVI, blind	1995	1995	Limburg H, Kumar R. Follow-up study of blindness attributed to cataract in Karnataka State, India. Ophthalmic Epidemiol. 1998; 5(4): 211-23.
India	Subnational	Yes	Presenting	blind, mild, moderate, severe	2014	2014	Malhotra S, Vashist P, Gupta N, Kalaivani M, Rath R, Gupta SK. Prevalence and causes of visual impairment among adults aged 15-49 years in a rural area of north India - A population-based study. Indian J Ophthalmol. 2018; 66(7): 951-956.
India	Subnational	Yes	Presenting	blind, moderate, severe	2014	2014	Malhotra S, Vashist P, Kalaivani M, Gupta N, Senjam SS, Rath R, Gupta SK. Prevalence and causes of visual impairment amongst older adults in a rural area of North India: a cross-sectional study. BMJ Open. 2018; 8(3): e018894.
India	Subnational	No	Both	MSVI, blind, moderate, severe	1999	2001	Murthy GVS, Gupta SK, Bachani D, Jose R, John N, National Programme for Control of Blindness (India). Current estimates of blindness in India [Unpublished data]. Br J Ophthalmol. 2005; 89(3): 257-60.

India	National	No	Best-corrected	blind, moderate, severe	1999	2001	Murthy GVS, Gupta SK, Bachani D, Jose R, John N. Current estimates of blindness in India. Br J Ophthalmol. 2005; 89(3): 257-60.
India	Subnational	No	Presenting	MSVI, blind	2006	2008	Nangia V, Jonas JB, Kulkarni M, Matin A. Prevalence of age-related macular degeneration in rural central India: the Central India Eye and Medical Study. Retina. 2011; 31(6): 1179-85.
India	Subnational	No	Presenting	blind, moderate, severe	2006	2008	Nangia V, Jonas JB, Sinha A, Bhojwani K, Matin A. Visual impairment among school children in urban Central India: The Central India Children Eye Study. Acta Ophthalmol (Copenh). 2012; 90(4): e329-e331.
India	Subnational	No	Presenting	blind, moderate, severe	2006	2008	Nangia V, Jonas JB, Sinha A, Matin A, Kulkarni M, Panda-Jonas S. Ocular axial length and its associations in an adult population of Central Rural India. The Central India Eye and Medical Study [Unpublished data]. Ophthalmology 2010;117(7):1360-6.
India	Subnational	Yes	Presenting	MSVI, blind, moderate, severe	2007	2007	Neena J, Rachel J, Praveen V, Murthy GVS. Rapid Assessment of Avoidable Blindness in India [Unpublished data]. PLoS One. 2008; 3(8): e2867.
India	National	Yes	Presenting	MSVI, blind	2007	2007	Neena J, Rachel J, Praveen V, Murthy GVS. Rapid Assessment of Avoidable Blindness in India. PLoS One. 2008; 3(8): e2867.
India	Subnational	No	Both	blind, moderate, severe	2010, 2012	2010, 2012	Patil S, Gogate P, Vora S, Ainapure S, Hingane RN, Kulkarni AN, Shammanna BR. Prevalence, causes of blindness, visual impairment and cataract surgical services in Sindhudurg district on the western coastal strip

							of India. Indian J Ophthalmol. 2014; 62(2): 240-245.
India	Subnational	No	Presenting	MSVI, blind	1995	1997	Ramakrishnan R, Nirmalan PK, Krishnadas R, Thulasiraj RD, Tielsch JM, Katz J, Friedman DS, Robin AL. Glaucoma in a rural population of southern India: the Aravind comprehensive eye survey. Ophthalmology. 2003; 110(8): 1484-90.
India	Subnational	No	Best-corrected	blind, moderate, severe	2009	2011	Rustagi N, Uppal Y, Taneja DK. Screening for visual impairment: outcome among schoolchildren in a rural area of Delhi. Indian J Ophthalmol. 2012; 60(3): 203-6.
India	Subnational	Yes	Presenting	blind, moderate, severe	2011	2011, 2013	Singh N, Eeda SS, Gudapati BK, Reddy S, Kanade P, Shantha GPS, Rani PK, Chakrabarti S, Khanna RC. Prevalence and causes of blindness and visual impairment and their associated risk factors, in three tribal areas of Andhra Pradesh, India. PLoS One. 2014; 9(7): e100644.
India	Subnational	No	Both	blind, moderate, severe	1995	1997	Thulasiraj RD, Nirmalan PK, Ramakrishnan R, Krishnadas R, Manimekalai TK, Baburajan NP, Katz J, Tielsch JM, Robin AL. Blindness and vision impairment in a rural south Indian population: the Aravind Comprehensive Eye Survey. Ophthalmology. 2003; 110(8): 1491-8.
India	Subnational	No	Presenting	blind, moderate, severe	2002	2004	Vijaya L, George R, Asokan R, Velumuri L, Ramesh SV. Prevalence and causes of low vision and blindness in an urban population: The Chennai Glaucoma Study. Indian J Ophthalmol. 2014.
Indonesia	Subnational	Yes	Presenting	MSVI, blind, moderate, severe	2006	2006	International Centre for Eye Health (ICEH). Indonesia - East Kalimantan Rapid

							Assessment of Avoidable Blindness 2006.
Indonesia	Subnational	No	Presenting	MSVI, blind, moderate, severe	2001	2002	Saw S-M, Husain R, Gazzard GM, Koh D, Widjaja D, Tan DTH. Causes of low vision and blindness in rural Indonesia. Br J Ophthalmol. 2003; 87(9): 1075-8.
Iran (Islamic Republic of)	Subnational	No	Presenting	blind, moderate, severe	2008	2009	Emamian MH, Zeraati H, Majdzadeh R, Shariati M, Hashemi H, Fotouhi A. The gap of visual impairment between economic groups in Shahroud, Iran: a Blinder-Oaxaca decomposition. Am J Epidemiol. 2011; 173(12): 1463-7.
Iran (Islamic Republic of)	Subnational	No	Best-corrected	blind, moderate, severe	2006	2006	Feghhi M, Khataminia G, Ziaei H, Latifi M. Prevalence and causes of blindness and low vision in Khuzestan province, Iran. J Ophthalmic Vis Res. 2009; 4(1): 29-34.
Iran (Islamic Republic of)	Subnational	No	Presenting	blind, moderate, severe	2002	2002	Fotouhi A, Hashemi H, Mohammad K, Jalali KH. The prevalence and causes of visual impairment in Tehran: the Tehran Eye Study. Br J Ophthalmol. 2004; 88(6): 740-5.
Iran (Islamic Republic of)	Subnational	No	Presenting	blind, moderate, severe	2009	2010	Hashemi H, Khabazkhoob M, Emamian MH, Shariati M, Fotouhi A. Visual impairment in the 40- to 64-year-old population of Shahroud, Iran. Eye (Lond). 2012; 26(8): 1071-7.
Iran (Islamic Republic of)	Subnational	No	Both	blind, moderate, severe	2008	2008	Hashemi H, Khabazkhoob M, Saatchi M, Ostadimoghaddam H, Yekta A. Visual impairment and blindness in a population-based study of Mashhad, Iran. J Curr Ophthalmol. 2018; 30(2): 161-8.
Iran (Islamic Republic of)	Subnational	Yes	Both	MSVI, blind, moderate, severe	2014	2014	International Centre for Eye Health (ICEH). Iran - Kordestan Rapid Assessment of Avoidable Blindness 2014. Grootebroek,

							Netherlands: RAAB Repository.
Iran (Islamic Republic of)	Subnational	Yes	Presenting	MSVI, blind, moderate , severe	2009	2009	International Centre for Eye Health (ICEH). Iran - Varamin Rapid Assessment of Avoidable Blindness 2009. Grootebroek, Netherlands: RAAB Repository.
Iran (Islamic Republic of)	Subnational	Yes	Presenting	blind, moderate , severe	2009	2009	Katibeh M, Behboudi H, Moradian S, Alizadeh Y, Beiranvand R, Sabbaghi H, Ahmadieh H. Rapid Assessment of Avoidable Blindness and Diabetic Retinopathy in Gilan Province, Iran. Ophthalmic Epidemiol. 2017; 24(6): 381-387.
Iran (Islamic Republic of)	Subnational	No	Best-corrected	blind, moderate , severe	2004	2005	Shahriari H-A, Izadi S, Rouhani M-R, Ghasemzadeh F, Maleki A-R. Prevalence and causes of visual impairment and blindness in Sistan-va-Baluchestan Province, Iran: Zahedan Eye Study. Br J Ophthalmol. 2007; 91(5): 579-84.
Israel	Subnational	No	Presenting	blind, moderate , severe	1982	1983	Thomson IM, Chumbley LC. Eye disease in the West Bank and Gaza Strip. Br J Ophthalmol. 1984; 68(8): 598-602.
Italy	Subnational	No	Presenting	MSVI, blind, mild, moderate , severe	2000	2000	Cedrone C, Nucci C, Scuderi G, Ricci F, Cerulli A, Culasso F. Prevalence of blindness and low vision in an Italian population: a comparison with other European studies. Eye (Lond). 2006; 20(6): 661-7.
Italy	Subnational	No	Best-corrected	MSVI, blind, moderate , severe	1988, 2000	1988, 2000	Cedrone C, Ricci F, Nucci C, Cesareo M, MacrÃ¬ G, Culasso F. Age-specific changes in the prevalence of best-corrected visual impairment in an Italian population. Ophthalmic Epidemiol. 2007; 14(5): 320-6.
Jordan	National	Yes	Both	MSVI, blind,	2012	2012	Rabiu MM, Al Bdour MD, Abu Ameerh MA, Jadoon MZ. Prevalence

				moderate, severe			of blindness and diabetic retinopathy in northern Jordan. Eur J Ophthalmol. 2015; 25(4): 320-7.
Kenya	Subnational	Yes	Presenting	MSVI, blind, moderate, severe	2007	2007	International Centre for Eye Health (ICEH). Kenya - Embu Rapid Assessment of Avoidable Blindness 2007. Grootebroek, Netherlands: RAAB Repository.
Kenya	Subnational	Yes	Both	MSVI, blind, moderate, severe	2007	2007	International Centre for Eye Health (ICEH). Kenya - Kericho Rapid Assessment of Avoidable Blindness 2007. Grootebroek, Netherlands: RAAB Repository.
Kenya	Subnational	Yes	Both	MSVI, blind, moderate, severe	2011	2011	International Centre for Eye Health (ICEH). Kenya - Kwale Rapid Assessment of Avoidable Blindness 2011. Grootebroek, Netherlands: RAAB Repository.
Kenya	Subnational	Yes	Both	MSVI, blind, moderate, severe	2005	2005	International Centre for Eye Health (ICEH). Kenya - Nakuru Rapid Assessment of Avoidable Blindness 2005. Grootebroek, Netherlands: RAAB Repository.
Kenya	Subnational	No	Presenting	MSVI, blind, moderate, severe	2002	2003	Ndegwa LK, Karimurio J, Okelo RO, Adala HS. Prevalence of visual impairment and blindness in a Nairobi urban population. East Afr Med J. 2006; 83(4): 69-72.
Kenya	National	No	Presenting	MSVI, blind	1976	1981	Schwab L, Steinkuller PG. Visual disability and blindness secondary to refractive errors in Africa. Soc Sci Med. 1983; 17(22): 1751-4.
Kenya	National	No	Presenting	MSVI, blind	1987	1988	Whitfield R, Schwab L, Ross-Degnan D, Steinkuller P, Swartwood J. Blindness and eye disease in Kenya: ocular status survey results from the Kenya Rural Blindness Prevention Project. Br J Ophthalmol. 1990; 74(6): 333-40.

Kyrgyzstan	Subnational	Yes	Both	MSVI, blind, mild, moderate, severe	2017	2017	International Centre for Eye Health (ICEH). Kyrgyzstan - Batken, Jalal Abad, and Osh Rapid Assessment of Avoidable Blindness Survey 2017. Grootebroek, Netherlands: RAAB Repository, 2017.
Lao People's Democratic Republic	Subnational	No	Presenting	blind, moderate, severe	2009	2009	Casson RJ, Kahawita S, Kong A, Muecke J, Sisaleumsak S, Visonnavong V. Exceptionally low prevalence of refractive error and visual impairment in schoolchildren from Lao People's Democratic Republic. Ophthalmology. 2012; 119(10): 2021-7.
Lao People's Democratic Republic	National	Yes	Both	MSVI, blind, moderate, severe	2007	2007	International Centre for Eye Health (ICEH). Laos Rapid Assessment of Avoidable Blindness Survey 2007. Grootebroek, Netherlands: RAAB Repository, 2017.
Lebanon	National	No	Presenting	blind, moderate, severe	1995	1995	Mansour AM, Kassak K, Chaya M, Hourani T, Sibai A, Alameddine MN. National survey of blindness and low vision in Lebanon. Br J Ophthalmol. 1997; 81(10): 905-6.
Libya	National	No	Presenting	MSVI, blind, moderate, severe	2010	2010	Rabiu MM, Jenf M, Fituri S, Choudhury A, Agbabiaka I, Mousa A. Prevalence and causes of visual impairment and blindness, cataract surgical coverage and outcomes of cataract surgery in Libya. Ophthalmic Epidemiol. 2013; 20(1): 26-32.
Madagascar	Subnational	Yes	Both	MSVI, blind, moderate, severe	2011	2011	International Centre for Eye Health (ICEH). Madagascar - Atsinanana Rapid Assessment of Avoidable Blindness 2011. Grootebroek, Netherlands: RAAB Repository.
Madagascar	Subnational	No	Presenting	MSVI, blind	2010	2010	Randrianaivo JB, Anholt RM, Tendrisoa DL,

							Margiano NJ, Courtright P3, Lewallen S. Blindness and cataract surgical services in Atsinanana region, Madagascar. Middle East Afr J Ophthalmol. 2014; 21(2): 153-7.
Malawi	Subnational	No	Both	blind, moderate, severe	1983	1983	Chirambo MC, Tielsch JM, West KP Jr, Katz J, Tizazu T, Schwab L, Johnson G, Swartwood J, Taylor HR, Sommer A. Blindness and visual impairment in southern Malawi. Bull World Health Organ. 1986; 64(4): 567-72.
Malawi	Subnational	Yes	Both	MSVI, blind, moderate, severe	2009	2010	International Centre for Eye Health (ICEH). Malawi - Southern Region Rapid Assessment of Avoidable Blindness 2009-2010. Grootebroek, Netherlands: RAAB Repository.
Malawi	Subnational	Yes	Presenting	blind, moderate, severe	2009	2010	Kalua K, Lindfield R, Mtupanyama M, Mtumodzi D, Msiska V. Findings from a rapid assessment of avoidable blindness (RAAB) in Southern Malawi. PLoS One. 2011; 6(4): e19226.
Malaysia	Subnational	No	Presenting	blind, mild, moderate, severe	2003	2003	Goh P-P, Abqariyah Y, Pokharel GP, Ellwein LB. Refractive error and visual impairment in school-age children in Gombak District, Malaysia. Ophthalmology. 2005; 112(4): 678-85.
Malaysia	Subnational	Yes	Both	MSVI, blind, mild, moderate, severe	2014	2014	International Centre for Eye Health (ICEH). Malaysia - Kelantan, Terengganu, and Pahang Rapid Assessment of Avoidable Blindness Survey 2014. Grootebroek, Netherlands: RAAB Repository, 2014.
Malaysia	Subnational	Yes	Both	MSVI, blind, mild, moderate, severe	2014	2014	International Centre for Eye Health (ICEH). Malaysia - Sabah Rapid Assessment of Avoidable Blindness Survey 2014. Grootebroek,

							Netherlands: RAAB Repository, 2014.
Malaysia	Subnational	Yes	Both	MSVI, blind, mild, moderate, severe	2014	2014	International Centre for Eye Health (ICEH). Malaysia - Sarawak Rapid Assessment of Avoidable Blindness Survey 2014. Grootebroek, Netherlands: RAAB Repository, 2014.
Malaysia	Subnational	No	Presenting	blind, moderate, severe	2000	2000	Reddy SC, Rampal L, Nurulaini O. Prevalence and causes of visual impairment and blindness in a rural population in Sepang district, Selangor. Med J Malaysia. 2004; 59(2): 212-7.
Malaysia	National	No	Presenting	MSVI, blind, moderate, severe	1996	1997	Zainal M, Ismail SM, Ropilah AR, Elias H, Arumugam G, Alias D, Fathilah J, Lim TO, Ding LM, Goh PP. Prevalence of blindness and low vision in Malaysian population: results from the National Eye Survey 1996. Br J Ophthalmol. 2002; 86(9): 951-6.
Malaysia	Subnational	No	Both	MSVI, blind, moderate, severe	1993	1994	Zainal M, Masran L, Ropilah AR. Blindness and visual impairment amongst rural Malays in Kuala Selangor, Selangor. Med J Malaysia. 1998; 53(1): 46-50.
Maldives	National	Yes	Both	MSVI, blind, moderate, severe	2016	2016	International Centre for Eye Health (ICEH). Maldives Rapid Assessment of Avoidable Blindness 2016. Grootebroek, Netherlands: RAAB Repository.
Mali	Subnational	Yes	Both	MSVI, blind, moderate, severe	2008	2008	International Centre for Eye Health (ICEH). Mali - Koulikoro Rapid Assessment of Avoidable Blindness 2008. Grootebroek, Netherlands: RAAB Repository.
Mali	Subnational	Yes	Both	MSVI, blind, moderate, severe	2011	2011	International Centre for Eye Health (ICEH). Mali - Koulikoro Rapid Assessment of Avoidable Blindness 2011. Grootebroek,

							Netherlands: RAAB Repository.
Mali	Subnational	No	Both	MSVI, blind	1990	1990	Kortlang C, Koster JC, Coulibaly S, Dubbeldam RP. Prevalence of blindness and visual impairment in the region of Ségou, Mali. A baseline survey for a primary eye care programme. *Trop Med Int Health*. 1996; 1(3): 314-9.
Mexico	Subnational	Yes	Both	MSVI, blind, moderate, severe	2009	2010	International Centre for Eye Health (ICEH), Tennent Institute of Ophthalmology, Vision Institute, Carlota Hospital (Nuevo LeÃ³n). Mexico - Chiapas Rapid Assessment of Avoidable Blindness and Diabetic Retinopathy 2010.
Mexico	Subnational	Yes	Both	MSVI, blind, moderate, severe	2005	2006	International Centre for Eye Health (ICEH). Mexico - Nuevo Leon Rapid Assessment of Avoidable Blindness 2005-2006. Grootebroek, Netherlands: RAAB Repository.
Mexico	Subnational	Yes	Both	MSVI, blind, mild, moderate, severe	2014	2014	International Centre for Eye Health (ICEH). Mexico - Nuevo Leon Rapid Assessment of Avoidable Blindness Survey 2014. Grootebroek, Netherlands: RAAB Repository, 2014.
Mexico	Subnational	Yes	Both	MSVI, blind, mild, moderate, severe	2015	2016	International Centre for Eye Health (ICEH). Mexico - Querétaro de Arteaga Rapid Assessment of Avoidable Blindness 2015-2016. Grootebroek, Netherlands: RAAB Repository.
Mexico	Subnational	No	Presenting	moderate, severe	2008	2008	Secretariat of Health (Mexico), Secretary of Public Education (Mexico). Mexico National School Health Survey 2008. Cuernavaca, Mexico: National Institute of Public Health (Mexico), 2010.

Mongolia	National	Yes	Both	MSVI, blind, moderate, severe	2012	2013	International Centre for Eye Health (ICEH). Mongolia Rapid Assessment of Avoidable Blindness Survey 2013. Grootebroek, Netherlands: RAAB Repository, 2014.
Morocco	National	No	Both	MSVI, blind, moderate, severe	1992	1992	World Health Organization (WHO). Prevention of blindness (PBL). Prevalence and causes of blindness and low vision.Wkly Epidemiol Rec. 1994: 69(18): 129-31.
Mozambique	Subnational	Yes	Both	MSVI, blind, moderate, severe	2011	2011	International Centre for Eye Health (ICEH). Mozambique - Nampula Rapid Assessment of Avoidable Blindness Survey 2011. Grootebroek, Netherlands: RAAB Repository, 2014.
Mozambique	Subnational	Yes	Both	MSVI, blind, moderate, severe	2009, 2012	2010, 2012	International Centre for Eye Health (ICEH). Mozambique - Sofala Division Rapid Assessment of Avoidable Blindness 2012. Grootebroek, Netherlands: RAAB Repository.
Myanmar	Subnational	No	Both	MSVI, blind, moderate, severe	2005	2005	Casson RJ, Newland HS, Muecke J, McGovern S, Durkin S, Sullivan T, Oo TZ, Aung TH, Shein WK, Selva D, Aung T. Prevalence and causes of visual impairment in rural Myanmar: the Meiktila Eye Study. Ophthalmology. 2007; 114(12): 2302-8.
Nepal	Subnational	No	Presenting	blind, moderate, severe	2009	2011	Dev MK, Shrestha GS, Paudel N, Joshi ND, Thapa M, Shah DN. Visual status and ocular morbidity in older adults living in residential care. Graefes Arch Clin Exp Ophthalmol. 2012; 250(9): 1387-93.
Nepal	Subnational	No	Presenting	MSVI, blind, moderate, severe	2009	2011	Dulal S, Sapkota YD. Prevalence of blindness and visual impairment and its causes among people aged 50 years and above in Karnali Zone,

							Nepal. Nepal J Ophthalmol. 2012; 4(2).
Nepal	Subnational	Yes	Both	MSVI, blind, moderate, severe	2008	2008	International Centre for Eye Health (ICEH), Tilganga Institute of Ophthalmology. Nepal - Bagmati Zone Rapid Assessment for Avoidable Blindness 2008.
Nepal	Subnational	Yes	Both	MSVI, blind, moderate, severe	2008	2008	International Centre for Eye Health (ICEH). Nepal - Bheri Zone Rapid Assessment of Avoidable Blindness 2009.
Nepal	Subnational	Yes	Both	MSVI, blind, moderate, severe	2010	2010	International Centre for Eye Health (ICEH). Nepal - Dhaulagiri Zone Rapid Assessment of Avoidable Blindness 2010.
Nepal	Subnational	Yes	Both	MSVI, blind, moderate, severe	2008	2008	International Centre for Eye Health (ICEH). Nepal - Janakpur Rapid Assessment for Avoidable Blindness 2008. Grootebroek, Netherlands: RAAB Repository.
Nepal	Subnational	Yes	Both	MSVI, blind, moderate, severe	2008	2008	International Centre for Eye Health (ICEH). Nepal - Karnali Zone Rapid Assessment of Avoidable Blindness 2008.
Nepal	Subnational	Yes	Both	MSVI, blind, moderate, severe	2008	2009	International Centre for Eye Health (ICEH). Nepal - Koshi Zone Rapid Assessment of Avoidable Blindness 2008-2009.
Nepal	Subnational	Yes	Both	MSVI, blind, moderate, severe	2009	2009	International Centre for Eye Health (ICEH). Nepal - Mechi Zone Rapid Assessment of Avoidable Blindness 2009.
Nepal	Subnational	Yes	Both	MSVI, blind, moderate, severe	2015	2016	International Centre for Eye Health (ICEH). Nepal - Narayani Rapid Assessment of Avoidable Blindness 2015-2016. Grootebroek, Netherlands: RAAB Repository.
Nepal	Subnational	Yes	Both	MSVI, blind, moderate, severe	2010	2010	International Centre for Eye Health (ICEH). Nepal - Rapti Zone Rapid Assessment of Avoidable Blindness 2010.

Nepal	Subnational	Yes	Both	MSVI, blind, moderate, severe	2008	2009	International Centre for Eye Health (ICEH). Nepal - Sagarmatha Zone Rapid Assessment of Avoidable Blindness 2008-2009.
Nepal	Subnational	Yes	Both	MSVI, blind, moderate, severe	2008	2008	International Centre for Eye Health (ICEH). Nepal - Seti and Mahakali Zones Rapid Assessment of Avoidable Blindness 2008.
Nepal	Subnational	No	Presenting	blind, moderate, severe	1997	1997	Pokharel GP, Negrel AD, Munoz SR, Ellwein LB. Refractive Error Study in Children: results from Mechi Zone, Nepal. Am J Ophthalmol. 2000; 129(4): 436-44.
Nepal	Subnational	Yes	Presenting	blind, moderate, severe	2006	2006	Sapkota YD, Sunuwar M, Naito T, Akura J, Adhikari HK. The prevalence of blindness and cataract surgery in Rautahat District, Nepal. Ophthalmic Epidemiol. 2010; 17(2): 82-9.
Nepal	Subnational	Yes	Presenting	blind, moderate, severe	2006	2006	Sherchan A, Kandel RP, Sharma MK, Sapkota YD, Aghajanian J, Bassett KL. Blindness prevalence and cataract surgical coverage in Lumbini Zone and Chetwan District of Nepal. Br J Ophthalmol. 2010; 94(2): 161-6.
Nepal	Subnational	No	Best-corrected	moderate, severe	2007	2007	Sherpa D, Panta CR, Joshi N. Ocular morbidity among primary school children of Dhulikhel, Nepal. Nepal J Ophthalmol. 2011; 3(2): 172-6.
Nepal	Subnational	No	Both	MSVI, blind, moderate, severe	2013	2015	Thapa R, Bajimaya S, Paudyal G, Khanal S, Tan S, Thapa SS, van Rens GHMB. Prevalence and causes of low vision and blindness in an elderly population in Nepal: the Bhaktapur retina study. BMC Ophthalmol. 2018; 18(1): 42.
Nepal	Subnational	No	Presenting	MSVI, blind, moderate, severe	2008	2009	Thapa SS, Berg RVD, Khanal S, Paudyal I, Pandey P, Maharjan N, Twyana SN, Paudyal G, Gurung R, Ruit S, Rens

							GHMBV. Prevalence of visual impairment, cataract surgery and awareness of cataract and glaucoma in Bhaktapur district of Nepal: the Bhaktapur Glaucoma Study. BMC Ophthalmol. 2011; 11: 2.
Nepal	Subnational	No	Presenting	MSVI, blind	2009	2011	Thapa SS, Paudyal I, Khanal S, Twyana SN, Paudyal G, Gurung R, Ruit S, van Rens GHMB. A population-based survey of the prevalence and types of glaucoma in Nepal: the Bhaktapur Glaucoma Study. Ophthalmology. 2012; 119(4): 759-64.
Nepal	Subnational	No	Both	MSVI, blind	2012	2012	Thapa SS, Poudyal I, Khanal S, van Rens G. Results of the Bhaktapur Glaucoma Study, Nepal. Nepal J Ophthalmol. 2013; 5(1.0): 81-93.
Netherlands	Subnational	No	Presenting	blind, moderate, severe	1997	1999	Gussekloo J, de Craen AJM, Oduber C, van Boxtel MPJ, Westendorp RGJ. Sensory impairment and cognitive functioning in oldest-old subjects: the Leiden 85+ Study. Am J Geriatr Psychiatry. 2005; 13(9): 781-6.
Netherlands	Subnational	No	Both	MSVI, blind, mild, moderate, severe	1990	1993	Klaver CC, Wolfs RC, Vingerling JR, Hofman A, de Jong PT. Age-specific prevalence and causes of blindness and visual impairment in an older population: the Rotterdam Study. Arch Ophthalmol. 1998; 116(5): 653-8.
Nigeria	Subnational	No	Presenting	MSVI, blind, moderate, severe	1995	1995	Abdu L. Prevalence and causes of blindness and low vision in Dambatta local government area, Kano State, Nigeria. Niger J Med. 2002; 11(3): 108-12.
Nigeria	Subnational	No	Best-corrected	blind, moderate, severe	2010	2012	Abegunde KA, Owoaje ET. Health problems and associated risk factors in selected urban and rural elderly population groups of South-West Nigeria. Ann Afr Med. 2013; 12(2): 90-7.

Nigeria	Subnational	No	Presenting	blind, moderate, severe	2002	2002	Ajaiyeoba AI, Isawumi MA, Adeoye AO, Oluleye TS. Pattern of eye diseases and visual impairment among students in southwestern Nigeria. Int J Ophthalmol. 2007; 27(5): 287-92.
Nigeria	Subnational	No	Best-corrected	blind, moderate, severe	2007	2007	Ejimadu CS, Adio AO. The burden of low vision in farming communities in South-South Nigeria. Niger J Med. 2012; 21(2): 218-22.
Nigeria	Subnational	No	Both	MSVI, blind, moderate, severe	1992	1992	Ezepue UF. Magnitude and causes of blindness and low vision in Anambra State of Nigeria (results of 1992 point prevalence survey). Public Health. 1997; 111(5): 305-9.
Nigeria	Subnational	Yes	Both	MSVI, blind, mild, moderate, severe	2016	2016	International Centre for Eye Health (ICEH). Nigeria - Sokoto Rapid Assessment of Avoidable Blindness 2016. Grootebroek, Netherlands: RAAB Repository.
Nigeria	Subnational	Yes	Presenting	MSVI, blind, moderate, severe	2005	2005	Kolawole OU, Ashaye AO, Mahmoud AO, Adeoti CO. Cataract blindness in Osun state, Nigeria: results of a survey. Middle East Afr J Ophthalmol. 2012; 19(4): 364-71.
Nigeria	Subnational	No	Presenting	MSVI, blind	2006	2007	Komolafe OO, Ashaye AO, Ajayi BGK, Bekibele CO. Visual impairment from age-related cataract among an indigenous African population. Eye (Lond). 2010; 24(1): 53-8.
Nigeria	National	No	Presenting	MSVI, blind, mild, moderate, severe	2005, 2006, 2007	2007	Kyari F, Gudlavalleti MVS, Sivsubramaniam S, Gilbert CE, Abdull MM, Entekume G, Foster A. Prevalence of blindness and visual impairment in Nigeria: the National Blindness and Visual Impairment Study. Invest Ophthalmol Vis Sci. 2009; 50(5): 2033-9.

Nigeria	Subnational	No	Presenting	blind, moderate, severe	2005	2005	Muhammad N, Mansur RM, Dantani AM, Elhassan E, Isiyaku S. Prevalence and causes of blindness and visual impairment in Sokoto State, Nigeria: baseline data for vision 2020: the Right to Sight Eye Care Programme. Middle East Afr J Ophthalmol. 2011; 18(2): 123-8.
Nigeria	Subnational	Yes	Presenting	MSVI, blind	2010	2012	Okoye O, Umeh RE, Ezepue FU. Prevalence of eye diseases among school children in a rural south-eastern Nigerian community. Rural Remote Health. 2013; 13(3): 2357.
Nigeria	Subnational	No	Presenting	MSVI, blind, moderate	2004	2005, 2006	Onakpoya OH, Adeoye AO, Akinsola FB, Adegbehingbe BO. Prevalence of blindness and visual impairment in Atakunmosa West Local Government area of southwestern Nigeria. Tanzan Health Res Bull. 2007; 9(2): 126-31.
Nigeria	Subnational	Yes	Presenting	blind, moderate, severe	1999	1999	Rabiu MM. Cataract blindness and barriers to uptake of cataract surgery in a rural community of northern Nigeria. Br J Ophthalmol. 2001; 85(7): 776-80.
Oman	National	No	Presenting	blind, moderate, severe	2005	2005	Khandekar R, Mohammed AJ, Raisi AA. Prevalence and causes of blindness and low vision: before and five years after "VISION 2020" initiatives in Oman: a review. Ophthalmic Epidemiol. 2007; 14(1): 9-15.
Pakistan	Subnational	No	Presenting	MSVI, blind, moderate, severe	1998	1998	Ahmad K, Khan MD, Qureshi MB, Munami S, Shah RA, Rasheed H, Jamali B, Baluch A, Khan MA. Prevalence and causes of blindness and low vision in a rural setting in Pakistan. Ophthalmic Epidemiol. 2005; 12(1): 19-23.
Pakistan	Subnational	Yes	Presenting	blind, moderate, severe	2000	2001	Haider S, Hussain A, Limburg H. Cataract blindness in Chakwal

							District, Pakistan: results of a survey. Ophthalmic Epidemiol. 2003; 10(4): 249-58.
Pakistan	Subnational	Yes	Presenting	MSVI, blind, moderate , severe	2000	2000	International Centre for Eye Health (ICEH). Pakistan - Chakwal Rapid Assessment of Avoidable Blindness 2000.
Pakistan	Subnational	Yes	Both	MSVI, blind, moderate , severe	2013	2013	International Centre for Eye Health (ICEH). Pakistan - Peshawar Rapid Assessment of Avoidable Blindness 2013. Grootebroek, Netherlands: RAAB Repository.
Pakistan	National	No	Presenting	MSVI, blind, mild, moderate , severe	2001	2003	Pakistan National Survey on Blindness and Low Vision 2002-2004. [Unpublished].
Pakistan	Subnational	No	Presenting	blind, moderate , severe	2003	2003	Shaikh SP, Aziz TM. Pattern of eye diseases in children of 5-15 years at Bazzertaline Area (South Karachi) Pakistan. J Coll Physicians Surg Pak. 2005; 15(5): 291-4.
Palestine	National	Yes	Presenting	MSVI, blind, moderate , severe	2008	2008	Chiang F, Kuper H, Lindfield R, Keenan T, Seyam N, Magauran D, Khalilia N, Batta H, Abdeen Z, Sargent N. Rapid assessment of avoidable blindness in the Occupied Palestinian Territories. PLoS One. 2010; 5(7): e11854.
Palestine	National	Yes	Both	MSVI, blind, moderate , severe	2008	2008	International Centre for Eye Health (ICEH). Palestine Rapid Assessment of Avoidable Blindness 2008. Grootebroek, Netherlands: RAAB Repository.
Panama	National	Yes	Both	MSVI, blind, moderate , severe	2012	2014	International Centre for Eye Health (ICEH), Ophthalmology School, Specialized University of the Americas (Panama). Panama Rapid Assessment of Avoidable Blindness 2012-2014. Grootebroek, Netherlands: RAAB Repository.

Papua New Guinea	Subnational	Yes	Presenting	MSVI, blind, moderate, severe	2004	2005	Garap JN, Sheeladevi S, Shamanna BR, Nirmalan PK, Brian G, Williams C. Blindness and vision impairment in the elderly of Papua New Guinea. Clin Experiment Ophthalmol. 2006; 34(4): 335-41.
Paraguay	National	Yes	Presenting	MSVI, blind, moderate, severe	1999	1999	Duerksen R, Limburg H, Carron JE, Foster A. Cataract blindness in Paraguay – results of a national survey. *Ophthalmic Epidemiol*. 2003; 10(5): 349-57.
Paraguay	National	Yes	Presenting	MSVI, blind, moderate, severe	1999, 2011	1999, 2011	Duerksen R, Limburg H, Lansingh VC, Silva JC. Review of blindness and visual impairment in Paraguay: changes between 1999 and 2011. Ophthalmic Epidemiol. 2013; 20(5.0): 301-7.
Paraguay	National	Yes	Both	MSVI, blind, moderate, severe	2011	2011	International Centre for Eye Health (ICEH). Paraguay Rapid Assessment of Avoidable Blindness 2011.
Peru	National	Yes	Both	MSVI, blind, moderate, severe	2011	2012	International Centre for Eye Health (ICEH). Peru Rapid Assessment of Avoidable Blindness 2011-2012. Grootebroek, Netherlands: RAAB Repository.
Peru	Subnational	Yes	Both	MSVI, blind, moderate, severe	2002	2003	Pongo Aguila L, Carrión R, Luna W, Silva JC, Limburg H. [Cataract blindness in people 50 years old or older in a semirural area of northern Peru]. *Rev Panam Salud Publica*. 2005; 17(5-6): 387-93.
Philippines	Subnational	Yes	Presenting	MSVI, blind, moderate, severe	2005, 2006	2005, 2006	Eusebio C, Kuper H, Polack S, Enconado J, Tongson N, Dionio D, Dumdum A, Limburg H, Foster A. Rapid assessment of avoidable blindness in Negros Island and Antique District, Philippines. Br J Ophthalmol. 2007; 91(12): 1588-92.
Philippines	Subnational	Yes	Both	MSVI, blind,	2006	2006	International Centre for Eye Health (ICEH). Philippines - Antique

				moderate, severe			Rapid Assessment of Avoidable Blindness 2006. Grootebroek, Netherlands: RAAB Repository.
Philippines	Subnational	Yes	Both	MSVI, blind, moderate, severe	2005	2005	International Centre for Eye Health (ICEH). Philippines - Negros Island Rapid Assessment of Avoidable Blindness 2005. Grootebroek, Netherlands: RAAB Repository.
Qatar	National	Yes	Both	blind, moderate, severe	2009	2009	Al Gamra H, Al Mansouri F, Khandekar R, Elshafei M, Al Qahtani O, Singh R, Hashim SP, Mujahed A, Makled A, Pai A. Prevalence and causes of blindness, low vision and status of cataract in 50 years and older citizen of Qatar – a community based survey. *Ophthalmic Epidemiol*. 2010; 17(5): 292-300.
Republic of Moldova	National	Yes	Both	MSVI, blind, moderate, severe	2012	2012	Zatic T1, Bendelic E2, Paduca A2, Rabiu M3, Corduneanu A2, Garaba A, Novac V, Curca C, Sorbala I, Chiaburu A, Verega F, Andronic V, Guzun I, Căpăţînă O, Zamă-Mardari I. Rapid assessment of avoidable blindness and diabetic retinopathy in Republic of Moldova. *Br J Ophthalmol*. 2015; 99(6): 832-6.
Russian Federation	Subnational	Yes	Both	MSVI, blind, moderate, severe	2008	2009	International Centre for Eye Health (ICEH). Russia - Samara District Rapid Assessment for Avoidable Blindness Survey 2008.
Russian Federation	Subnational	No	Presenting	blind, moderate, near vision loss, severe	2015	2017	Ufa Eye Research Institute. Russia - Ural Eye and Medical Study 2015-2017.
Rwanda	National	Yes	Both	MSVI, blind, moderate, severe	2015	2015	International Centre for Eye Health (ICEH). Rwanda Rapid Assessment of Avoidable Blindness Survey 2015. Grootebroek,

							Netherlands: RAAB Repository, 2014.
Rwanda	Subnational	Yes	Both	MSVI, blind, moderate, severe	2006	2006	Mathenge W NJ, Limburg H, Kuper H. Rapid assessment of avoidable blindness in Western Rwanda: blindness in a postconflict setting. PLoS Med. 2007; 4(7).
Saudi Arabia	Subnational	Yes	Presenting	MSVI, blind, moderate, severe	2011	2011	Al Ghamdi AH, Rabiu M, Hajar S, Yorston D, Kuper H, Polack S. Rapid assessment of avoidable blindness and diabetic retinopathy in Taif, Saudi Arabia. Br J Ophthalmol. 2012; 96(9): 1168-72.
Saudi Arabia	Subnational	No	Best-corrected	blind, moderate, severe	1984, 1989	1984, 1990	Badr IA, Saif AM, Al-Rajhi AA. Changing patterns of visual loss in the Eastern Province, Kingdom of Saudi Arabia. Saudi J Ophthalmol. 2004; 18: SI56-SI64.
Senegal	Subnational	Yes	Both	MSVI, blind, moderate, severe	2010	2010	International Centre for Eye Health (ICEH). Senegal - Fatick Rapid Assessment of Avoidable Blindness 2010. Grootebroek, Netherlands: RAAB Repository.
Senegal	Subnational	Yes	Both	MSVI, blind, moderate, severe	2010	2010	International Centre for Eye Health (ICEH). Senegal - Kaolack Rapid Assessment of Avoidable Blindness 2010. Grootebroek, Netherlands: RAAB Repository.
Sierra Leone	National	Yes	Both	MSVI, blind, moderate, severe	2010	2011	International Centre for Eye Health (ICEH). Sierra Leone Rapid Assessment of Avoidable Blindness 2010-2011.
Singapore	Subnational	No	Presenting	blind, moderate, severe	2007	2008	Dirani M, Zhou B, Hornbeak D, Chang BC, Gazzard G, Chia A, Ling Y, Selvaraj P, Young TL, Varma R, Wong TY, Saw SM. Prevalence and causes of decreased visual acuity in Singaporean Chinese preschoolers. Br J Ophthalmol. 2010; 94(12): 1561-5.

Singapore	Subnational	No	Both	MSVI, blind, moderate, severe	1997	1998	Saw S-M, Foster PJ, Gazzard G, Seah S. Causes of blindness, low vision, and questionnaire-assessed poor visual function in Singaporean Chinese adults: The Tanjong Pagar Survey. Ophthalmology. 2004; 111(6): 1161-8.
Singapore	Subnational	No	Presenting	MSVI, blind, moderate, severe	2004	2006	Wong TY, Chong EW, Wong W-L, Rosman M, Aung T, Loo J-L, Shen S, Loon S-C, Tan DTH, Tai ES, Saw S-M. Prevalence and causes of low vision and blindness in an urban malay population: the Singapore Malay Eye Study. Arch Ophthalmol. 2008; 126(8): 1091-9.
South Africa	Subnational	Yes	Both	MSVI, blind, moderate, severe	2010	2010	Cockburn N, Steven D, Lecuona K, Joubert F, Rogers G, Cook C, Polack S. Prevalence, Causes and Socio-Economic Determinants of Vision Loss in Cape Town, South Africa. Atashili J, editor. PLoS One. 2012; 7(2): e30718.
South Africa	Subnational	No	Presenting	blind, moderate, severe	1990	1991	Cook CD, Knight SE, Crofton-Briggs I. Prevalence and causes of low vision and blindness in northern KwaZulu. S Afr Med J. 1993; 83(8): 590-3.
South Africa	Subnational	Yes	Presenting	MSVI, blind, moderate, severe	2009	2009	Govender P, Ramson P, Visser L, Naidoo KS. Rapid assessment of avoidable blindness in the northern eThekwini district of KwaZulu-Natal Province, South Africa. Afr Vis Eye Health. 2015; 74(1): 1-7.
South Africa	Subnational	Yes	Both	MSVI, blind, moderate, severe	2010	2010	International Centre for Eye Health (ICEH). South Africa Rapid Assessment of Avoidable Blindness Survey 2010. Grootebroek, Netherlands: RAAB Repository, 2014.
South Sudan	Subnational	No	Presenting	MSVI, blind, moderate, severe	2003, 2005	2005	Ngondi J, Ole-Sempele F, Onsarigo A, Matende I, Baba S, Reacher M, Matthews F, Brayne C, Emerson PM. Prevalence

							and causes of blindness and low vision in southern Sudan. PLoS Med. 2006; 3(12): e477.
Spain	Subnational	No	Best-corrected	MSVI, blind, mild	2007	2007	Antón A, Andrada MT, Mayo A, Portela J, Merayo J. Epidemiology of refractive errors in an adult European population: the Segovia study. *Ophthalmic Epidemiol*. 2009; 16(4): 231-7.
Spain	National	No	Presenting	blind, moderate, severe	2011	2012	Garin N, Olaya B, Lara E, Moneta MV, Miret M, Ayuso-Mateos JL, Haro JM. Visual impairment and multimorbidity in a representative sample of the Spanish population. BMC Public Health. 2014; 14: 815.
Sri Lanka	Subnational	No	Both	blind, moderate, severe	2006	2007	Edussuriya K, Sennanayake S, Senaratne T, Marshall D, Sullivan T, Selva D, Casson RJ. The prevalence and causes of visual impairment in central Sri Lanka: the Kandy Eye study. Ophthalmology. 2009; 116(1): 52-6.
Sudan	Subnational	Yes	Both	MSVI, blind, moderate, severe	2010	2010	International Centre for Eye Health (ICEH). Sudan - North Kordofan Rapid Assessment of Avoidable Blindness 2010. Grootebroek, Netherlands: RAAB Repository.
Sudan	Subnational	Yes	Both	MSVI, blind, moderate, severe	2010	2010	International Centre for Eye Health (ICEH). Sudan - Northern Rapid Assessment of Avoidable Blindness 2009-2010. Grootebroek, Netherlands: RAAB Repository.
Sudan	Subnational	Yes	Both	MSVI, blind, moderate, severe	2010	2010	International Centre for Eye Health (ICEH). Sudan - Sennar Rapid Assessment of Avoidable Blindness 2010. Grootebroek, Netherlands: RAAB Repository.
Sudan	Subnational	Yes	Both	MSVI, blind,	2010	2010	International Centre for Eye Health (ICEH). Sudan - White Nile Rapid

				moderate, severe			Assessment of Avoidable Blindness Survey 2010. Grootebroek, Netherlands: RAAB Repository, 2010.
Suriname	National	Yes	Presenting	MSVI, blind, moderate, severe	2013	2014	Minderhoud J, Pawiroredjo JC, Themen HCI, Bueno de Mesquita-Voigt A-MT, Siban MR, Forster-Pawiroredjo CM, Limburg H, van Nispen RMA, Mans DRA, Moll AC. Blindness and Visual Impairment in the Republic of Suriname. *Ophthalmology*. 2015; 122(10): 2147–9.
Sweden	Subnational	No	Presenting	blind, moderate, severe	1982	1993	Kvarnström G, Jakobsson P, Lennerstrand G. Visual screening of Swedish children: an ophthalmological evaluation. *Acta Ophthalmol Scand*. 2001; 79(3): 240-4.
Taiwan (Province of China)	Subnational	No	Both	MSVI, blind, moderate, severe	2009	2011	Chen S-J, Cheng C-Y, Li A-F, Peng K-L, Chou P, Chiou S-H, Hsu W-M. Prevalence and associated risk factors of myopic maculopathy in elderly Chinese: the Shihpai eye study. Invest Ophthalmol Vis Sci. 2012; 53(8): 4868-73.
Taiwan (Province of China)	Subnational	No	Both	MSVI, blind, mild	1999	2000	Hsu WM, Cheng CY, Liu JH, Tsai SY, Chou P. Prevalence and causes of visual impairment in an elderly Chinese population in Taiwan: the Shihpai Eye Study. Ophthalmology. 2004; 111(1): 62-9.
Taiwan (Province of China)	Subnational	No	Best-corrected	MSVI, blind, moderate, severe	1993	1995	Liu JH, Cheng CY, Chen SJ, Lee FL. Visual impairment in a Taiwanese population: prevalence, causes, and socioeconomic factors. Ophthalmic Epidemiol. 2001; 8(5): 339-50.
Taiwan (Province of China)	National	No	Best-corrected	blind, moderate, severe	2002	2002	Tsai C-Y, Woung L-C, Chou P, Yang C-S, Sheu M-M, Wu J-R, Chuang T-L, Tung T-H. The current status of visual disability in the elderly population of Taiwan.

							Jpn J Ophthalmol. 2005; 49(2): 166-72.
Thailand	National	Yes	Presenting	MSVI, blind, moderate, severe	2012	2013	Isipradit S, Sirimaharaj M, Charukamnoetkanok P, Thonginnetra O, Wongsawad W, Sathornsumetee B, Somboonthanakij S, Soomsawasdi P, Jitawatanarat U, Taweebanjongsin W, Arayangkoon E, Arame P, Kobkoonthon C, Pangputhipong P. The first rapid assessment of avoidable blindness (RAAB) in Thailand. PLoS One. 2014; 9(12): e114245.
Thailand	Subnational	No	Both	blind, moderate, severe	1997	1998	Singalavanija A, Metheetrairut A, Ruangvaravate N, Tuchinda R, Wanumkarng N. Ocular diseases and blindness in elderly Thais. J Med Assoc Thai. 2001; 84(10): 1383-8.
Thailand	National	No	Presenting	MSVI, blind, moderate, severe	1994	1995	Thailand National Survey of Blindness and Low Vision 1994. [Unpublished].
Timor-Leste	National	Yes	Both	MSVI, blind, moderate, severe	2016	2016	International Centre for Eye Health (ICEH). Timor Leste Rapid Assessment of Avoidable Blindness 2016. Grootebroek, Netherlands: RAAB Repository.
Timor-Leste	National	Yes	Presenting	blind, moderate, severe	2009	2010	Ramke J, Brian G, Naduvilath T, Lee L, Qoqonokana MQ. Prevalence and Causes of Blindness and Low Vision Revisited after 5 years of Eye Care in Timor- Leste. Ophthalmic Epidemiol. 2012; 19(2): 52-7.
Tonga	National	No	Both	MSVI, blind, moderate, severe	1991	1991	Newland HS, Woodward AJ, Taumoepeau LA, Karunaratne NS, Duguid IG. Epidemiology of blindness and visual impairment in the kingdom of Tonga. Br J Ophthalmol. 1994; 78(5): 344-8.

Trinidad and Tobago	National	No	Presenting	blind, mild, moderate, near vision loss, severe	2013	2014	Braithwaite T, Bartholomew D, Deomansingh F, Fraser A, Maharaj V, Bridgemohan P, Sharma SC, Singh DP, Ramsewak SS, Bourne RRA. The prevalence and causes of blindness and vision impairment in Trinidad and Tobago. Invest Ophthalmol Vis Sci. 2015; 56(7).
Tunisia	National	No	Presenting	blind, moderate, severe	1993	1993	Ayed S, Négrel AD, Nabli M, Kamel N, Jebri AM, Siddhom M. [Prevalence and causes of blindness in the Tunisian Republic. Results of a national survey conducted in 1993. Tunisian Team on the Evaluation of Blindness]. *Sante*. 1998; 8(4): 275-82.
Turkey	Subnational	No	Presenting	blind, moderate, severe	1989	1989	Negrel AD, Minassian DC, Sayek F. Blindness and low vision in southeast Turkey. Ophthalmic Epidemiol. 1996; 3(3): 127-34.
Turkmenistan	National	Yes	Presenting	MSVI, blind, moderate, severe	2000	2001	Amansakhatov S, Volokhovskaya ZP, Afanasyeva AN, Limburg H. Cataract blindness in Turkmenistan: results of a national survey. Br J Ophthalmol. 2002; 86(11): 1207-10.
Uganda	Subnational	Yes	Both	MSVI, blind, moderate, severe	2013	2013	International Centre for Eye Health (ICEH). Uganda - Hoima Rapid Assessment of Avoidable Blindness 2013. Grootebroek, Netherlands: RAAB Repository.
Uganda	Subnational	Yes	Both	MSVI, blind, moderate, severe	2014	2015	International Centre for Eye Health (ICEH). Uganda - Karamoja Rapid Assessment of Avoidable Blindness Survey 2015. Grootebroek, Netherlands: RAAB Repository.[Forthcoming].

Uganda	Subnational	Yes	Both	MSVI, blind, moderate, severe	2012	2012	International Centre for Eye Health (ICEH). Uganda - Mubende Rapid Assessment of Avoidable Blindness 2012. Grootebroek, Netherlands: RAAB Repository.
Uganda	Subnational	Yes	Both	MSVI, blind, moderate, severe	2011	2011	International Centre for Eye Health (ICEH). Uganda - Western Ntungamo Rapid Assessment of Avoidable Blindness Survey 2011. Grootebroek, Netherlands: RAAB Repository. [Forthcoming}.
Uganda	Subnational	No	Presenting	MSVI, blind, moderate, severe	1999	2000, 2001	Mbulaiteye SM, Reeves BC, Karabalinde A, Ruberantwari A, Mulwanyi F, Whitworth JAG, Johnson GJ. Evaluation of E-optotypes as a screening test and the prevalence and causes of visual loss in a rural population in SW Uganda. Ophthalmic Epidemiol. 2002; 9(4): 251-62.
United Kingdom	Subnational	No	Best-corrected	blind, moderate, severe	1982	1984	Gibson JM, Lavery JR, Rosenthal AR. Blindness and partial sight in an elderly population. Br J Ophthalmol. 1986; 70(9): 700-5.
United Kingdom	Subnational	No	Presenting	blind, moderate, severe	2004	2011	Khawaja AP, Chan MPY, Hayat S, Broadway DC, Luben R, Garway-Heath DF, Sherwin JC, Yip JLY, Dalzell N, Wareham NJ, Khaw K-T, Foster PJ. The EPIC-Norfolk Eye Study: rationale, methods and a cross-sectional analysis of visual impairment in a population-based cohort. BMJ Open. 2013; 3(3).
United Kingdom	Subnational	No	Both	moderate, severe	1994	1995	Van der Pols JC, Bates CJ, McGraw PV, Thompson JR, Reacher M, Prentice A, Finch S. Visual acuity measurements in a national sample of British elderly people. Br J Ophthalmol. 2000; 84(2): 165-70.

United Kingdom	Subnational	No	Presenting	blind, mild, moderate, severe	1989	1990, 1991	Wormald RP, Wright LA, Courtney P, Beaumont B, Haines AP. Visual problems in the elderly population and implications for services. BMJ. 1992; 304(6836): 1226-9.
United Republic of Tanzania	Subnational	Yes	Both	MSVI, blind, moderate, severe	2007	2007	Habiyakire C, Kabona G, Courtright P, Lewallen S. Rapid assessment of avoidable blindness and cataract surgical services in Kilimanjaro region, Tanzania. Ophthalmic Epidemiol. 2010; 17(2): 90-4.
United Republic of Tanzania	Subnational	Yes	Both	MSVI, blind, moderate, severe	2011	2011	International Centre for Eye Health (ICEH). Tanzania - Kigoma Rapid Assessment of Avoidable Blindness Survey 2011. Grootebroek, Netherlands: RAAB Repository, 2010. [Unpublished].
United Republic of Tanzania	Subnational	Yes	Both	MSVI, blind, moderate, severe	2007	2007	International Centre for Eye Health (ICEH). Tanzania - Kilimanjaro Rapid Assessment of Avoidable Blindness 2007. Grootebroek, Netherlands: RAAB Repository.
United Republic of Tanzania	Subnational	Yes	Both	MSVI, blind, moderate, severe	2017	2017	International Centre for Eye Health (ICEH). Tanzania - Singida Rapid Assessment of Avoidable Blindness Survey 2017. Grootebroek, Netherlands: RAAB Repository.
United Republic of Tanzania	Subnational	Yes	Both	MSVI, blind, moderate, severe	2007	2007	International Centre for Eye Health (ICEH). Tanzania - Zanzibar Rapid Assessment of Avoidable Blindness 2007.
United Republic of Tanzania	Subnational	No	Both	blind, moderate, severe	1986	1986	Rapoza PA, West SK, Katala SJ, Taylor HR. Prevalence and causes of vision loss in central Tanzania. Int J Ophthalmol. 1991; 15(2): 123-9.
United States of America	Subnational	No	Best-corrected	blind, moderate, severe	1991	1991	Boyle CA, Yeargin-Allsopp M, Doernberg NS, Holmgreen P, Murphy CC, Schendel DE, Centers for Disease

							Control and Prevention (CDC). Prevalence of selected developmental disabilities in children 3-10 years of age: the Metropolitan Atlanta Developmental Disabilities Surveillance Program, 1991. MMWR Surveill Summ. 1996; 45(2): 1-14.
United States of America	Subnational	No	Presenting	blind, moderate, severe	1979	1988	Choi TB, Lee DA, Oelrich FO, Amponash D, Bateman JB, Christensen RE. A retrospective study of eye disease among first grade children in Los Angeles. J Am Optom Assoc. 1995; 66(8): 484-8.
Uruguay	National	Yes	Both	MSVI, blind, moderate, severe	2011	2011	International Centre for Eye Health (ICEH). Uruguay Rapid Assessment of Avoidable Blindness 2011.
Vanuatu	National	No	Both	blind, moderate, severe	1989	1989	Newland HS, Harris MF, Walland M, McKnight D, Galbraith JE, Iwasaki W, Momomura K. Epidemiology of blindness and visual impairment in Vanuatu. Bull World Health Organ. 1992; 70(3): 369-72.
Venezuela (Bolivarian Republic of)	National	Yes	Both	MSVI, blind, moderate, severe	2005	2005	International Centre for Eye Health (ICEH). Venezuela Rapid Assessment of Avoidable Blindness Survey 2004. Grootebroek, Netherlands: RAAB Repository.
Viet Nam	Subnational	Yes	Both	MSVI, blind, moderate, severe	2007	2007	International Centre for Eye Health (ICEH). Vietnam - Ba Ria-Vung Tau Rapid Assessment of Avoidable Blindness 2007. Grootebroek, Netherlands: RAAB Repository.
Viet Nam	Subnational	Yes	Both	MSVI, blind, moderate, severe	2007	2007	International Centre for Eye Health (ICEH). Vietnam - Bac Ninh Rapid Assessment of Avoidable Blindness 2007. Grootebroek, Netherlands: RAAB Repository.

Viet Nam	Subnational	Yes	Both	MSVI, blind, mild, moderate, severe	2015	2015	International Centre for Eye Health (ICEH). Vietnam - Bac Ninh Rapid Assessment of Avoidable Blindness Survey 2015. Grootebroek, Netherlands: RAAB Repository.
Viet Nam	Subnational	Yes	Both	MSVI, blind, moderate, severe	2007	2007	International Centre for Eye Health (ICEH). Vietnam - Binh Dinh Rapid Assessment of Avoidable Blindness 2007. Grootebroek, Netherlands: RAAB Repository.
Viet Nam	Subnational	Yes	Both	MSVI, blind, moderate, severe	2001	2001	International Centre for Eye Health (ICEH). Vietnam - Binh Dinh Rapid Assessment of Cataract Surgical Services 2001. Grootebroek, Netherlands: RAAB Repository.
Viet Nam	Subnational	Yes	Both	MSVI, blind, mild, moderate, severe	2015	2015	International Centre for Eye Health (ICEH). Vietnam - Binh Duong Rapid Assessment of Avoidable Blindness Survey 2015. Grootebroek, Netherlands: RAAB Repository.
Viet Nam	Subnational	Yes	Both	MSVI, blind, moderate, severe	2007	2007	International Centre for Eye Health (ICEH). Vietnam - Binh Phuoc Rapid Assessment of Avoidable Blindness 2007. Grootebroek, Netherlands: RAAB Repository.
Viet Nam	Subnational	Yes	Both	MSVI, blind, moderate, severe	2002	2002	International Centre for Eye Health (ICEH). Vietnam - Binh Phuoc Rapid Assessment of Cataract Surgical Services 2002. Grootebroek, Netherlands: RAAB Repository.
Viet Nam	Subnational	Yes	Both	MSVI, blind, mild, moderate, severe	2015	2015	International Centre for Eye Health (ICEH). Vietnam - Ca Mau Rapid Assessment of Avoidable Blindness Survey 2015. Grootebroek, Netherlands: RAAB Repository.

Viet Nam	Subnational	Yes	Both	MSVI, blind, moderate, severe	2007	2007	International Centre for Eye Health (ICEH). Vietnam - Can Tho Rapid Assessment of Avoidable Blindness 2007. Grootebroek, Netherlands: RAAB Repository.
Viet Nam	Subnational	Yes	Both	MSVI, blind, mild, moderate, severe	2015	2015	International Centre for Eye Health (ICEH). Vietnam - Dien Bien Rapid Assessment of Avoidable Blindness Survey 2015. Grootebroek, Netherlands: RAAB Repository.
Viet Nam	Subnational	Yes	Both	MSVI, blind, moderate, severe	2007	2007	International Centre for Eye Health (ICEH). Vietnam - Gia Lai Rapid Assessment of Avoidable Blindness 2007. Grootebroek, Netherlands: RAAB Repository.
Viet Nam	Subnational	Yes	Both	MSVI, blind, mild, moderate, severe	2015	2015	International Centre for Eye Health (ICEH). Vietnam - Gia Lai Rapid Assessment of Avoidable Blindness Survey 2015. Grootebroek, Netherlands: RAAB Repository.
Viet Nam	Subnational	Yes	Both	MSVI, blind, moderate, severe	2002	2002	International Centre for Eye Health (ICEH). Vietnam - Gia Lai Rapid Assessment of Cataract Surgical Services 2002. Grootebroek, Netherlands: RAAB Repository.
Viet Nam	Subnational	Yes	Both	MSVI, blind, moderate, severe	2007	2007	International Centre for Eye Health (ICEH). Vietnam - Ha Tay Rapid Assessment of Avoidable Blindness 2007. Grootebroek, Netherlands: RAAB Repository.
Viet Nam	Subnational	Yes	Both	MSVI, blind, moderate, severe	2000	2000	International Centre for Eye Health (ICEH). Vietnam - Ha Tay Rapid Assessment of Cataract Surgical Services 2000. Grootebroek, Netherlands: RAAB Repository.
Viet Nam	Subnational	Yes	Both	MSVI, blind,	2007	2007	International Centre for Eye Health (ICEH). Vietnam - Hai Phong

				moderate , severe			Rapid Assessment of Avoidable Blindness 2007. Grootebroek, Netherlands: RAAB Repository.
Viet Nam	Subnational	Yes	Both	MSVI, blind, moderate , severe	2000	2000	International Centre for Eye Health (ICEH). Vietnam - Hai Phong Rapid Assessment of Cataract Surgical Services 2000. Grootebroek, Netherlands: RAAB Repository.
Viet Nam	Subnational	Yes	Both	MSVI, blind, moderate , severe	2007	2007	International Centre for Eye Health (ICEH). Vietnam - Ho Chi Minh Rapid Assessment of Avoidable Blindness 2007. Grootebroek, Netherlands: RAAB Repository.
Viet Nam	Subnational	Yes	Presenting	MSVI, blind, moderate , severe	2007	2007	International Centre for Eye Health (ICEH). Vietnam - Hue Rapid Assessment of Avoidable Blindness 2007. Grootebroek, Netherlands: RAAB Repository.
Viet Nam	Subnational	Yes	Both	MSVI, blind, mild, moderate , severe	2015	2015	International Centre for Eye Health (ICEH). Vietnam - Lam Dong Rapid Assessment of Avoidable Blindness Survey 2015. Grootebroek, Netherlands: RAAB Repository.
Viet Nam	Subnational	Yes	Both	MSVI, blind, moderate , severe	2007	2007	International Centre for Eye Health (ICEH). Vietnam - Lao Cai Rapid Assessment of Avoidable Blindness 2007. Grootebroek, Netherlands: RAAB Repository.
Viet Nam	Subnational	Yes	Both	MSVI, blind, mild, moderate , severe	2015	2015	International Centre for Eye Health (ICEH). Vietnam - Nam Dinh Rapid Assessment of Avoidable Blindness Survey 2015. Grootebroek, Netherlands: RAAB Repository.
Viet Nam	Subnational	Yes	Both	MSVI, blind, moderate , severe	2007	2007	International Centre for Eye Health (ICEH). Vietnam - Nghe An Rapid Assessment of

							Avoidable Blindness 2007. Grootebroek, Netherlands: RAAB Repository.
Viet Nam	Subnational	Yes	Both	MSVI, blind, moderate, severe	2012	2012	International Centre for Eye Health (ICEH). Vietnam - Nghe An Rapid Assessment of Avoidable Blindness 2012. Grootebroek, Netherlands: RAAB Repository.
Viet Nam	Subnational	Yes	Both	MSVI, blind, moderate, severe	2000	2000	International Centre for Eye Health (ICEH). Vietnam - Nghe An Rapid Assessment of Cataract Surgical Services 2000. Grootebroek, Netherlands: RAAB Repository.
Viet Nam	Subnational	Yes	Both	MSVI, blind, moderate, severe	2007	2007	International Centre for Eye Health (ICEH). Vietnam - Ninh Thuan Rapid Assessment of Avoidable Blindness 2007. Grootebroek, Netherlands: RAAB Repository.
Viet Nam	Subnational	Yes	Both	MSVI, blind, moderate, severe	2007	2007	International Centre for Eye Health (ICEH). Vietnam - Phu Tho Rapid Assessment of Avoidable Blindness 2007. Grootebroek, Netherlands: RAAB Repository.
Viet Nam	Subnational	Yes	Both	MSVI, blind, mild, moderate, severe	2015	2015	International Centre for Eye Health (ICEH). Vietnam - Phu Tho Rapid Assessment of Avoidable Blindness Survey 2015. Grootebroek, Netherlands: RAAB Repository.
Viet Nam	Subnational	Yes	Both	MSVI, blind, moderate, severe	2000	2000	International Centre for Eye Health (ICEH). Vietnam - Phu Tho Rapid Assessment of Cataract Surgical Services 2000. Grootebroek, Netherlands: RAAB Repository.
Viet Nam	Subnational	Yes	Both	MSVI, blind, moderate, severe	2012	2012	International Centre for Eye Health (ICEH). Vietnam - Quang Nam Rapid Assessment of Avoidable Blindness 2012. Grootebroek,

							Netherlands: RAAB Repository.
Viet Nam	Subnational	Yes	Both	MSVI, blind, mild, moderate, severe	2015	2015	International Centre for Eye Health (ICEH). Vietnam - Quang Ngai Rapid Assessment of Avoidable Blindness Survey 2015. Grootebroek, Netherlands: RAAB Repository.
Viet Nam	Subnational	Yes	Both	MSVI, blind, mild, moderate, severe	2015	2015	International Centre for Eye Health (ICEH). Vietnam - Quang Tri Rapid Assessment of Avoidable Blindness Survey 2015. Grootebroek, Netherlands: RAAB Repository.
Viet Nam	Subnational	Yes	Both	MSVI, blind, moderate, severe	2010	2010	International Centre for Eye Health (ICEH). Vietnam - Son La Rapid Assessment of Avoidable Blindness 2010. Grootebroek, Netherlands: RAAB Repository.
Viet Nam	Subnational	Yes	Both	MSVI, blind, moderate, severe	2007	2007	International Centre for Eye Health (ICEH). Vietnam - Thai Nguyen Rapid Assessment of Avoidable Blindness 2007. Grootebroek, Netherlands: RAAB Repository.
Viet Nam	Subnational	Yes	Both	MSVI, blind, moderate, severe	2011	2011	International Centre for Eye Health (ICEH). Vietnam - Thanh Hoa Rapid Assessment of Avoidable Blindness 2011. Grootebroek, Netherlands: RAAB Repository.
Viet Nam	Subnational	Yes	Both	MSVI, blind, moderate, severe	2007	2007	International Centre for Eye Health (ICEH). Vietnam - Tien Giang Rapid Assessment of Avoidable Blindness 2007. Grootebroek, Netherlands: RAAB Repository.
Viet Nam	Subnational	Yes	Both	MSVI, blind, mild, moderate, severe	2015	2015	International Centre for Eye Health (ICEH). Vietnam - Tien Giang Rapid Assessment of Avoidable Blindness Survey 2015. Grootebroek,

							Netherlands: RAAB Repository.
Viet Nam	Subnational	Yes	Both	MSVI, blind, moderate, severe	2001	2001	International Centre for Eye Health (ICEH). Vietnam - Tien Giang Rapid Assessment of Cataract Surgical Services 2000. Grootebroek, Netherlands: RAAB Repository.
Viet Nam	Subnational	Yes	Both	MSVI, blind, mild, moderate, severe	2015	2015	International Centre for Eye Health (ICEH). Vietnam - Tuyen Quang Rapid Assessment of Avoidable Blindness Survey 2015. Grootebroek, Netherlands: RAAB Repository.
Viet Nam	Subnational	Yes	Both	MSVI, blind, mild, moderate, severe	2015	2015	International Centre for Eye Health (ICEH). Vietnam - Vung Tau Rapid Assessment of Avoidable Blindness Survey 2015. Grootebroek, Netherlands: RAAB Repository.
Viet Nam	Subnational	No	Both	blind, moderate, severe	2007, 2011	2008, 2011	Paudel P, Ramson P, Naduvilath T, Wilson D, Phuong HT, Ho SM, Giap NV. Prevalence of vision impairment and refractive error in school children in Ba Ria - Vung Tau province, Vietnam. Clin Experiment Ophthalmol. 2014; 42(3.0): 217-26.
Yemen	Subnational	Yes	Both	MSVI, blind, moderate, severe	2009	2009	Al-Khatib TK, Ahmed AA, Hameed AS. Rapid assessment of avoidable blindness in amran and lahj governorates of Yemen. *Sudan J Ophthalmol*. 2017; 5(1): 9–16.
Yemen	Subnational	Yes	Presenting	MSVI, blind, moderate, severe	2009	2009	International Centre for Eye Health (ICEH). Yemen - Amran Rapid Assessment of Avoidable Blindness 2009.
Zambia	Subnational	Yes	Presenting	MSVI, blind, moderate, severe	2010	2010	Lindfield R, Griffiths U, Bozzani F, Mumba M, Munsanje J. A Rapid Assessment of Avoidable Blindness in Southern Zambia. PLoS One. 2012; 7(6): e38483.

Zimbabwe	Subnational	Yes	Both	MSVI, blind, moderate, severe	2014	2014	International Centre for Eye Health (ICEH). Zimbabwe - Manicaland Rapid Assessment of Avoidable Blindness Survey 2016. Grootebroek, Netherlands: RAAB Repository.

Table 3. Characteristics of data sources used in the analysis that reported vision impairment from uncorrected presbyopia

Country	Coverage	Rapid	Presenting or best-corrected	Core visual acuity level(s)	Year study started	Year study ended	Reference
Australia	Subnational	No	Presenting	blind, mild, moderate, near vision loss, severe	1992, 1994	1994, 1996	Taylor HR, Livingston PM, Stanislavsky YL, McCarty CA. Visual impairment in Australia: distance visual acuity, near vision, and visual field findings of the Melbourne Visual Impairment Project. Am J Ophthalmol. 1997; 123(3): 328-37.
Brazil	Subnational	No	Presenting	near vision loss	1999	2000	Duarte WR, Barros AJD, Dias-da-Costa JS, Cattan JM. [Prevalence of near vision deficiency and related factors: a population-based study]. Cad Saude Publica. 2003; 19(2): 551-9.
China	Subnational	No	Both	MSVI, blind, moderate, near vision loss, severe	2009	2009	Cheng F, Shan L, Song W, Fan P, Yuan H. Distance- and near-visual impairment in rural Chinese adults in Kailu, Inner Mongolia. Acta Ophthalmol.

							2016; 94(4): 407-13.
China	National	No	Presenting	near vision loss	2007	2010	Ministry of Health (China), National Center for Chronic and Noncommunicable Disease Control and Prevention (China), World Health Organization (WHO). China WHO Study on Global AGEing and Adult Health 2007-2010.
China, India, Nepal, Niger, South Africa, United States of America	Subnational	No	Presenting	near vision loss	2008, 2011	2009, 2011	He M, Abdou A, Naidoo KS, Sapkota YD, Thulasiraj RD, Varma R, Zhao J, Ellwein LB. Prevalence and correction of near vision impairment at seven sites in China, India, Nepal, Niger, South Africa, and the United States. Am J Ophthalmol. 2012; 154(1): 107-116e1.
Eritrea	Subnational	Yes	Presenting	near vision loss	2010	2012	Fai Chan V, Mebrahtu G, Ramson P, Wepo M, Naidoo KS. Prevalence of refractive error and spectacle coverage in Zoba Ma'ekel Eritrea: a rapid assessment of refractive errror. Ophthalmic Epidemiol. 2013; 20(3): 131-7.
Ghana	National	No	Presenting	near vision loss	2007	2008	Ghana Health Service, Ministry of Health (Ghana), University of Ghana, World Health Organization (WHO). Ghana WHO Study on Global AGEing

							and Adult Health 2007-2008.
India	National	No	Presenting	near vision loss	2007	2007	International Institute for Population Sciences (India), World Health Organization (WHO). India WHO Study on Global Ageing and Adult Health 2007. Geneva, Switzerland: World Health Organization (WHO), 2007.
India	Subnational	Yes	Presenting	near vision loss	2011	2013	Marmamula S, Khanna RC, Narsaiah S, Shekhar K, Rao GN. Prevalence of spectacles use in Andhra Pradesh, India: rapid assessment of visual impairment project. Clin Exp Ophthalmol. 2014; 42(3): 227-34.
Kenya	Subnational	No	Presenting	near vision loss	2007	2008	Bastawrous A, Mathenge W, Foster A, Kuper H. Prevalence and predictors of refractive error and spectacle coverage in Nakuru, Kenya: a cross-sectional, population-based study. Int Ophthalmol. 2013; 33(5): 541-8.
Kenya	Subnational	No	Presenting	near vision loss	2011	2011	Kimani K, Lindfield R, Senyonjo L, Mwaniki A, Schmidt E. Prevalence and causes of ocular morbidity in Mbeere District, Kenya. Results of a population-based survey. PLoS One. 2013; 8(8): e70009.

Mexico	National	No	Presenting	near vision loss	2009	2010	National Institute of Public Health (Mexico), World Health Organization (WHO). Mexico WHO Study on Global AGEing and Adult Health 2009-2010. Geneva, Switzerland: World Health Organization (WHO), 2011.
Nigeria	Subnational	Yes	Presenting	near vision loss	2012	2012	Senyonjo L, Lindfield R, Mahmoud A, Kimani K, Sanda S, Schmidt E. Ocular morbidity and health seeking behaviour in Kwara state, Nigeria: implications for delivery of eye care services. PLoS One. 2014; 9(8): e104128.
Nigeria	Subnational	No	Presenting	near vision loss	2010	2010	Uche JN, Ezegwui IR, Uche E, Onwasigwe EN, Umeh RE, Onwasigwe CN. Prevalence of presbyopia in a rural African community. Rural Remote Health. 2014; 14(3.0): 2731.
Pakistan	Subnational	Yes	Presenting	near vision loss	2002	2004	Pakistan Rapid Assessment of Refractive Error Survey 2002-2004.
Russian Federation	National	No	Presenting	near vision loss	2007	2010	Russian Academy of Medical Science, World Health Organization (WHO). Russia WHO Study on Global AGEing and Adult Health 2007-2010.
Russian Federation	Subnational	No	Presenting	blind, moderate,	2015	2017	Ufa Eye Research Institute. Russia -

				near vision loss, severe			Ural Eye and Medical Study 2015-2017.
South Africa	National	No	Presenting	near vision loss	2007	2008	Department of Health (South Africa), Human Sciences Research Council, World Health Organization (WHO). South Africa WHO Study on Global AGEing and Adult Health 2007-2008. Geneva, Switzerland: World Health Organization (WHO).
Trinidad and Tobago	National	No	Presenting	blind, mild, moderate, near vision loss, severe	2013	2014	Braithwaite T, Bartholomew D, Deomansingh F, Fraser A, Maharaj V, Bridgemohan P, Sharma SC, Singh DP, Ramsewak SS, Bourne RRA. The prevalence and causes of blindness and vision impairment in Trinidad and Tobago. Invest Ophthalmol Vis Sci. 2015; 56(7).
United Republic of Tanzania	Subnational	No	Presenting	near vision loss	2004	2004	Burke AG, Patel I, Munoz B, Kayongoya A, McHiwa W, Schwarzwalder AW, West SK. Population-based study of presbyopia in rural Tanzania. Ophthalmology. 2006; 113(5): 723-7.
United States of America	National	No	Presenting	blind, moderate, near vision loss	1999	2000	National Center for Health Statistics (NCHS), Centers for Disease Control and Prevention (CDC). United States National Health and

							Nutrition Examination Survey 1999-2000. Hyattsville, United States: National Center for Health Statistics (NCHS), Centers for Disease Control and Prevention (CDC).
United States of America	National	No	Presenting	blind, moderate, near vision loss	2001	2002	National Center for Health Statistics (NCHS), Centers for Disease Control and Prevention (CDC). United States National Health and Nutrition Examination Survey 2001-2002. Hyattsville, United States: National Center for Health Statistics (NCHS), Centers for Disease Control and Prevention (CDC).
United States of America	National	No	Presenting	blind, moderate, near vision loss	2003	2004	National Center for Health Statistics (NCHS), Centers for Disease Control and Prevention (CDC). United States National Health and Nutrition Examination Survey 2003-2004. Hyattsville, United States: National Center for Health Statistics (NCHS), Centers for Disease Control and Prevention (CDC).
United States of America	National	No	Presenting	blind, moderate, near vision loss	2005	2006	National Center for Health Statistics (NCHS), Centers for Disease Control and Prevention

							(CDC). United States National Health and Nutrition Examination Survey 2005-2006. Hyattsville, United States: National Center for Health Statistics (NCHS), Centers for Disease Control and Prevention (CDC), 2007.
United States of America	National	No	Presenting	blind, moderate, near vision loss	2007	2008	National Center for Health Statistics (NCHS), Centers for Disease Control and Prevention (CDC). United States National Health and Nutrition Examination Survey 2007-2008. Hyattsville, United States: National Center for Health Statistics (NCHS), Centers for Disease Control and Prevention (CDC), 2009.

Table 4. PRISMA-P checklist

**Section/topic # Checklist item**	**Section/topic # Checklist item**	**Section/topic # Checklist item *[and location in original manuscript submission]***	**Reported on page # *[of original manuscript submission]***
TITLE			
Title	1	Identify the report as a systematic review, meta-analysis, or both.<br><br>*The report is identified as a systematic review and meta-analysis in Abstract and Methods section.*	13, 14
ABSTRACT			
Structured summary	2	Provide a structured summary including, as applicable: background; objectives; data sources; study eligibility criteria, participants, and interventions; study appraisal and synthesis methods; results; limitations; conclusions and implications of key findings; systematic review registration number.<br><br>*This summary is given in the abstract.*	13
INTRODUCTION			
Rationale	3	Describe the rationale for the review in the context of what is already known.<br><br>*The rationale is clearly stated in the Introduction section.*	16
Objectives	4	Provide an explicit statement of questions being addressed with reference to participants, interventions, comparisons, outcomes, and study design (PICOS).<br><br>*The statement is clearly stated in the Introduction section.*	16
METHODS			
Protocol and registration	5	Indicate if a review protocol exists, if and where it can be accessed	16 & Appendix Text 2

		(e.g., Web address), and, if available, provide registration information including registration number.<br><br>*The systematic review strategy was published previously and the reference to this is given [reference 5]. The full search strategy is given in Text 2 of the Appendix*	
Eligibility criteria	6	Specify study characteristics (e.g., PICOS, length of follow-up) and report characteristics (e.g., years considered, language, publication status) used as criteria for eligibility, giving rationale.<br><br>*The eligibility criteria are given in Text 2 of the Appendix, section 2.1. 'Developing the Search Strategy'*	Appendix Text 2
Information sources	7	Describe all information sources (e.g., databases with dates of coverage, contact with study authors to identify additional studies) in the search and date last searched.<br><br>*The information sources are given in Text 2 of the Appendix, section 2.1. 'Developing the Search Strategy' and Section 2.2 'Final Search Strategies'.*	Appendix Text 2
Search	8	Present full electronic search strategy for at least one database, including any limits used, such that it could be repeated.<br><br>*This is given in Text 2 of the Appendix Section 2.2 'Final Search Strategies'.*	Appendix Text 2
Study selection	9	State the process for selecting studies (i.e., screening, eligibility,	18

		included in systematic review, and, if applicable, included in the meta-analysis).<br><br>*The study selection for eligibility and screening is given in the main manuscript under Methods.*	
Data collection process	10	Describe method of data extraction from reports (e.g., piloted forms, independently, in duplicate) and any processes for obtaining and confirming data from investigators.<br><br>*This is stated in the Methods section*	18
Data items	11	List and define all variables for which data were sought (e.g., PICOS, funding sources) and any assumptions and simplifications made.<br><br>*This is stated in the Methods section*	18
Risk of bias in individual studies	12	Describe methods used for assessing risk of bias of individual studies (including specification of whether this was done at the study or outcome level), and how this information is to be used in any data synthesis.<br><br>*The principal risk of bias with data sources resided with data sources that were not population-based, the visual acuity measurement methods were unclear or where the visual acuity cut-offs for vision loss were not compatible with those of this study. These were minimised by exclusion of full-text articles as shown in the PRISMA flowchart (Figure 1 Appendix) and the Methods section that*	18 & Appendix Fig 1

		explains the review by the expert panel.	
Summary measures	13	State the principal summary measures (e.g., risk ratio, difference in means).<br><br>*We estimated crude and age-standardised prevalence of blindness and vision impairment for 2020 and trends in age-standardised prevalence of vision impairment, including analysis of uncertainties, by age, sex, and geographical region. This is detailed in the Methods section*	18
Synthesis of results	14	Describe the methods of handling data and combining results of studies, if done, including measures of consistency (e.g., I^2) for each meta-analysis.<br><br>*These are explained in the final sections of the Methods section.*	18
Risk of bias across studies	15	Specify any assessment of risk of bias that may affect the cumulative evidence (e.g., publication bias, selective reporting within studies).<br><br>*These are described under 'limitations of the study' in the Discussion section.*	29
Additional analyses	16	Describe methods of additional analyses (e.g., sensitivity or subgroup analyses, meta-regression), if done, indicating which were pre-specified.<br><br>*These were not performed.*	N/A
RESULTS			
Study selection	17	Give numbers of studies screened, assessed for eligibility, and included in the review, with reasons for exclusions at	Appendix Fig 1

		each stage, ideally with a flow diagram.<br><br>*This is given as Appendix Fig 1 using a PRISMA flowchart*	
Study characteristics	18	For each study, present characteristics for which data were extracted (e.g., study size, PICOS, follow-up period) and provide the citations.<br><br>*The citations are available in Tables 2 and 3 of the Appendix*	Appendix Tables 2 and 3
Risk of bias within studies	19	Present data on risk of bias of each study and, if available, any outcome level assessment (see item 12).<br><br>*We were unable to comment on this beyond the decision to exclude studies that carried a significant risk of bias as detailed in Point 12 of the checklist.*	18
Results of individual studies	20	For all outcomes considered (benefits or harms), present, for each study: (a) simple summary data for each intervention group (b) effect estimates and confidence intervals, ideally with a forest plot.<br><br>*In terms of prevalence of vision impairment and blindness, the tables in both the manuscript and also the Appendix contain uncertainty intervals around all of the estimates*	Main text and appendix
Synthesis of results	21	Present results of each meta-analysis done, including confidence intervals and measures of consistency.<br><br>*These results are reported with 95% Uncertainty Intervals in the results section and Tables.*	21-25

Risk of bias across studies	22	Present results of any assessment of risk of bias across studies (see Item 15).<br><br>*The statistical model investigated the risk of bias across studies and an account of this can be found in the Appendix*	Appendix
Additional analysis	23	Give results of additional analyses, if done (e.g., sensitivity or subgroup analyses, meta-regression [see Item 16]).<br><br>*Not applicable*	N/A
DISCUSSION			
Summary of evidence	24	Summarize the main findings including the strength of evidence for each main outcome; consider their relevance to key groups (e.g., healthcare providers, users, and policy makers).<br><br>*This is summarised in the discussions section.*	26-30
Limitations	25	Discuss limitations at study and outcome level (e.g., risk of bias), and at review-level (e.g., incomplete retrieval of identified research, reporting bias).<br><br>*The limitations are discussed in the discussion section.*	26-30
Conclusions	26	Provide a general interpretation of the results in the context of other evidence, and implications for future research.<br><br>*Please see the discussion and conclusions section and Research in Context panel*	26-30 and 14-15
FUNDING			
Funding	27	Describe sources of funding for the systematic review and other support (e.g., supply of data); role of	13

		funders for the systematic review.<br><br>*These are given in the abstract section of the manuscript*	

Table 5. Sources searched for the 2018 update

Database/information source	Interface/URL
From original review	
MEDLINE, MEDLINE In-Process, MEDLINE Daily and Epub Ahead of Print	Ovid SP
Embase	Ovid SP
WHOLIS	http://dosei.who.int/uhtbin/webcat
Additional sources for the 2018 update	
SciELO	http://www.scielo.org/php/index.php?lang=en
Open Grey	http://www.opengrey.eu/

Table 6: Literature search results

**Resource**	**Number of records identified**
Epub Ahead of Print, In-Process & Other Non-Indexed Citations, Ovid MEDLINE(R) Daily and Ovid MEDLINE(R) 1946 to Present	14219
Embase	23973
WHOLIS	79
OpenGrey	54
SciELO Citation Index (SCIELO)	709
Website searches	256
**Total number of records retrieved**	**39290**
**Total number of records after deduplication and the removal of pre-1980 records**	**10092**

Table 7. Adjustment factors for non-reference definition data.

Data type*	Logit-Transformed Beta Coefficient (95% CI)			
	Mild Vision Loss	Moderate Vision Loss	Severe Vision Loss	Blindness
Best-corrected visual acuity	-0.83 (-1.51 to -0.12)	-1.11 (-2.27 to 0.06)	-0.94 (-2.30 to 0.42)	-0.15 (-0.19 to -0.15)
Uses rapid methodology	0.09 (-0.88 to 1.06)	-0.06 (-1.23 to 1.11)	0.11 (-1.25 to1.48)	0.07 (-0.03 to 0.34)

*Adjusted to reference definition of presenting visual acuity measured in a non-RAAB methodology study.

Table 8. Fixed effects for sex (study-level covariate), healthcare access and quality index (country-level covariate), and/or sociodemographic index that were included in Dismod models.

Model	Covariate	Exponentiated Coefficient (95% UI)
Mild vision impairment	Socio-demographic index	0.37 (0.19 – 0.73)
Mild vision impairment	Sex	0.83 (0.74 – 0.93)
Moderate vision impairment	Socio-demographic index	0.78 (0.67 – 0.92)
Moderate vision impairment	Sex	0.89 (0.86 – 0.92)
Severe vision impairment	Socio-demographic index	0.38 (0.31 – 0.49)
Severe vision impairment	Sex	0.85 (0.82 – 0.88)
Blindness	Socio-demographic index	0.38 (0.23 – 0.61)
Blindness	Healthcare access & quality index	0.98 (0.98 – 0.99)
Blindness	Sex	0.90 (0.87 – 0.94)
Presbyopia	Socio-demographic index	0.56 (0.33 – 0.89)
Presbyopia	Sex	0.91 (0.44 – 0.98)

Table 9. Health state descriptions and accompanying disability weight.

Health state name	Health state description	Disability weight
Distance vision, mild loss	This person has some difficulty with distance vision, for example reading signs, but no other problems with eyesight.	0.003 (0.001-0.007)
Distance vision, moderate loss	This person has vision problems that make it difficult to recognize faces or objects across a room.	0.031 (0.019–0.049)
Distance vision, severe loss	This person has severe vision loss, which causes difficulty in daily activities, some emotional impact (for example, worry), and some difficulty going outside the home without assistance.	0.184 (0.125–0.258)
Distance vision blindness	This person is completely blind, which causes great difficulty in some daily activities, worry and anxiety, and great difficulty going outside the home without assistance.	0.187 (0.124–0.260)
Near vision loss	This person has difficulty seeing things that are nearer than 3 feet, but has no difficulty with seeing things at a distance.	0.011 (0.005–0.02)

Figure 1. PRISMA flowchart

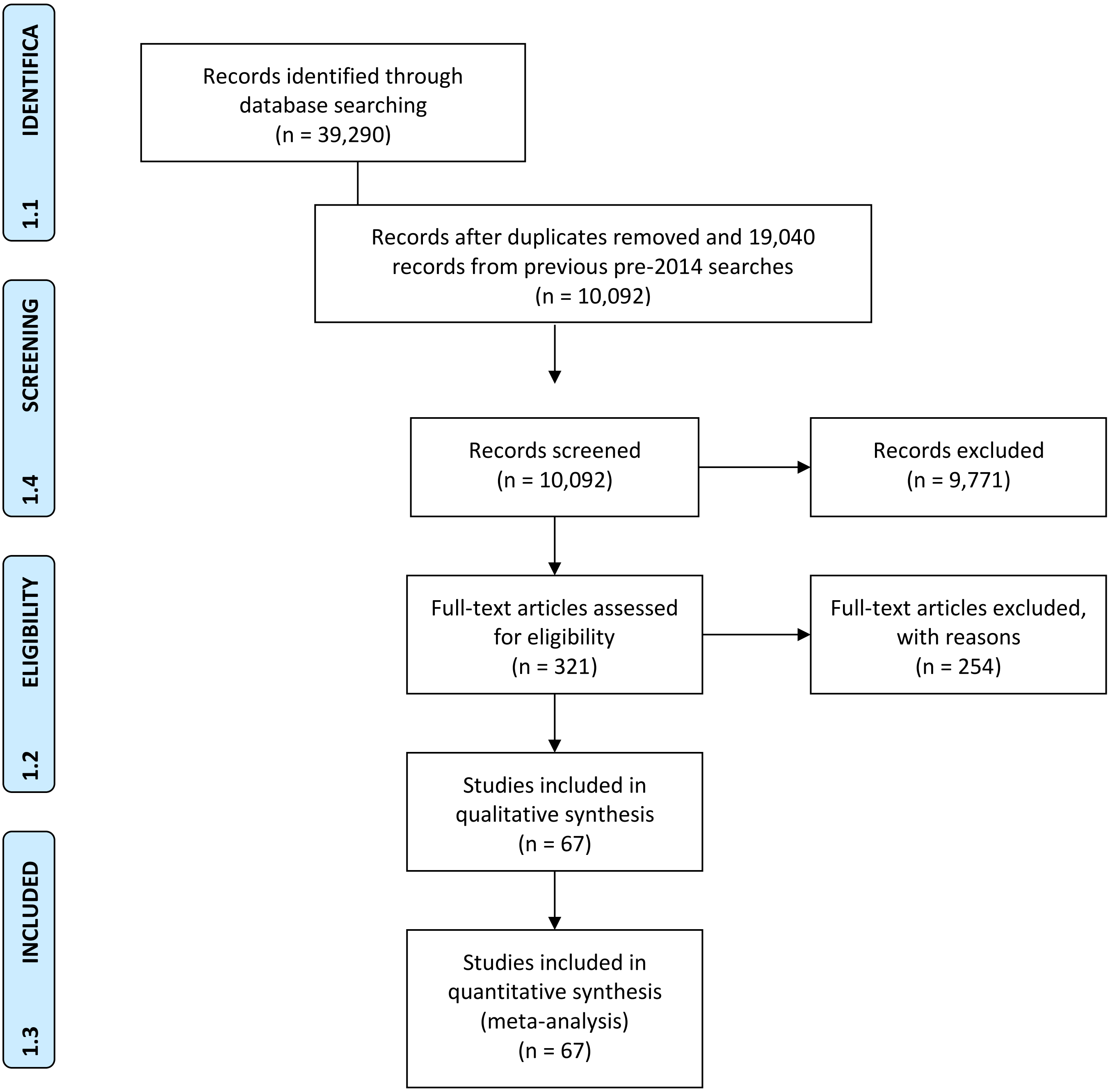

Figure 2. Meta-regression results to split mixed severity data into single severity data points. (Dots display input data that was either included or trimmed in the model; the model fit is displayed as the black line and uncertainty as grey shading).

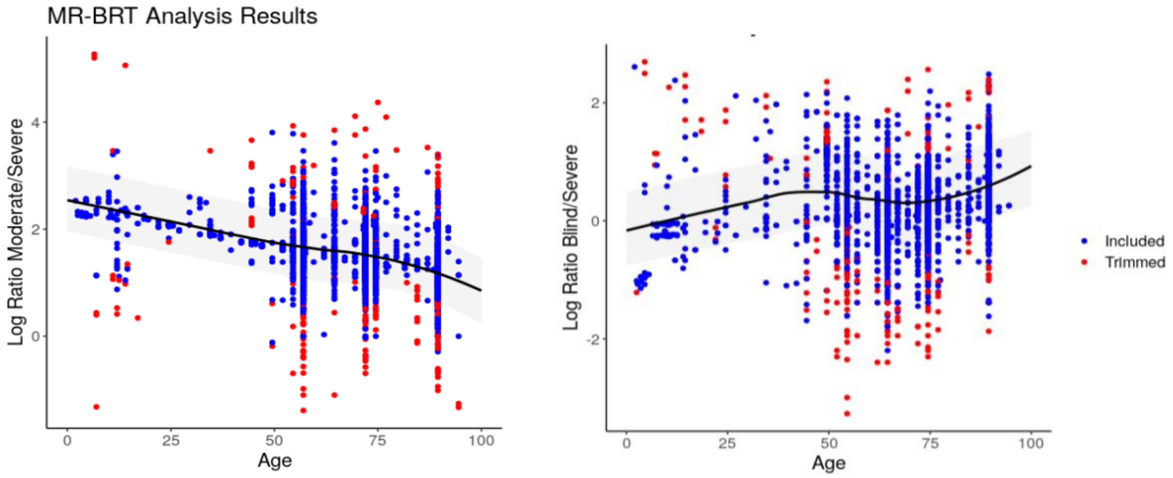

APPENDIX 2: SUPPLEMENTARY RESULTS

Contents

Table 1A. Crude prevalence of moderate and severe vision impairment (MSVI) and blindness in 2020, globally, by region and by sex

Table 1B. Crude prevalence of mild vision impairment and vision impairment from uncorrected presbyopia in 2020, globally, by region and by sex

Table 2. Crude prevalence of blindness, moderate and severe vision impairment, mild vision impairment and vision impairment from uncorrected presbyopia in 2020, globally, by age group

Table 3A. Number of persons affected and age-standardised prevalence of moderate and severe vision impairment (MSVI) and blindness in 2020 and change since 1990 in adults aged 50 years and older.

Table 3B. Number of persons affected and age-standardised prevalence of mild vision impairment and vision impairment from uncorrected presbyopia in 2020 and change since 1990 in adults aged 50 years and older.

Table 4. Number of persons and crude prevalence of persons blind, with MSVI, with mild VI, and vision impairment from uncorrected presbyopia in 1990

Table 5A. Forecasted number of persons and age-standardized prevalence of moderate and severe vision impairment and blindness in 2050 by region

Table 5B. Forecasted number of persons and age-standardized prevalence of mild vision impairment and vision impairment from uncorrected presbyopia in 2050 by region

Figure 1: Data Sources used in the analysis from RAAB (Map A) and non-RAAB (Map B) studies.

Figure 2A-D. Forecast of numbers of people affected by blindness, moderate and severe vision impairment, mild vision impairment and vision impairment from uncorrected presbyopia to 2050- by region.

Table 1A. Crude prevalence of moderate and severe vision impairment (MSVI) and blindness in 2020, globally, by region and by sex

	Moderate and severe vision impairment						Blindness					
Region	Male prevalence per 1,000 (95% UI)	Male cases x100,000 (95% UI)	Female prevalence per 1,000 (95% UI)	Female cases x100,000 (95% UI)	Both sex prevalence per 1,000 (95% UI)	Both sex cases x100,000 (95% UI)	Male prevalence per 1,000 (95% UI)	Male cases x100,000 (95% UI)	Female prevalence per 1,000 (95% UI)	Female cases x100,000 (95% UI)	Both sex prevalence per 1,000 (95% UI)	Both sex cases x100,000 (95% UI)
Andean Latin America	39·8 (35·9–43·6)	12·9 (11·6–14·1)	45·6 (41·5–50·1)	14·7 (13·4–16·2)	42·7 (38·8–46·7)	27·6 (25·1–30·2)	5·20 (4·44–5·94)	1·68 (1·43–1·92)	5·61 (4·82–6·40)	1·81 (1·55–2·06)	5·41 (4·66–6·16)	3·49 (3·01–3·98)
Australasia	24·2 (21·9–26·6)	3·51 (3·18–3·87)	26·7 (24·1–29·2)	3·99 (3·60–4·35)	25·5 (23·0–27·9)	7·50 (6·79–8·21)	2·15 (1·83–2·47)	0·312 (0·266–0·358)	2·52 (2·16–2·90)	0·376 (0·322–0·433)	2·34 (2·00–2·68)	0·689 (0·591–0·788)
Caribbean	29·9 (27·0–32·7)	6·95 (6·28–7·61)	35·9 (32·6–39·4)	8·59 (7·80–9·44)	32·9 (29·8–36·1)	15·5 (14·1–17·0)	5·45 (4·63–6·20)	1·27 (1·08–1·44)	5·54 (4·72–6·38)	1·33 (1·13–1·53)	5·50 (4·68–6·26)	2·60 (2·21–2·96)
Central Asia	26·4 (23·7–29·3)	12·3 (11·1–13·7)	36·0 (32·3–39·9)	17·2 (15·4–19·1)	31·2 (28·1–34·6)	29·5 (26·6–32·7)	2·91 (2·47–3·33)	1·36 (1·16–1·56)	3·45 (2·94–3·95)	1·65 (1·40–1·89)	3·18 (2·71–3·63)	3·01 (2·56–3·44)
Central Europe	27·4 (24·3–30·8)	15·2 (13·5–17·1)	41·6 (36·6–46·6)	24·3 (21·4–27·2)	34·7 (30·6–38·8)	39·5 (34·9–44·2)	2·50 (2·14–2·84)	1·39 (1·19–1·57)	3·23 (2·78–3·70)	1·89 (1·62–2·16)	2·88 (2·46–3·28)	3·27 (2·80–3·73)
Central Latin America	33·6 (30·3–36·7)	43·7 (39·5–47·8)	40·4 (36·5–44·3)	54·7 (49·5–60·0)	37·0 (33·5–40·6)	98·4 (89·1–108)	4·61 (4·00–5·17)	6·00 (5·22–6·74)	4·91 (4·27–5·51)	6·65 (5·78–7·47)	4·76 (4·15–5·34)	12·7 (11·0–14·2)
Central Sub-Saharan Africa	14·6 (13·1–16·3)	9·61 (8·63–10·7)	15·8 (14·2–17·6)	10·5 (9·47–11·7)	15·2 (13·7–16·9)	20·1 (18·1–22·3)	2·00 (1·73–2·29)	1·32 (1·13–1·50)	2·34 (2·01–2·66)	1·56 (1·34–1·77)	2·17 (1·87–2·47)	2·87 (2·47–3·27)
East Asia	30·2 (26·8–33·9)	231 (205–259)	41·9 (37·2–46·9)	308 (274–345)	35·9 (31·9–40·3)	539 (478–604)	5·40 (4·69–6·16)	41·3 (35·9–47·1)	6·74 (5·84–7·66)	49·6 (43·0–56·3)	6·06 (5·26–6·90)	90·9 (78·9–103)
Eastern Europe	39·2 (34·9–44·0)	38·0 (33·9–42·6)	65·0 (57·8–72·3)	72·8 (64·7–81·0)	53·0 (47·2–59·1)	111 (98·6–123)	2·94 (2·55–3·31)	2·85 (2·47–3·21)	4·51 (3·95–5·10)	5·04 (4·42–5·71)	3·78 (3·30–4·26)	7·90 (6·90–8·90)
Eastern Sub-Saharan Africa	15·1 (13·8–16·6)	32·1 (29·3–35·2)	17·6 (16·0–19·3)	37·9 (34·6–41·5)	16·4 (14·9–17·9)	70·1 (63·8–76·7)	4·19 (3·70–4·68)	8·90 (7·87–9·96)	5·01 (4·42–5·61)	10·8 (9·52–12·1)	4·60 (4·06–5·14)	19·7 (17·4–22·0)
High-income Asia Pacific	25·8 (23·3–28·3)	23·7 (21·3–26·0)	31·4 (27·9–34·7)	29·7 (26·4–32·9)	28·6 (25·7–31·4)	53·4 (47·9–58·6)	2·78 (2·43–3·12)	2·55 (2·23–2·86)	2·95 (2·59–3·30)	2·80 (2·46–3·14)	2·87 (2·52–3·20)	5·35 (4·70–5·97)
High-income North America	17·8 (16·2–19·4)	32·2 (29·3–35·2)	22·6 (20·4–24·7)	42·1 (38·1–46·0)	20·2 (18·3–22·0)	74·4 (67·5–81·1)	1·79 (1·57–2·01)	3·24 (2·84–3·65)	2·08 (1·82–2·34)	3·88 (3·40–4·37)	1·94 (1·70–2·18)	7·12 (6·26–8·01)
North Africa and Middle East	32·3 (29·4–35·3)	106 (96·1–116)	37·0 (33·7–40·6)	113 (103–124)	34·6 (31·4–37·8)	218 (199–239)	4·48 (3·86–5·10)	14·6 (12·6–16·7)	5·34 (4·57–6·08)	16·3 (13·9–18·5)	4·90 (4·20–5·57)	30·9 (26·5–35·2)
Oceania	28·0 (25·4–31·0)	1·94 (1·76–2·14)	29·2 (26·4–32·2)	1·91 (1·73–2·11)	28·6 (25·9–31·5)	3·85 (3·49–4·25)	2·65 (2·28–3·03)	0·183 (0·157–0·209)	3·21 (2·75–3·65)	0·210 (0·180–0·239)	2·92 (2·51–3·31)	0·393 (0·338–0·446)
South Asia	47·7 (42·8–53·0)	449 (403–498)	57·0 (51·2–63·3)	514 (461–570)	52·3 (46·9–58·0)	962 (864–1070)	5·90 (5·13–6·62)	55·5 (48·3–62·3)	7·09 (6·15–8·00)	63·9 (55·4–72·0)	6·48 (5·63–7·29)	119 (104–134)
Southeast Asia	38·2 (35·1–41·5)	130 (119–141)	46·4 (42·9–50·0)	158 (146–170)	42·3 (39·0–45·7)	288 (265–311)	6·91 (6·02–7·77)	23·5 (20·4–26·4)	10·6 (9·19–11·9)	36·1 (31·3–40·6)	8·75 (7·58–9·82)	59·5 (51·6–66·8)
Southern Latin America	28·7 (26·0–31·6)	9·45 (8·56–10·4)	34·0 (30·8–37·0)	11·7 (10·6–12·8)	31·4 (28·5–34·3)	21·2 (19·2–23·1)	2·23 (1·91–2·53)	0·734 (0·629–0·833)	2·46 (2·13–2·82)	0·849 (0·735–0·971)	2·35 (2·02–2·67)	1·58 (1·36–1·80)
Southern Sub-Saharan Africa	17·7 (16·1–19·6)	6·99 (6·33–7·71)	20·7 (18·8–22·7)	8·60 (7·81–9·42)	19·3 (17·5–21·1)	15·6 (14·1–17·1)	5·33 (4·64–5·94)	2·10 (1·83–2·34)	6·43 (5·60–7·19)	2·67 (2·32–2·98)	5·90 (5·13–6·58)	4·77 (4·15–5·32)
Tropical Latin America	41·9 (37·9–45·8)	45·9 (41·5–50·2)	50·1 (45·4–54·9)	57·4 (52·0–62·8)	46·1 (41·7–50·4)	103 (93·5–113)	7·40 (6·45–8·26)	8·11 (7·06–9·05)	8·50 (7·45–9·49)	9·73 (8·53–10·9)	7·96 (6·98–8·89)	17·8 (15·6–19·9)
Western Europe	31·4 (28·4–34·4)	67·4 (61·0–73·8)	39·1 (35·0–42·9)	86·8 (77·8–95·3)	35·3 (31·7–38·6)	154 (139–169)	2·77 (2·39–3·16)	5·95 (5·13–6·79)	4·23 (3·63–4·86)	9·39 (8·06–10·8)	3·51 (3·03–4·03)	15·3 (13·2–17·6)
Western Sub-Saharan Africa	19·2 (17·4–21·1)	44·7 (40·5–49·1)	22·4 (20·3–24·6)	54·0 (48·8–59·4)	20·8 (18·9–22·9)	98·6 (89·4–108)	4·79 (4·21–5·38)	11·1 (9·78–12·5)	5·13 (4·50–5·75)	12·4 (10·9–13·9)	4·96 (4·37–5·57)	23·5 (20·7–26·4)
Global	33·4 (30·3–36·8)	1320 (1200–1460)	41·5 (37·5–45·6)	1630 (1470–1790)	37·4 (33·9–41·2)	2950 (2670–3250)	4·90 (4·28–5·48)	194 (169–217)	6·08 (5·30–6·83)	239 (208–268)	5·49 (4·76–6·13)	433 (376–484)

Table 1B. Crude prevalence of mild vision impairment and vision impairment from uncorrected presbyopia in 2020, globally, by region and by sex

	Mild vision impairment						Vision impairment due to uncorrected presbyopia					
Region	Male prevalence per 1,000 (95% UI)	Male cases x100,000 (95% UI)	Female prevalence per 1,000 (95% UI)	Female cases x100,000 (95% UI)	Both sex prevalence per 1,000 (95% UI)	Both sex cases x100,000 (95% UI)	Male prevalence per 1,000 (95% UI)	Male cases x100,000 (95% UI)	Female prevalence per 1,000 (95% UI)	Female cases x100,000 (95% UI)	Both sex prevalence per 1,000 (95% UI)	Both sex cases x100,000 (95% UI)
Andean Latin America	30·9 (27·8–34·4)	10·0 (8·99–11·1)	35·4 (31·9–39·3)	11·4 (10·3–12·7)	33·2 (29·9–36·7)	21·4 (19·3–23·7)	40·8 (29·3–54·5)	13·2 (9·48–17·6)	44·0 (31·6–59·1)	14·2 (10·2–19·1)	42·4 (30·7–56·9)	27·4 (19·8–36·8)
Australasia	12·8 (11·5–14·3)	1·86 (1·67–2·08)	16·2 (14·4–18·0)	2·41 (2·14–2·68)	14·5 (13·0–16·1)	4·27 (3·82–4·75)	9·44 (6·38–13·4)	1·37 (0·928–1·95)	12·5 (8·32–17·7)	1·87 (1·24–2·64)	11·0 (7·42–15·4)	3·24 (2·19–4·54)
Caribbean	33·7 (30·3–37·4)	7·84 (7·05–8·71)	39·3 (35·3–43·7)	9·42 (8·46–10·5)	36·6 (32·9–40·6)	17·3 (15·5–19·2)	60·4 (43·8–81·0)	14·1 (10·2–18·8)	53·9 (38·8–71·8)	12·9 (9·29–17·2)	57·1 (41·4–76·9)	26·9 (19·5–36·3)
Central Asia	20·8 (18·8–23·2)	9·73 (8·79–10·9)	25·7 (23·1–28·6)	12·3 (11·1–13·7)	23·3 (21·0–25·9)	22·0 (19·9–24·4)	43·5 (31·0–58·6)	20·4 (14·5–27·4)	62·8 (44·6–83·5)	30·0 (21·3–39·9)	53·3 (38·0–71·0)	50·4 (35·9–67·1)
Central Europe	15·2 (13·5–16·9)	8·40 (7·48–9·38)	19·6 (17·4–21·9)	11·4 (10·1–12·8)	17·4 (15·5–19·5)	19·8 (17·7–22·2)	87·6 (63·1–118)	48·6 (35·0–65·6)	125 (89·6–169)	73·3 (52·4–98·5)	107 (76·8–145)	122 (87·4–165)
Central Latin America	31·6 (28·4–35·0)	41·2 (37·0–45·6)	36·9 (33·1–40·9)	49·9 (44·8–55·4)	34·3 (30·9–38·1)	91·1 (82·0–101)	45·5 (32·6–60·9)	59·2 (42·5–79·4)	57·2 (41·3–75·7)	77·4 (56·0–103)	51·4 (37·2–68·5)	137 (98·8–182)
Central Sub-Saharan Africa	26·4 (23·5–29·9)	17·3 (15·4–19·6)	31·7 (28·4–35·8)	21·1 (18·9–23·8)	29·1 (25·9–32·8)	38·4 (34·3–43·3)	31·7 (22·2–42·7)	20·8 (14·6–28·0)	39·6 (29·0–53·0)	26·3 (19·3–35·3)	35·6 (25·7–47·7)	47·1 (34·0–63·1)
East Asia	34·1 (30·2–38·2)	260 (231–292)	46·4 (41·0–51·9)	341 (301–382)	40·1 (35·5–44·9)	601 (533–672)	96·9 (69·4–128)	740 (530–979)	122 (86·8–161)	896 (638–1180)	109 (78·1–144)	1640 (1170–2160)
Eastern Europe	21·0 (18·9–23·5)	20·4 (18·3–22·8)	29·5 (26·2–32·9)	33·0 (29·3–36·9)	25·6 (22·9–28·5)	53·4 (47·7–59·6)	94·5 (67·6–125)	91·6 (65·5–122)	151 (109–199)	169 (123–222)	125 (89·8–165)	260 (188–344)
Eastern Sub-Saharan Africa	24·4 (21·8–27·5)	51·8 (46·3–58·5)	28·5 (25·6–32·1)	61·6 (55·1–69·2)	26·5 (23·7–29·9)	113 (101–128)	37·4 (27·8–47·5)	79·4 (59·2–101)	43·2 (32·3–54·9)	93·1 (69·7–118)	40·3 (30·2–51·0)	173 (129–218)
High-income Asia Pacific	43·6 (38·4–48·8)	40·0 (35·3–44·8)	61·5 (54·1–68·7)	58·3 (51·3–65·2)	52·7 (46·4–58·8)	98·4 (86·7–110)	8·98 (5·92–12·8)	8·24 (5·43–11·8)	12·2 (8·37–17·4)	11·6 (7·94–16·5)	10·6 (7·14–15·1)	19·8 (13·3–28·1)
High-income North America	12·9 (11·5–14·3)	23·3 (20·8–25·9)	15·1 (13·4–16·7)	28·1 (25·0–31·2)	14·0 (12·4–15·5)	51·4 (45·8–57·0)	13·2 (9·22–17·8)	23·8 (16·7–32·2)	13·6 (9·72–18·7)	25·5 (18·1–34·9)	13·4 (9·46–18·1)	49·3 (34·8–66·8)
North Africa and Middle East	21·6 (19·6–24·1)	70·6 (64·0–78·7)	24·2 (21·9–26·8)	73·6 (66·7–81·7)	22·8 (20·7–25·4)	144 (131–160)	21·2 (14·9–28·7)	69·3 (48·6–94·0)	24·7 (17·6–33·1)	75·3 (53·7–101)	22·9 (16·3–30·8)	145 (103–195)
Oceania	26·0 (23·3–29·1)	1·80 (1·61–2·01)	30·0 (27–33·6)	1·97 (1·77–2·20)	28·0 (25·1–31·3)	3·76 (3·38–4·21)	27·9 (19·6–37·9)	1·93 (1·36–2·62)	32·0 (22·5–43·1)	2·10 (1·47–2·82)	29·9 (21·1–40·4)	4·03 (2·84–5·44)
South Asia	29·9 (26·9–33·3)	281 (253–313)	35·5 (32·0–39·6)	320 (288–356)	32·6 (29·4–36·3)	601 (541–669)	83·3 (60·9–109)	783 (573–1030)	98·3 (71·7–128)	885 (646–1150)	90·6 (66·3–118)	1670 (1220–2180)
Southeast Asia	41·1 (37·2–45·7)	140 (126–155)	48·6 (44·0–53·6)	166 (150–183)	44·9 (40·6–49·7)	305 (276–338)	36·7 (26·0–50·0)	124 (88·1–170)	50·1 (36·0–67·3)	171 (122–229)	43·4 (31·0–58·5)	295 (211–398)
Southern Latin America	16·8 (15·1–18·6)	5·52 (4·96–6·13)	21·4 (19·1–23·9)	7·36 (6·58–8·22)	19·1 (17·2–21·3)	12·9 (11·6–14·4)	6·31 (4·22–9·05)	2·08 (1·39–2·98)	8·17 (5·52–11·8)	2·82 (1·90–4·07)	7·26 (4·94–10·3)	4·89 (3·32–6·96)
Southern Sub-Saharan Africa	27·3 (24·7–30·4)	10·8 (9·73–12·0)	34·9 (31·6–38·8)	14·5 (13·1–16·1)	31·2 (28·2–34·7)	25·3 (22·9–28·1)	102 (75·9–130)	40·0 (29·9–51·2)	134 (101–169)	55·6 (41·7–70·2)	118 (88·1–150)	95·7 (71·3–121)
Tropical Latin America	34·2 (30·8–38·1)	37·5 (33·7–41·7)	41·4 (37·1–46·1)	47·4 (42·5–52·8)	37·9 (34·0–42·2)	84·9 (76·2–94·6)	36·3 (25·6–48·9)	39·8 (28·0–53·5)	42·5 (30·2–57·9)	48·7 (34·6–66·3)	39·5 (27·9–53·5)	88·4 (62·5–120)
Western Europe	21·4 (19·0–23·8)	45·9 (40·7–51·0)	28·5 (25·1–31·8)	63·2 (55·8–70·7)	25·0 (22·1–27·9)	109 (96·4–122)	8·85 (5·82–12·4)	19·0 (12·5–26·5)	11·7 (7·89–16·6)	25·9 (17·5–36·8)	10·3 (6·90–14·5)	44·9 (30·1–63·4)
Western Sub-Saharan Africa	30·4 (27·2–34·1)	70·6 (63·2–79·3)	37·1 (33·3–41·4)	89·3 (80·3–99·8)	33·8 (30·3–37·8)	160 (144–179)	40·5 (29·6–52·4)	94·0 (68·8–122)	43·9 (32·2–57·2)	106 (77·6–138)	42·2 (30·9–54·8)	200 (146–260)
Global	29·2 (26·4–32·3)	1160 (1040–1280)	36·2 (32·7–40·0)	1420 (1280–1570)	32·7 (29·5–36·2)	2580 (2330–2850)	58·0 (42·1–76·4)	2290 (1670–3020)	71·3 (52·1–92·9)	2800 (2050–3650)	64·6 (47·1–84·5)	5100 (3710–6670)

Table 2. Crude prevalence of blindness, moderate and severe vision impairment, mild vision impairment and vision impairment from uncorrected presbyopia in 2020, globally, by age group

Age group	Mild vision impairment: prevalence per 1,000 (95% UI)	Moderate and severe vision impairment: prevalence per 1,000 (95% UI)	Blindness: prevalence per 1,000 (95% UI)	Vision impairment due to uncorrected presbyopia: prevalence per 1,000 (95% UI)
Early Neonatal	13·8 (8·55–20·6)	3·86 (2·44–5·99)	0·350 (0·194–0·501)	
Late Neonatal	14.0 (8·78–20·8)	3·95 (2·54–6·05)	0·352 (0·197–0·503)	
Post Neonatal	16·3 (11·8–22·4)	5·11 (3·75–7·08)	0·384 (0·231–0·534)	
1 to 4	23·3 (18·5–29·1)	9·17 (7·18–11·5)	0·540 (0·387–0·712)	
5 to 9	25·7 (19·8–33·0)	12·2 (9·31–15·6)	0·745 (0·547–0·974)	
10 to 14	22·4 (17·2–28·5)	12·8 (9·79–16·5)	0·910 (0·703–1·15)	
15 to 19	18·5 (14·3–23·6)	12·9 (9·96–16·3)	1·06 (0·836–1·32)	
20 to 24	15·3 (11·9–19·6)	12·9 (9·78–16·5)	1·19 (0·957–1·49)	
25 to 29	13·9 (10·9–17·5)	12·7 (9·91–15·7)	1·37 (1·11–1·68)	
30 to 34	13·8 (10·3–18·2)	13·1 (10·1–16·2)	1·67 (1·36–2·01)	11·4 (6·86–18·0)
35 to 39	15·9 (12·1–19·9)	16·3 (12·7–20·3)	2·23 (1·79–2·70)	24·0 (14·2–39·0)
40 to 44	19·5 (14·9–25·5)	21·8 (17·0–27·9)	3·19 (2·52–3·99)	49·1 (31·8–72·1)
45 to 49	24·8 (19·2–31·3)	30·9 (24.3–38·7)	4·46 (3·58–5·82)	86·5 (54·0–131)
50 to 54	32·4 (24·2–42·9)	44·3 (34·6–56·2)	6·24 (4·78–7·90)	130 (84·1–186)
55 to 59	45·7 (34·9–58·9)	67·1 (54·4–83·5)	8·38 (6·72–10·4)	179 (111–260)
60 to 64	63·4 (48·1–81·7)	95·7 (76·8–122)	12·2 (9·75–15.1)	224 (146–323)
65 to 69	90·2 (70·2–112)	130 (106–157)	19·0 (14·9–24·2)	269 (172–385)
70 to 74	117 (88·7–147)	165 (133–201)	27·8 (22.2–34·7)	295 (193–419)
75 to 79	141 (113–169)	198 (165–234)	37·6 (29·6–46·8)	317 (209–476)
80 to 84	158 (122–204)	226 (183–279)	46·5 (37·0–56·9)	329 (216–492)
85 to 89	167 (134–203)	252 (213–297)	56·6 (45·3–69.5)	319 (207–467)
90 to 94	174 (133–217)	275 (229–329)	68·6 (55·5–85·9)	296 (182–437)
95 plus	190 (145–244)	299 (244–368)	91·5 (73·3–115)	269 (146–411)
All-age	32·7 (29·5–36·2)	37·4 (33·9–41·2)	5·49 (4·76–6·13)	64·6 (47·1–84·5)
Age 50+	75·3 (64·3–85·9)	109 (96·1–123)	17·7 (15·1–20·3)	221 (155–296)

Table 3A. Number of persons affected and age-standardised prevalence of moderate and severe vision impairment (MSVI) and blindness in 2020 and change since 1990 in adults aged 50 years and older.

Region	Moderate and severe vision impairment				Blindness			
	Cases in 1000s (95% UI)	% change 1990 to 2020 (95% UI)	Age-standardized rate per 1,000 (95% UI)	% change 1990 to 2020 (95% UI)	Cases in 1000s (95% UI)	% change 1990 to 2020 (95% UI)	Age-standardized rate per 1,000 (95% UI)	% change 1990 to 2020 (95% UI)
Andean Latin America	1620 (1440–1830)	189·1 (181·3–197·4)	130 (115–146)	-4·1 (-6·5–-1·7)	275 (229–323)	79·7 (70·9–87·1)	22.0 (18·3–25·9)	-43·3 (-46·0–-41·3)
Australasia	472 (413–532)	137·0 (125·6–149·0)	40·7 (35·5–46·0)	1·6 (-3·3–6·3)	57·5 (48·4–67·3)	94.0 (83·8–105.0)	4·87 (4·10–5·68)	-19·7 (-23·2–-16·1)
Caribbean	967 (847–1090)	106·6 (102·5–110·7)	82·2 (72·0–92·9)	-3·7 (-5·3–-1·9)	205 (169–241)	53·9 (50·3–57·6)	17·4 (14·4–20·5)	-30.0 (-31·3–-28·8)
Central Asia	1930 (1680–2220)	57·0 (53·8–60·4)	120 (105–135)	-4·2 (-5·9–-2·7)	222 (183–261)	17·2 (13·7–20·6)	14·9 (12·5–17·5)	-26·6 (-28·0–-25·5)
Central Europe	3270 (2830–3740)	48·1 (44·1–52·3)	67·8 (58·8–77·1)	-2·8 (-3·8–-1·8)	279 (236–323)	23·3 (19·5–27·4)	5·79 (4·91–6·68)	-22·8 (-24·0–-21·5)
Central Latin America	5760 (5050–6510)	197·3 (192·8–202·2)	107 (94·1–121)	-7·5 (-8·5–-6·6)	970 (826–1110)	97·6 (93·7–102.0)	18·3 (15·6–21·1)	-41·0 (-41·8–-40·1)
Central Sub-Saharan Africa	1030 (885–1200)	131·2 (124·1–138·9)	99·4 (86·8–113)	-3·2 (-5·7–0·2)	167 (137–195)	80·4 (74·6–87·6)	17·8 (14·8–20·8)	-26·6 (-28·8–-23·8)
East Asia	44900 (39100–51200)	206·7 (200·1–212·8)	94·9 (83·0–107)	8·5 (7·1–9·9)	7260 (6130–8400)	105.0 (97·4–114.0)	15·6 (13·2–18·0)	-29·0 (-32·0–-25·7)
Eastern Europe	8940 (7810–10200)	25·0 (22·7–27·3)	115 (100–130)	-2·3 (-3·3–-1·3)	682 (587–778)	-0·7 (-3·4–2·3)	8·79 (7·59–10·0)	-27·4 (-28·5–-26.4)
Eastern Sub-Saharan Africa	3600 (3190–4080)	105·7 (102·2–109·0)	112 (100–125)	-7·2 (-8·5–-6·1)	1200 (1020–1390)	62·4 (59·7–65·1)	40·0 (33·8–46·1)	-27·0 (-28·1–-25·8)
High-income Asia Pacific	4080 (3580–4570)	154·7 (143·5–166·7)	38.6 (33·9–43·4)	1·6 (0·7–2·5)	450 (392–512)	84·7 (73·9–96·2)	4·24 (3·68–4·78)	-26·8 (-28·2–-25·5)
High-income North America	4750 (4180–5340)	83·8 (81·6–86·0)	32·8 (28·8–37·0)	-1·4 (-2·1–-0·6)	591 (512–675)	88·5 (85·8–91·7)	3·98 (3·45–4·52)	-0·5 (-1·3–-0·3)
North Africa and Middle East	12000 (10500–13600)	153·0 (147·8–158·6)	130 (115–147)	-7·1 (-8·9–-5·0)	2350 (1940–2740)	58·2 (54.0–62·6)	27·0 (22·2–31·7)	-43.0 (-44·1–-41·9)
Oceania	222 (193–253)	126·8 (119·4–134·0)	173 (154–193)	-1·8 (-4·4–0·9)	23·1 (19.0–27·4)	74·2 (68·7–80.0)	19·8 (16·4–23·3)	-24·9 (-26·8–-22·8)
South Asia	68800 (60100–79000)	127·6 (122·6–133·4)	229 (203–260)	-12·6 (-13·8–-11·6)	9580 (8170–11000)	44·7 (40·1–49.0)	35·3 (30·2–40·7)	-47·6 (-48·4–-46.9)
Southeast Asia	19800 (18100–21800)	140·7 (134·1–148·2)	154 (141–168)	-7·7 (-10·0–-5·2)	4550 (3860–5240)	60·6 (58.0–63·1)	37·2 (31·8–42·6)	-40·1 (-41·1–-39·2)
Southern Latin America	1240 (1090–1400)	91·2 (84·9–98·1)	65·9 (58·0–74·1)	-2·1 (-5·2–1·0)	125 (105–147)	44·9 (39·5–50·3)	6·58 (5·57–7·69)	-28·7 (-30·8–-26·7)
Southern Sub-Saharan Africa	870 (763–992)	110·8 (108·0–113·4)	72·7 (63·9–81·9)	-3·0 (-4·3–-1·9)	353 (302–407)	52·9 (49.0–56·9)	30·9 (26·3–35·5)	-29·0 (-30·8–-27·3)
Tropical Latin America	5710 (5000–6460)	179·6 (174·8–184·5)	106 (93·0–119)	-5·5 (-6·5–-4·5)	1430 (1230–1640)	124.0 (119.0–129.0)	27.1 (23·2–31·0)	-28·3 (-29·1–-27·5)
Western Europe	11100 (9690–12400)	62·8 (59·2–66·3)	51·5 (45·2–57·9)	-2·8 (-3·8–-1·7)	1350 (1150–1580)	34.0 (28·9–40·1)	6·01 (5·13–6·95)	-23·9 (-25.1–-22·9)
Western Sub-Saharan Africa	5530 (4830–6360)	113·2 (110·6–115·9)	144 (127–163)	-2·9 (-3·6–-2·1)	1490 (1240–1740)	56·9 (54·7–59·1)	42·2 (35.0–49·2)	-27·8 (-28·7–-26·8)
Global	206000 (182000–233000)	128·0 (125·1–131·0)	112 (99·0–126)	2·5 (1·9–3·2)	33600 (28600–38500)	62·4 (59·6–65·0)	18·5 (15·7–21·1)	-28·5 (-29·4–-27·7)

Table 3B. Number of persons affected and age-standardised prevalence of mild vision impairment and vision impairment from uncorrected presbyopia in 2020 and change since 1990 in adults aged 50 years and older.

Region	Mild vision impairment				Vision impairment due to uncorrected presbyopia			
	Cases in 1000s (95% UI)	% change 1990 to 2020 (95% UI)	Age-standardized rate per 1,000 (95% UI)	% change 1990 to 2020 (95% UI)	Cases in 1000s (95% UI)	% change 1990 to 2020 (95% UI)	Age-standardized rate per 1,000 (95% UI)	% change 1990 to 2020 (95% UI)
Andean Latin America	1180 (1010–1350)	168·0 (161·0–177·0)	94·4 (81·1–108)	-10·1 (-12·2–-7·5)	2410 (1690–3290)	186·0 (169·0–204·0)	192 (135–262)	-4·4 (-9·5–1·8)
Australasia	272 (231–314)	129·0 (119·0–141·0)	23·1 (19·6–26·5)	-1·8 (-5·7–3·3)	298 (196–422)	68·1 (29·8–104·0)	26·4 (17·1–37·2)	-24.1 (-42·4–-7·7)
Caribbean	1060 (908–1210)	87·1 (83·1–91·1)	90·0 (77·2–103)	-12·2 (-14–-10·6)	2340 (1640–3180)	97·6 (87·7–107·0)	199 (139.2–270)	-7·1 (-11·6–-2·7)
Central Asia	941 (797–1080)	51·1 (47·1–54·7)	58·2 (49·8–66·3)	-7·8 (-9·4–-6·1)	4360 (3030–5950)	58·6 (51·3–66·7)	268 (189–357)	-3·8 (-7·6–0·1)
Central Europe	1310 (1110–1500)	32·4 (27·5–36·5)	27·5 (23·5–31·6)	-12·2 (-13·3–-11·0)	11400 (8060–15500)	41·5 (34·1–48·6)	235 (168–316)	-6·1 (-8·5–-3·5)
Central Latin America	5190 (4450–5930)	181·0 (177·0–185·0)	96·0 (82·3–109)	-11·5 (-12·5–-10·5)	12200 (8670–16400)	213·0 (204·0–227·0)	226 (161–304)	-1·8 (-4·4–1·3)
Central Sub-Saharan Africa	1010 (844–1180)	111·0 (103·0–118·0)	91·7 (78·8–105)	-11·1 (-13·8–-8·4)	3610 (2490–4950)	124·0 (109·0–139·0)	327 (232–442)	-5·7 (-11·3–0·6)
East Asia	41700 (35300–48000)	145·0 (140·0–150·0)	85·9 (73·1–98·5)	-9·3 (-10·5–-8·2)	142000 (99400–191000)	155·0 (147·0–164·0)	287 (204–385)	-4·6 (-7·2–-1·8)
Eastern Europe	3550 (3010–4070)	19·0 (16·3–21·6)	45·6 (38·8–52·4)	-6·8 (-7·9–-5·7)	24000 (17200–32200)	37·6 (33·4–41·9)	306 (220–409)	8·0 (5·1–11·2)
Eastern Sub-Saharan Africa	2750 (2320–3180)	89·0 (85·8–92·1)	81·6 (70·3–93·1)	-15·2 (-16·4–-14·1)	12700 (9280–16500)	105·0 (98·3–112·0)	368 (271–474)	-8·1 (-11.0–-4·8)
High-income Asia Pacific	7620 (6530–8720)	136·0 (123·0–147·0)	71·2 (61·1–80·7)	-6·3 (-7·2–-5·3)	1860 (1240–2660)	138·0 (112·0–162·0)	18·2 (11·9–25·7)	0·3 (-3·7–4·1)
High-income North America	3290 (2800–3750)	81·5 (78·3–84·6)	22·4 (19·1–25·6)	-1·4 (-2·4–-0·25)	4570 (3190–6190)	84·1 (78·9–89·2)	31·9 (22·1–43·5)	-0·3 (-2·9–2·1)
North Africa and Middle East	5980 (5100–6860)	147·0 (143·0–151·0)	65·2 (55·6–74·6)	-10·3 (-11·4–-9·1)	12200 (8410–16800)	136·0 (127·0–143·0)	132 (92·3–179)	-14·1 (-16·8–-11·3)
Oceania	119 (100–138)	111·0 (103·0–119·0)	90·4 (77·3–103)	-8·7 (-11·4–-5·7)	321 (219–439)	122·0 (106·0–142·0)	240 (167–323)	-4·5 (-10·9–3·2)
South Asia	32100 (27300–36800)	140·0 (134·0–146·0)	108 (92·6–123)	-10·1 (-11.0–-9·3)	124000 (86600–166000)	141·0 (133·0–151·0)	399 (283–532)	-6·0 (-8·4–-3·3)
Southeast Asia	15600 (13400–17800)	124·0 (120·0–129·0)	117 (101–132)	-13·7 (-15·3–-12·2)	25800 (18000–35100)	136·0 (127·0–144·0)	195 (137–264)	-9·7 (-12·6–-6·6)
Southern Latin America	719 (611–823)	83·1 (76·7–89·5)	37·7 (32·1–43·2)	-7·3 (-9·8–-4·5)	438 (291–631)	79·8 (64·6–97·9)	23·2 (15·3–33·5)	-6·0 (-14·4–2·9)
Southern Sub-Saharan Africa	1060 (892–1210)	95·2 (91·9–98·2)	85·4 (73·5–97·7)	-10·3 (-11·6–-9·1)	6810 (4940–8890)	118·0 (109·0–127·0)	527 (382–681)	-0·8 (-4·9–3·3)
Tropical Latin America	5230 (4480–5960)	158·0 (153·0–163·0)	96·6 (82·7–110)	-11·8 (-12·9–-10·8)	8000 (5580–10900)	174·0 (160·0–187·0)	149 (104–203)	-8·0 (-11·1–-5·1)
Western Europe	7970 (6770–9150)	57·8 (53·2–62·2)	36·3 (31·0–41·3)	-5·6 (-6·9–-4·4)	4190 (2770–5990)	54·5 (47·4–63·4)	20·3 (13·2–28·8)	-3·4 (-6·4–0·3)
Western Sub-Saharan Africa	4270 (3610–4970)	105·0 (102·0–108·0)	105 (90·2–121)	-8·1 (-8·8–-7·3)	14600 (10400–19500)	135·0 (127·0–142·0)	351 (252–463)	3·7 (1·5–5·9)
Global	143000 (122000–163000)	121·6 (118·6–124·3)	77·2 (66·2–88·2)	-0·3 (-0.8–-0·2)	419000 (295000–562000)	127·1 (122·7–131·9)	223 (158–299)	4·1 (2·7–5.5)

Table 4. Number of persons and crude prevalence of persons blind, with MSVI, with mild VI, and vision impairment from uncorrected presbyopia in 1990

	Mild vision impairment		Moderate and severe vision impairment		Blindness		Vision impairment due to uncorrected presbyopia	
Region	Cases in 1000s (95% UI)	Age-standardized rate per 1,000 (95% UI)	Cases in 1000s (95% UI)	Age-standardized rate per 1,000 (95% UI)	Cases in 1000s (95% UI)	Age-standardized rate per 1,000 (95% UI)	Cases in 1000s (95% UI)	Age-standardized rate per 1,000 (95% UI)
Andean Latin America	1100 (996–1230)	38·5 (34·5–42·7)	1270 (1150–1400)	47·5 (43·1–52·0)	213 (183–241)	10·2 (8·67–11·8)	1000 (720–1340)	49·4 (35·4–66·6)
Australasia	249 (224–276)	12·0 (10·8–13·3)	422 (382–462)	20.1 (18·2–22·0)	40·3 (35·1–45·3)	1·83 (1·60–2·06)	204 (158–256)	8·80 (6·83–11·1)
Caribbean	1160 (1050–1280)	38·3 (34·6–42·4)	950 (860–1050)	31·8 (28·8–34·9)	181 (154–206)	6·85 (5·82–7·82)	1410 (1010–1890)	54·2 (38·9–72·3)
Central Asia	1650 (1490–1830)	27·6 (25·0–30·6)	1970 (1790–2160)	38·2 (34·5–41·9)	247 (210–283)	5·37 (4·56–6·19)	3100 (2210–4100)	68·0 (48·5–89·8)
Central Europe	1920 (1730–2140)	15.0 (13·5–16·7)	3030 (2700–3400)	22·2 (20·0–24·6)	283 (242–322)	2·10 (1·81–2·37)	8840 (6300–11800)	60·9 (43·8–80·9)
Central Latin America	4770 (4310–5300)	39·5 (35·6–43·8)	4574 (4130–5050)	40·8 (36·9–44·7)	719 (629–805)	8·30 (7·17–9·38)	4570 (3300–6070)	55·8 (40·3–74·8)
Central Sub-Saharan Africa	1790 (1600–2000)	42·7 (38·4–47·4)	850 (763–948)	29·8 (26·7–33·3)	153 (131–174)	6·55 (5·56–7·49)	2040 (1460–2700)	88·0 (62·8–115)
East Asia	39200 (35300–43600)	38·0 (34·2–42·0)	22100 (19800–24700)	25·1 (22·5–27·7)	5310 (4770–5880)	6·22 (5·55–6·87)	66500 (47600–87800)	75·5 (54·6–99·7)
Eastern Europe	5210 (4680–5800)	21·6 (19·5–23·9)	9530 (8510–10600)	37·0 (33·3–40·9)	817 (708–923)	3·25 (2·83–3·65)	18900 (13500–24900)	68·9 (49·3–90·4)
Eastern Sub-Saharan Africa	5840 (5230–6560)	40·6 (36·6–45·0)	3260 (2960–3580)	34·9 (31·5–38·3)	1150 (1010–1290)	14·5 (12·5–16·4)	8070 (5990–10300)	103 (77·5–131)
High-income Asia Pacific	6190 (5580–6880)	35·3 (32·0–39·1)	3160 (2860–3460)	17·6 (15·9–19·3)	373 (328–420)	1·98 (1·75–2·22)	901 (602–1270)	4·56 (3·08–6·40)
High-income North America	3520 (3160–3910)	11·6 (10·4–12·9)	4950 (4480–5420)	15·9 (14·4–17·5)	416 (367–467)	1·23 (1·09–1·39)	2780 (1960–3770)	7.96 (5·68–10·8)
North Africa and Middle East	7990 (7190–8920)	29·8 (27·0–32·9)	10400 (9470–11500)	45·9 (41·8–50·1)	2020 (1740–2300)	12·0 (10·1–13·8)	6130 (4360–8290)	37·3 (26·7–50·2)
Oceania	189 (169–212)	39·1 (35·1–43·1)	173 (156–192)	49·7 (44·9–55·1)	22·3 (19–25·3)	7·28 (6·19–8·30)	179 (127–237)	61·8 (44·1–82·6)
South Asia	34500 (31200–38300)	43·6 (39·3–48·4)	48900 (44100–54000)	74·5 (67·1–82·4)	8700 (7560–9730)	16·8 (14·6–18·9)	70700 (51600–92300)	115 (84·3–149)
Southeast Asia	20400 (18400–22900)	54·4 (49.0–60·1)	15000 (13600–16400)	50.1 (45·5–54·8)	4070 (3570–4560)	16·4 (14·3–18·4)	12900 (9180–17200)	52·5 (37·9–70·5)
Southern Latin America	890 (803–990)	18·7 (16·8–20·7)	1350 (1230–1480)	28·7 (26·1–31·4)	119 (102–136)	2·70 (2·31–3·08)	279 (189–396)	6·14 (4·19–8·73)
Southern Sub-Saharan Africa	1650 (1480–1840)	39·4 (35·6–43·6)	869 (788–957)	24·6 (22·3–27·0)	328 (290–365)	11·5 (10·1–12·9)	4230 (3130–5390)	145 (108–184)
Tropical Latin America	4790 (4340–5310)	40·4 (36·4–44·8)	5500 (4950–6070)	45·7 (41·5–49·8)	932 (823–1040)	10·2 (8·95–11·4)	3380 (2430–4590)	38·9 (28.1–52·6)
Western Europe	8220 (7340–9140)	18·2 (16·4–20·2)	11300 (10200–12400)	24·4 (22·1–26·7)	1240 (1070–1400)	2·34 (2·04–2·63)	2980 (1980–4190)	5·26 (3·55–7·39)
Western Sub-Saharan Africa	7120 (6400–7940)	47·3 (42·8–52·4)	4390 (3980–4830)	42·0 (37·7–46·4)	1390 (1210–1560)	15·3 (13·1–17·3)	8070 (5890–10500)	88·3 (64·5–115)
Global	158000 (143000–176000)	33·3 (30·1–36·9)	154000 (140000–169000)	35·4 (32·1–38·8)	28700 (25300–31900)	7·18 (6·31–8·02)	227000 (164000–298000)	56·1 (40·8–73·4)

Table 5A. Forecasted number of persons and age-standardized prevalence of moderate and severe vision impairment and blindness in 2050 by region

	Moderate and severe vision impairment				Blindness			
Region	Female: Cases in 1000s (95% UI)	Female: Age-standardized rate per 1,000 (95% UI)	Male: Cases in 1000s (95% UI)	Male: Age-standardized rate per 1,000 (95% UI)	Female: Cases in 1000s (95% UI)	Female: Age-standardized rate per 1,000 (95% UI)	Male: Cases in 1000s (95% UI)	Male: Age-standardized rate per 1,000 (95% UI)
Andean Latin America	2890 (2620–3170)	45·5 (41·2–50·1)	2440 (2200–2670)	41·3 (37·2–45·4)	247 (211–285)	3·24 (2·77–3·7)	224 (191–259)	3·34 (2·85–3·84)
Australasia	825 (721–932)	21·9 (19·4–24·7)	696 (607–784)	21·6 (19.0–24·2)	67·8 (55·3–81·6)	1·37 (1·14–1·60)	51·9 (41·7–62·6)	1·33 (1·08–1·57)
Caribbean	1410 (1260–1540)	32.0 (29–35·1)	1020 (919–1130)	28.0 (25·3–30·7)	197 (168–229)	3·57 (3·08–4·08)	165 (141–190)	3·92 (3·34–4·48)
Central Asia	2990 (2680–3320)	35·1 (31·6–38·7)	2060 (1850–2290)	31·2 (28·3–34·3)	291 (246–335)	3·05 (2·61–3·49)	215 (182–247)	3·26 (2·78–3·73)
Central Europe	2660 (2340–2990)	22·1 (19·8–24·4)	1880 (1650–2110)	19·2 (17·2–21·2)	241 (202–285)	1·48 (1·27–1·69)	186 (157–217)	1·55 (1·33–1·77)
Central Latin America	10600 (9480–11600)	39·1 (35·3–42·8)	7880 (7090–8640)	34·2 (31·1–37·5)	1020 (883–1170)	3·08 (2·67–3·49)	852 (732–972)	3·22 (2·78–3·65)
Central Sub-Saharan Africa	2550 (2290–2830)	24·8 (22·3–27·5)	2360 (2110–2630)	25·7 (23.0–28·5)	348 (297–398)	3·74 (3·20–4·28)	290 (248–330)	3·66 (3·12–4·18)
East Asia	48900 (43100–54500)	30.0 (27·1–33·2)	33200 (29300–37000)	23·9 (21·6–26·4)	8210 (6830–9710)	3·88 (3·29–4·50)	6120 (5090–7320)	3·67 (3·10–4·31)
Eastern Europe	8020 (7150–8900)	36·9 (33·4–40·4)	4490 (4010–4960)	31·2 (28·2–34·3)	548 (467–641)	1·87 (1·61–2·12)	339 (292–389)	1·97 (1·71–2·24)
Eastern Sub-Saharan Africa	9740 (8870–10600)	28·1 (25·6–30·6)	7960 (7250–8710)	26·3 (24.0–28·6)	2380 (2090–2660)	7·40 (6·48–8·27)	1830 (1610–2040)	6·83 (5·97–7·65)
High-income Asia Pacific	4610 (4050–5170)	18·8 (16·9–20·7)	3950 (3490–4400)	19·2 (17·4–21·1)	421 (359–498)	1·18 (1·03–1·33)	348 (297–408)	1·31 (1·15–1·49)
High-income North America	7310 (6490–8120)	17·8 (16·1–19·6)	5580 (4950–6180)	15·6 (14·1–17·1)	733 (623–863)	1·38 (1·20–1·57)	610 (523–706)	1·42 (1·24–1·61)
North Africa and Middle East	24600 (22100–27100)	40·7 (36·8–44·7)	22100 (19900–24400)	38.0 (34·4–41·6)	2690 (2300–3100)	3·90 (3·35–4·48)	2270 (1940–2610)	3·62 (3·12–4·14)
Oceania	432 (387–475)	44·4 (40·1–48·5)	380 (343–421)	42·3 (38·2–46·6)	41·6 (35·7–47·5)	4·75 (4·07–5·41)	30·2 (25·8–34·7)	3·91 (3·35–4·46)
South Asia	67000 (60500–73700)	46·9 (42·7–51·1)	59100 (53400–65100)	43·2 (39·2–47·3)	6030 (5220–6890)	3·93 (3·44–4·44)	5610 (4860–6340)	3·89 (3·39–4·36)
Southeast Asia	26400 (24700–28500)	41·7 (39–44·7)	20700 (19200–22300)	39·1 (36·4–41·8)	4250 (3690–4850)	5·66 (4·94–6·38)	2810 (2430–3190)	4·81 (4·17–5·42)
Southern Latin America	2080 (1860–2300)	30.0 (27.0–33.0)	1620 (1440–1800)	28·3 (25·5–31·2)	123 (104–145)	1·45 (1·22–1·68)	104 (87·3–121)	1·59 (1·34–1·84)
Southern Sub-Saharan Africa	1680 (1500–1850)	23·7 (21·4–26·1)	1320 (1180–1470)	23·9 (21·5–26·4)	396 (340–449)	5·33 (4·60–5·99)	313 (269–354)	5·82 (5·03–6·54)
Tropical Latin America	11700 (10500–12800)	49·3 (44·7–53·9)	8550 (7700–9390)	44·1 (40·1–48·1)	1630 (1400–1870)	5·25 (4·59–5·89)	1220 (1060–1390)	5·25 (4·6–5·88)
Western Europe	13100 (11600–14600)	25.0 (22·5–27·4)	11400 (10100–12600)	25.0 (22·5–27·4)	1120 (942–1310)	1·56 (1·34–1·78)	744 (626–874)	1·29 (1·10–1·47)
Western Sub-Saharan Africa	14200 (12900–15600)	35·2 (31·9–38·7)	11300 (10300–12400)	31·5 (28·5–34·6)	2690 (2340–3020)	7·30 (6·32–8·24)	2270 (1980–2560)	7·29 (6·30–8·25)

Table 5B. Forecasted number of persons and age-standardized prevalence of mild vision impairment and vision impairment from uncorrected presbyopia in 2050 by region

	Mild vision impairment				Vision impairment due to uncorrected presbyopia			
Region	Female: Cases in 1000s (95% UI)	Female: Age-standardized rate per 1,000 (95% UI)	Male: Cases in 1000s (95% UI)	Male: Age-standardized rate per 1,000 (95% UI)	Female: Cases in 1000s (95% UI)	Female: Age-standardized rate per 1,000 (95% UI)	Male: Cases in 1000s (95% UI)	Male: Age-standardized rate per 1,000 (95% UI)
Andean Latin America	1900 (1680–2110)	32·7 (29·2–36·6)	1610 (1440–1780)	29·4 (26·4–32·7)	3070 (2140–4220)	43·4 (30·4–59·2)	2820 (1980–3890)	43·7 (30·7–59·6)
Australasia	399 (346–451)	12·3 (10·9–13·8)	302 (264–341)	10·7 (9·46–12.0)	365 (226–579)	8·05 (5·04–12·1)	286 (182–446)	7·57 (4·82–11·3)
Caribbean	1200 (1070–1340)	31·6 (28·3–35·1)	930 (829–1030)	28·6 (25·8–31·7)	2190 (1520–2990)	43·4 (30·4–59·2)	1810 (1270–2490)	43·7 (30·7–59·6)
Central Asia	1960 (1750–2180)	25.0 (22·5–27·6)	1450 (1290–1610)	22·7 (20·5–25·1)	6010 (4200–8160)	63·9 (44·9–86·9)	3970 (2760–5450)	55·5 (39·2–75·9)
Central Europe	1290 (1120–1460)	12·7 (11·4–14·2)	963 (835–1080)	11·6 (10·4–12·8)	8690 (6130–11800)	63·9 (44·9–86·9)	6310 (4410–8570)	55·5 (39·2–75·9)
Central Latin America	7730 (6860–8570)	32·4 (29·2–35·7)	6070 (5420–6710)	29·3 (26·4–32·6)	13400 (9320–18400)	43·4 (30·4–59·2)	11200 (7850–15400)	43·7 (30·7–59·6)
Central Sub-Saharan Africa	3850 (3460–4300)	35·4 (31·8–39·4)	3040 (2730–3410)	30·1 (27·1–33·4)	8760 (6530–11400)	90·6 (67·9–117)	7090 (5180–9290)	85·3 (63.0–111)
East Asia	42200 (36700–47500)	31·4 (28·2–34·7)	29700 (26000–33300)	25·5 (22·9–28·2)	105000 (75200–144000)	54·4 (38·7–74)	80200 (56100–109000)	48·5 (34·6–65·2)
Eastern Europe	3740 (3270–4200)	20·1 (18.0–22·2)	2320 (2050–2600)	18·3 (16·4–20·3)	16000 (11300–21600)	63·9 (44·9–86·9)	9200 (6410–12600)	55·5 (39·2–75·9)
Eastern Sub-Saharan Africa	11100 (10100–12300)	30·5 (27·7–33·8)	8720 (7870–9670)	26·3 (23·7–29.0)	30300 (22500–39600)	90·6 (67·9–117)	23800 (17400–31100)	85·3 (63.0–111)
High-income Asia Pacific	6420 (5620–7180)	34.0 (30·8–37·5)	4780 (4180–5370)	29·3 (26·5–32·6)	2460 (1500–4040)	8·05 (5·04–12·1)	1920 (1200–3070)	7·57 (4·82–11·3)
High-income North America	4000 (3500–4490)	11·4 (10·3–12·7)	3440 (3030–3830)	11·1 (10.0–12·4)	3940 (2440–6240)	8·05 (5·04–12·1)	3160 (2010–4920)	7·57 (4·82–11·3)
North Africa and Middle East	13800 (12300–15300)	24·7 (22·3–27·5)	12400 (11000–13800)	22·7 (20·4–25·2)	20500 (14200–28500)	30·3 (21.0–42·2)	17700 (12100–24900)	27·2 (18·8–38·2)
Oceania	349 (313–392)	34·4 (30·9–38·5)	291 (260–326)	30.0 (26·9–33·5)	498 (353–669)	54·4 (38·7–74.0)	382 (271–517)	48·5 (34·6–65·2)
South Asia	43800 (39000–48900)	33·9 (30·6–37·7)	37500 (33500–41900)	30.0 (27·1–33·3)	160000 (118000–206000)	98.0 (73·2–126)	139000 (103000–183000)	89·7 (66·7–117)
Southeast Asia	22000 (19800–24100)	39·2 (35·6–43·1)	18000 (16200–19900)	37·4 (33·9–41·3)	39600 (28200–54200)	54·4 (38·7–74)	29300 (20600–39400)	48·5 (34·6–65·2)
Southern Latin America	1060 (926–1190)	17·3 (15·5–19·3)	805 (709–896)	15·6 (14.0–17·4)	673 (418–1050)	8·05 (5·04–12·1)	508 (318–771)	7·57 (4·82–11·3)
Southern Sub-Saharan Africa	2180 (1950–2430)	33·1 (29·7–36·7)	1530 (1370–1690)	28·1 (25·3–31·2)	6860 (5140–8940)	90·6 (67·9–117)	5020 (3650–6550)	85·3 (63.0–111)
Tropical Latin America	6850 (6030–7690)	33·2 (29·9–37.0)	5110 (4520–5710)	29·9 (26·9–33·1)	12400 (8590–17100)	43·4 (30·4–59·2)	10000 (7010–13800)	43·7 (30·7–59·6)
Western Europe	7670 (6680–8610)	17·5 (15·8–19·5)	6190 (5400–6920)	16 (14·3–17·7)	5220 (3210–8410)	8·05 (5·04–12·1)	4210 (2640–6700)	7·57 (4·82–11·3)
Western Sub-Saharan Africa	18000 (16300–19900)	41·4 (37·7–45·5)	13500 (12200–15000)	33·3 (30·2–36·8)	33600 (25100–43800)	90·6 (67·9–117)	26600 (19400–34400)	85·3 (63.0–111)

Figure 1: Data Sources used in the analysis from RAAB (Map A) and non-RAAB (Map B) studies.

Map A.

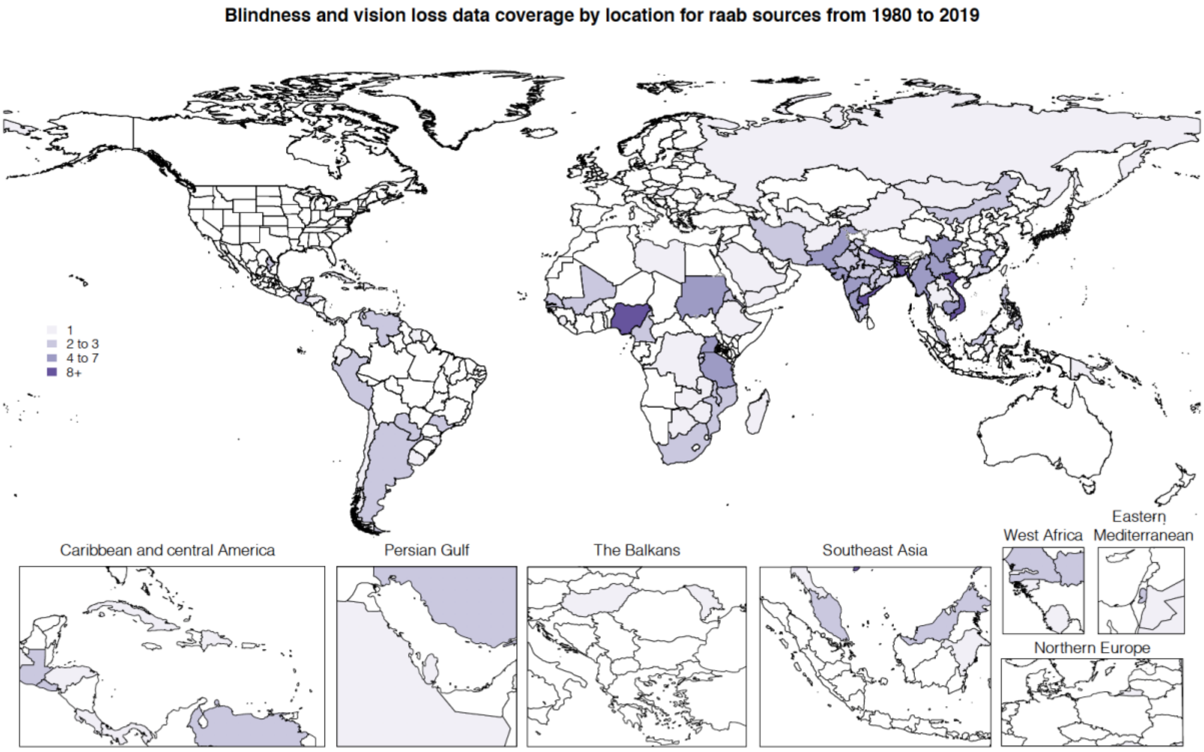

Map B.

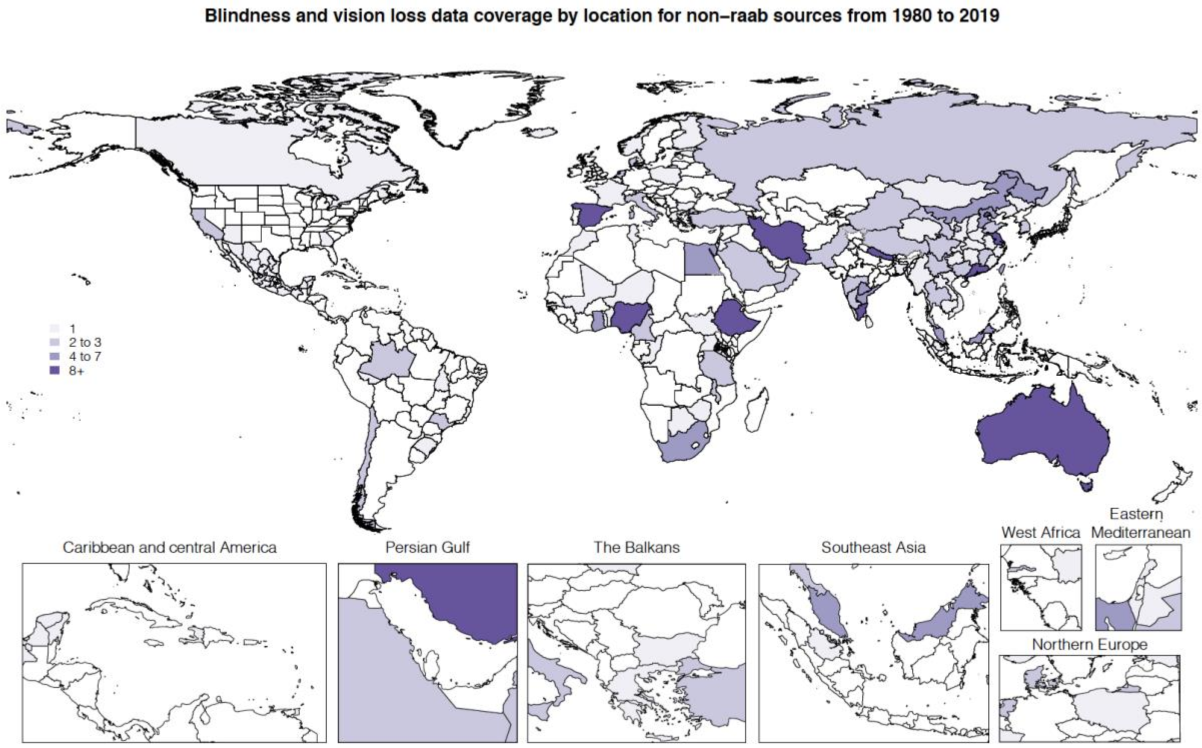

All sources can be found in Tables 2 and 3 of Appendix 1.

Figure 2 A-D. Forecast of numbers of people affected by blindness, moderate and severe vision impairment, mild vision impairment and vision impairment from uncorrected presbyopia to 2050- by region.

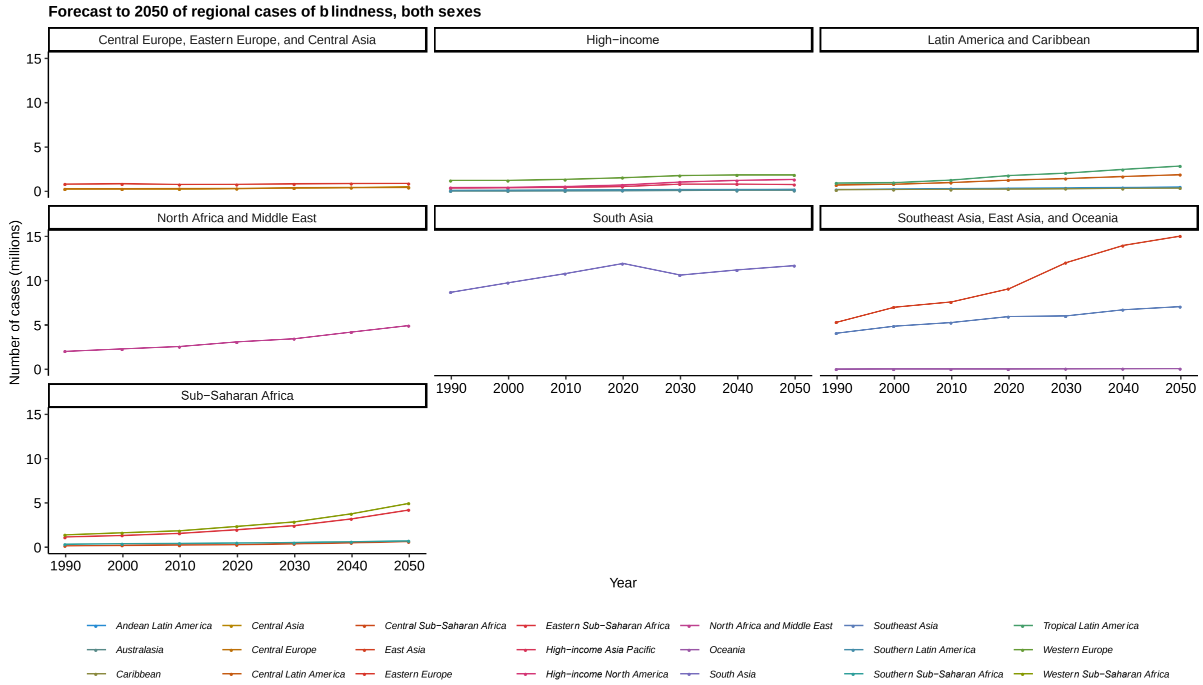

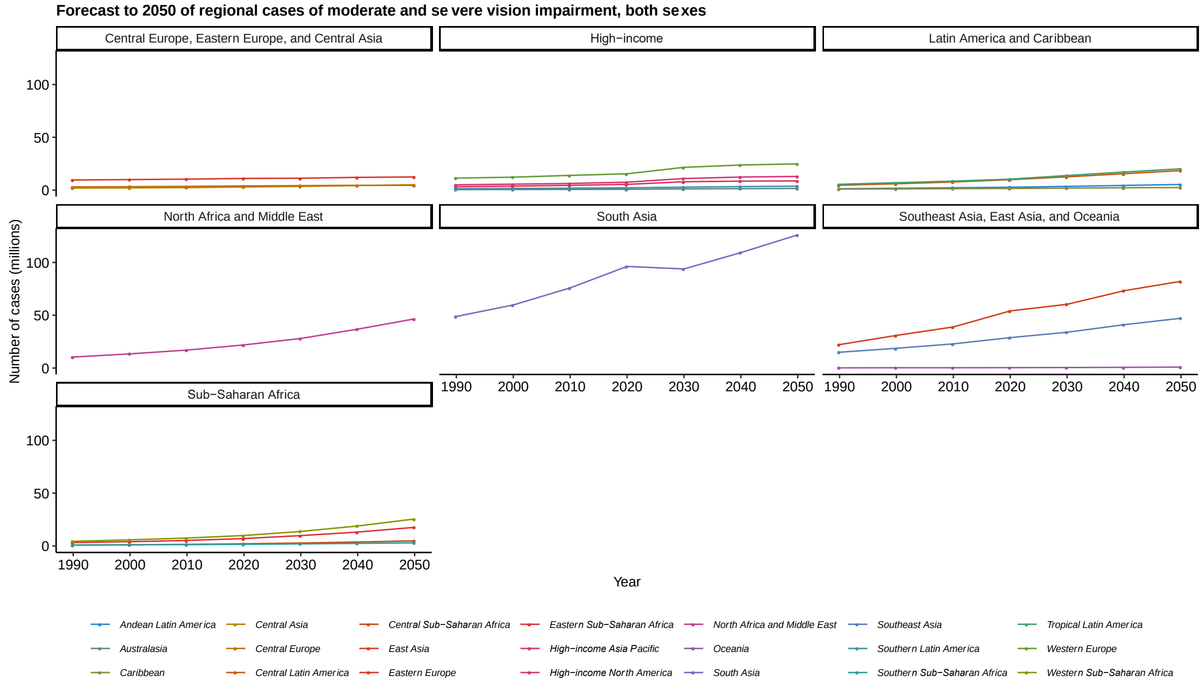

Forecast to 2050 of regional cases of mild vision impairment, both se xes

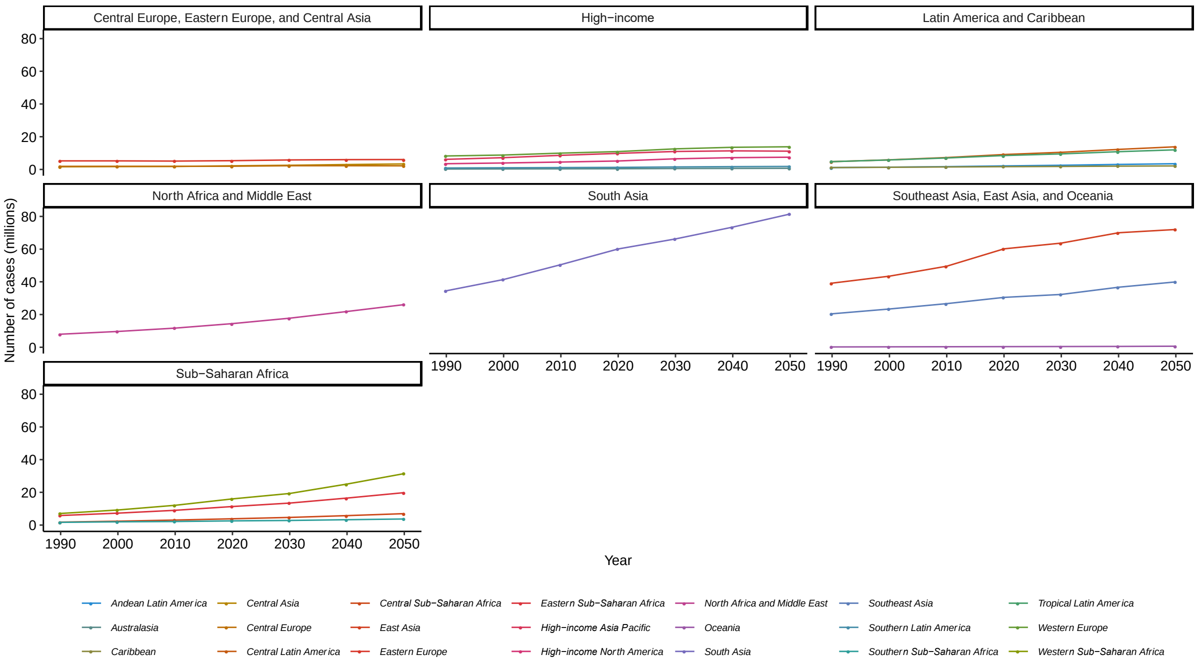

Forecast to 2050 of regional cases of vision impairment fr om uncorrected presbyopia, both sexes

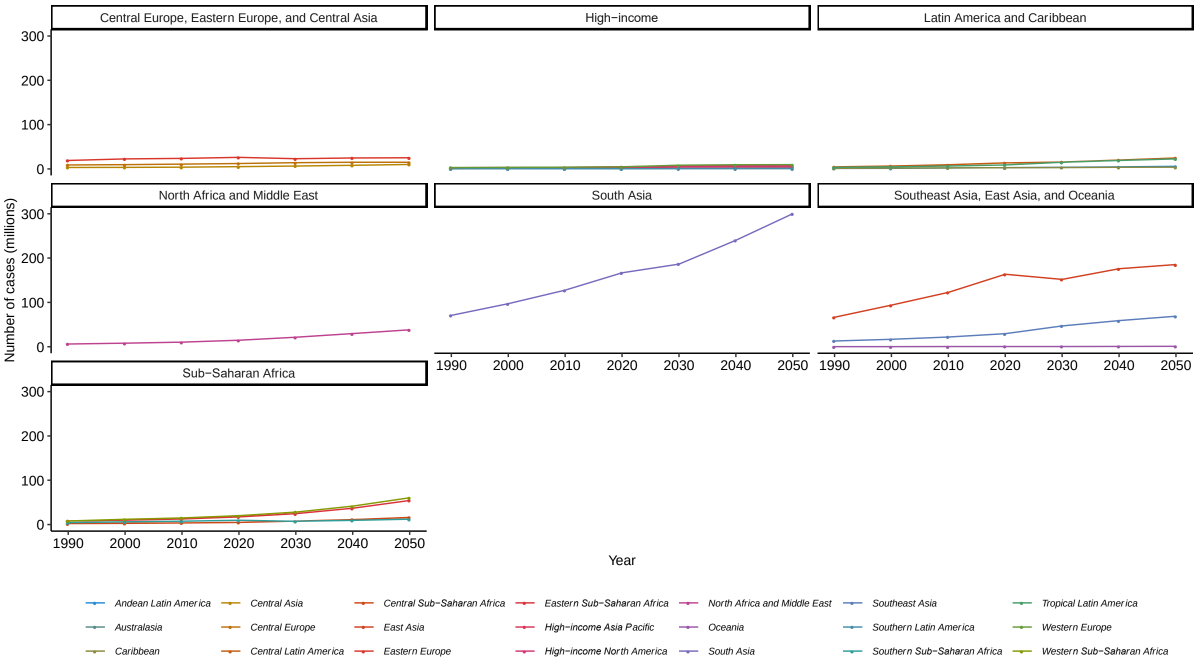

APPENDIX 3: CONTRIBUTORS

GBD 2019 Blindness and Vision Impairment Collaborator Contributions

Managing the estimation process
Rupert Bourne and Theo Vos.
Writing the first draft of the manuscript
Rupert Bourne, Hugh Taylor, Serge Resnikoff, Robert Casson, and Jost Jonas.
Providing data or critical feedback on data sources
Rupert Bourne, Hugh Taylor, Serge Resnikoff, Kenji Shibuya, Amir Abdoli, Yonas Akalu, Jalal Arabloo, Till Bärnighausen, Akshaya Bhagavathula, Mukharram Bikbov, Michele Bottone, Tasanee Braithwaite, Alain Bron, Ching-Yu Cheng, Dinh-Toi Chu, Maria Vittoria Cicinelli, Lalit Dandona, Rakhi Dandona, Jenny Deva, Joshua Ehrlich, Leon Ellwein, Mohammad Hassan Emamian, Arthur Fernandes, João Furtado, Abhay Gaidhane, Shilpa Gaidhane, Gus Gazzard, Ronnie George, Ahmad Ghashghaee, Mahaveer Golechha, Mary Elizabeth Hartnett, Hung Chak Ho, Chi Hoang, Mowafa Househ, Seyed Sina Irvani, Rim Kahloun, Ayele Semachew Kasa, Maryam Keramati, Moncef Khairallah, Ejaz Khan, Rohit Khanna, Mahalaqua Nazli Khatib, Judy Kim, Yun Jin Kim, Adnan Kisa, Sezer Kisa, Van Lansingh, Janet Leasher, Nicolas Leveziel, Hans Limburg, Navid Manafi, Colm McAlinden, Seyed Farzad Mohammadi, Abdollah Mohammadian-Hafshejani, Reza Mohammadpourhodki, Ali Mokdad, Delaram Moosavi, Kovin Naidoo, Vinay Nangia, Cuong Nguyen, Huong Nguyen, Kolawole Ogundimu, Andrew Olagunju, Songhomitra Panda-Jonas, Konrad Pesudovs, Tunde Peto, Zahiruddin Quazi Syed, Mohammad Hifz Ur Rahman, David Rawaf, Salman Rawaf, Sare Safi, Abdallah Samy, Deepak Saxena, Masood Shaikh, Tueng Shen, Jae Il Shin, Juan Carlos Silva, Alexander Silvester, Jasvinder Singh, Deepika Singhal, Rita Sitorus, Eirini Skiadaresi, Amin Soheili, Eyayou Tadesse, Nina Tahhan, Bach Tran, Ravensara Travillian, Ningli Wang, Tien Wong, Zoubida Zaidi, and Jost Jonas.
Development of methods or computational machinery
Ashkan Afshin, Akshaya Bhagavathula, Michele Bottone, Mowafa Househ, Maryam Keramati, Mahalaqua Nazli Khatib, Adnan Kisa, Sezer Kisa, Reza Mohammadpourhodki, Ali Mokdad, Konrad Pesudovs, Zahiruddin Quazi Syed, Abdallah Samy, and Jost Jonas.
Providing critical feedback on methods or results
Hugh Taylor, Eman Abu-Gharbieh, Ashkan Afshin, Hamid Ahmadieh, Yonas Akalu, Alehegn Alamneh, Ahmed Alfaar, Vahid Alipour, Etsay Anbesu, Sofia Androudi, Jalal Arabloo, Eleni Bagli, Atif Baig, Till Bärnighausen, Akshaya Bhagavathula, Pankaj Bhardwaj, Nikha Bhardwaj, Krittika Bhattacharyya, Ali Bijani, Michele Bottone, Zahid Butt, Dinh-Toi Chu, João Coelho, Baye Dagnew, Reza Dana, Lalit Dandona, Rakhi Dandona, Daniel Diaz, Shirin Djalalinia, Joshua Ehrlich, Mohammad Hassan Emamian, Florian Fischer, Abhay Gaidhane, Shilpa Gaidhane, Berhe Gebremichael, Ahmad Ghashghaee, Mahaveer Golechha, Risky Hartono, Simon Hay, Golnaz Heidari, Hung Chak Ho, Chi Hoang, Mowafa Househ, Segun Ibitoye, Milena Ilic, Irena Ilic, Seyed Sina Irvani, Ravi Jha, Himal Kandel, Ayele Semachew Kasa, Maryam Keramati, Ejaz Khan, Mahalaqua Nazli Khatib, Yun Jin Kim, Adnan Kisa, Sezer Kisa, Ai Koyanagi, Om Kurmi, Nicolas Leveziel, Marek Majdan, Navid Manafi, Colm McAlinden, Seyed Farzad Mohammadi, Abdollah Mohammadian-Hafshejani, Reza Mohammadpourhodki, Ali Mokdad, Delaram Moosavi, Mehdi Naderi, Kovin Naidoo, Cuong Nguyen, Huong Nguyen, Andrew Olagunju, Samuel Ostroff, Songhomitra Panda-Jonas, Konrad Pesudovs, Zahiruddin Quazi Syed, Mohammad Hifz Ur Rahman, David Rawaf, Salman Rawaf, Sare Safi, Abdallah Samy, Deepak Saxena, Masood Shaikh, Jae Il Shin, Jasvinder Singh, Deepika Singhal, Rita Sitorus, Eirini Skiadaresi, Vegard Skirbekk, Amin Soheili, Raúl Sousa, Emma Spurlock, Biruk Taddele, Eyayou Tadesse, Md Tareque, Fotis Topouzis, Bach Tran, Ravensara Travillian, Miltiadis Tsilimbaris, Zoubida Zaidi, Kaleab Zewdie, and Jost Jonas.
Extracting, cleaning, or cataloging data; designing or coding figures and tables
Rupert Bourne, Jaimie D Steinmetz, Seth Flaxman, Paul Briant, Serge Resnikoff, Mukharram Bikbov, Michele Bottone, Tasanee Braithwaite, Alain Bron, Ching-Yu Cheng, Maria Vittoria Cicinelli, Arthur Fernandes, João Furtado, Ronnie George, Rim Kahloun, Moncef Khairallah, Judy Kim, Van Lansingh, Janet Leasher, Hans Limburg, Vinay Nangia, Kolawole Ogundimu, Konrad Pesudovs, Tunde Peto, Nickolas Reinig, Alexander Silvester, Nina Tahhan, Ningli Wang, Tien Wong, Jost Jonas, and Theo Vos.
Drafting the manuscript or revising it critically for important intellectual content
Rupert Bourne, Jaimie D Steinmetz, Seth Flaxman, Paul Briant, Hugh Taylor, Serge Resnikoff, Robert Casson, Eman Abu-Gharbieh, Wondu Alemayehu, Ahmed Alfaar, Sofia Androudi, Jalal Arabloo, Malke Asaad, Eleni Bagli, Atif Baig, Till Bärnighausen, Maurizio Battaglia Parodi, Akshaya Bhagavathula, Krittika Bhattacharyya, Mukharram Bikbov, Michele Bottone, Tasanee Braithwaite, Alain Bron, Ching-Yu Cheng, Maria Vittoria Cicinelli, João Coelho, Baye Dagnew, Xiaochen Dai, Monte Del Monte, Daniel Diaz, Joshua Ehrlich, Mohammad Hassan Emamian, Arthur Fernandes, Florian Fischer, David Friedman, João Furtado, Abhay Gaidhane, Shilpa Gaidhane, Gus Gazzard, Berhe Gebremichael, Ronnie George, Ahmad Ghashghaee, Samer

Hamidi, Billy Hammond, Golnaz Heidari, Hung Chak Ho, Chi Hoang, Mowafa Househ, Segun Ibitoye, Milena Ilic, Irena Ilic, April Ingram, Seyed Sina Irvani, Ravi Jha, Rim Kahloun, Himal Kandel, John Kempen, Moncef Khairallah, Ejaz Khan, Rohit Khanna, Mahalaqua Nazli Khatib, Adnan Kisa, Ai Koyanagi, Om Kurmi, Van Lansingh, Janet Leasher, Nicolas Leveziel, Hans Limburg, Marek Majdan, Navid Manafi, Kaweh Mansouri, Colm McAlinden, Abdollah Mohammadian-Hafshejani, Ali Mokdad, Delaram Moosavi, Mehdi Naderi, Kovin Naidoo, Vinay Nangia, Cuong Nguyen, Huong Nguyen, Kolawole Ogundimu, Andrew Olagunju, Songhomitra Panda-Jonas, Konrad Pesudovs, Tunde Peto, Zahiruddin Quazi Syed, Mohammad Hifz Ur Rahman, Pradeep Ramulu, Nickolas Reinig, Alan Robin, Amirhossein Sahebkar, Abdallah Samy, Deepak Saxena, Janet Serle, Masood Shaikh, Kenji Shibuya, Jae Il Shin, Alexander Silvester, Jasvinder Singh, Deepika Singhal, Rita Sitorus, Eirini Skiadaresi, Raúl Sousa, Dwight Stambolian, Eyayou Tadesse, Nina Tahhan, Fotis Topouzis, Bach Tran, Ravensara Travillian, Rohit Varma, Gianni Virgili, Ningli Wang, Ya Xing Wang, Sheila West, Zoubida Zaidi, Kaleab Zewdie, Jost Jonas, and Theo Vos.

Management of the overall research enterprise

Rupert Bourne, Hugh Taylor, and Theo Vos.
